# Supplementary material for: Efficient Synthesis of 2-OH Thioglycosides from Glycals Based on the Reduction of Aryl Disulfides by NaBH4
Source: Molecules. 2022 Sep 14;27(18):5980. doi: 10.3390/molecules27185980 (PMC9506437; doi:10.3390/molecules27185980)
Supplement: Supplementary file 1 [file molecules-27-05980-s001.zip › molecules-1912277-supplementary.pdf]

Supplementary Material

Efficient Synthesis of 2-OH Thioglycosides from Glycals Based on the Reduction of Aryl Disulfides by $NaBH_4$

Yang-Fan Guo,[#] Tao Luo,[#] Guang-Jing Feng, Chun-Yang Liu, and Hai Dong*

Key laboratory of Material Chemistry for Energy Conversion and Storage, Ministry of Education, School of Chemistry & Chemical Engineering, Hubei Key Laboratory of Material Chemistry and Service Failure, Huazhong University of Science & Technology, Luoyu Road 1037, Wuhan, 430074, P. R. China. E-mail: hdong@mail.hust.edu.cn

TABLE OF CONTENTS

1. Table S1……………………………………………………………………………………..S2

2. Preparation of glycals…………………………………………………………………S3

3. Reference…………………………………………………………………………………..S5

4. Copy of NMR spectra …. ……………………………………………………………..S6

1. Table S1. Comparison of isolated yields with NMR yields.[a]

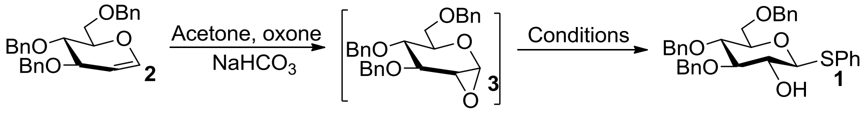

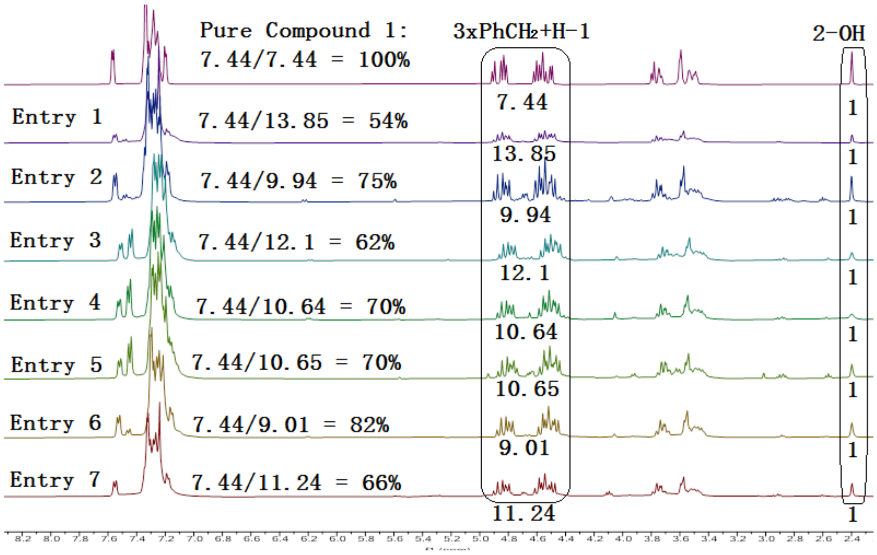

Entry	Reaction condition	**NMR**/Isolated Yields (%)
1	ACN, NaSPh (1.5eq), rt, 36 h	**54**/43
2	ACN, PhSSPh(0.7eq), $NaBH_4$(1.5eq), rt, 1h	**75**/73
3	ACN, PhSSPh(0.5eq), $NaBH_4$(1.0eq), rt, 1h	**62**/55
4	ACN, PhSSPh(0.7eq), $NaBH_4$(0.7eq), rt, 1h	**70**/60
5	ACN, PhSSPh(0.5eq), $NaBH_4$(0.5eq), NaSPh (0.4eq), rt, 4h	**70**/65
6	ACN, PhSSPh(0.3eq), $NaBH_4$(0.3eq), NaSPh (0.8eq), rt, 4h	**82**/72
7	ACN, PhSSPh(0.1eq), $NaBH_4$(0.1eq), NaSPh (1.2eq), rt, 4h	**66**/55

[a] Reagents and conditions: substrate **2** (0.1 mmol), solvents (1 mL), yields based on **2**.

2. Preparation of glycals

a) List of glycals

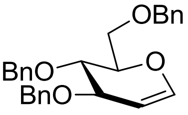

2

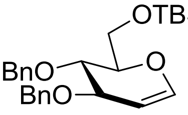

4a

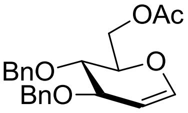

5a

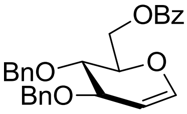

6a

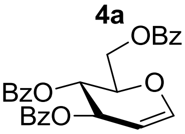

7a

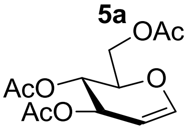

8a

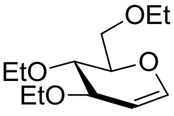

9a

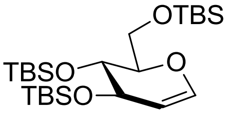

10a

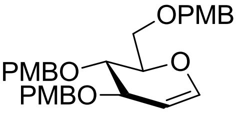

11a

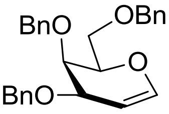

12a

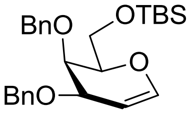

13a

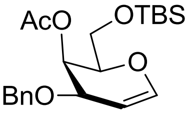

14a

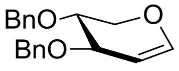

15a

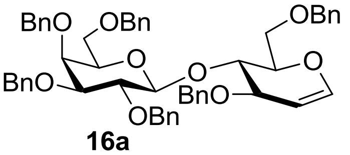

16a

b) Preparation of glycals

3,4,6-Tri-*O*-benzyl-α-D-glucal 2: Synthesized in light of the reported reference.[1] ^{1}H NMR (600 MHz, Chloroform-*d*) δ 7.37 – 7.25 (m, 16H), 6.45 (dd, *J* = 6.1, 1.4 Hz, 1H), 4.90 (dd, *J* = 6.2, 2.7 Hz, 1H), 4.86 (d, *J* = 11.3 Hz, 1H), 4.68 – 4.64 (m, 2H), 4.64 – 4.55 (m, 3H), 4.24 (ddd, *J* = 6.2, 2.8, 1.4 Hz, 1H), 4.09 (ddd, *J* = 8.3, 5.2, 2.8 Hz, 1H), 3.89 (dd, *J* = 8.7, 6.2 Hz, 1H), 3.83 – 3.75 (m, 2H) ppm.

3,4-Di-*O*-benzyl-6-*O*-*tert*-butyl-dimethylsily-D-glucal 4a: Synthesized in light of the reported reference.[2] ^{1}H NMR (400 MHz, Chloroform-*d*) δ 7.32 – 7.15 (m, 10H), 6.31 (d, *J* = 6.1, Hz, 1H), 4.82 – 4.73 (m, 2H), 4.67 (d, *J* = 11.2 Hz, 1H), 4.46 – 4.60 (m, 2H), 4.13 (dt, *J* = 4.1, 2.1 Hz, 1H), 3.90 (dd, *J* = 10.5, 2.7 Hz, 1H), 3.85 – 3.76 (m, 3H), 0.83 (s, 9H), 0.09 – 0.03 (m, 6H) ppm.

3,4-Di-*O*-benzyl-6-*O*-acetyl-α-D-glucal 5a: Synthesized in light of the reported reference.[3] ^{1}H NMR (400 MHz, Chloroform-*d*) δ 7.46 – 7.29 (m, 10H), 6.44 (dd, *J* = 6.2, 1.3 Hz, 1H), 4.97 (dd, *J* = 6.2, 2.7 Hz, 1H), 4.90 (d, *J* = 11.3 Hz, 1H), 4.74 – 4.68 (m, 2H), 4.60 (d, *J* = 11.7 Hz, 1H), 4.49 – 4.36 (m, 2H), 4.27 (ddd, *J* = 6.1, 2.8, 1.4 Hz, 1H), 4.14 (ddd, *J* = 8.3, 5.2, 2.8 Hz, 1H), 3.83 (dd, *J* = 8.6, 6.1 Hz, 1H), 2.09 (s, 3H) ppm.

3,4-Di-*O*-benzyl-6-*O*-benzoyl-α-D-glucal 6a: 3,4-Di-*O*-benzyl-D-glucal[4] (1.46 g, 4.47 mmol) was dissolved in a solution of pyridine (20 mL), and benzoyl chloride (1.2 eq) was added and stirred for 1h. The solution was then diluted with DCM (100 mL), washed with 1M HCl (2 x 60 mL), $NaHCO_3$ (sat. aq.) (2 x 60 mL), brine (40 mL), dried over $MgSO_4$ filtered and concentrated in vacuo. Following purification by column chromatography (petrol ether: EtOAc, 95:5 to 85:15)

led to product **6a** as a solid (1.52 g, 92%). ^{1}H NMR (400 MHz, Chloroform-*d*) δ 8.05 – 7.96 (m, 2H), 7.55 (t, *J* = 7.4 Hz, 1H), 7.46 – 7.37 (m, 2H), 7.38 – 7.16 (m, 10H), 6.42 (d, *J* = 6.1 Hz, 1H), 4.94 (dd, *J* = 6.2, 2.7 Hz, 1H), 4.88 (d, *J* = 11.2 Hz, 1H), 4.72 – 4.55 (m, 5H), 4.30 – 4.24 (m, 1H), 4.22 (dt, *J* = 8.1, 3.8 Hz, 1H), 3.91 (dd, *J* = 8.7, 6.1 Hz, 1H) ppm. ^{13}C NMR (100 MHz, Chloroform-*d*) δ 166.73, 144.10, 138.08, 137.74, 133.09, 129.89, 129.73, 128.51, 128.49, 128.39, 128.10, 127.93, 127.86, 127.82, 100.04, 75.73, 75.19, 74.04, 73.81, 70.76, 63.00 ppm. $[\alpha]^{20}_D$ = -53.0 (c 0.1, CH_2Cl_2); HRMS (ESI-TOF) (m/z): $[M + Na]^+$ calcd for $C_{27}H_{26}O_5Na^+$, 453.1678; found, 453.1648.

3,4,6-Tri-*O*-benzoyl-*α*-D-glucal 7a: Synthesized in light of the reported reference.[5] ^{1}H NMR (400 MHz, Chloroform-*d*) δ 8.11 – 8.00 (m, 6H), 7.67 – 7.50 (m, 3H), 7.49 – 7.36 (m, 6H), 6.63 (dd, *J* = 6.2, 1.3 Hz, 1H), 5.83 (dd, *J* = 7.3, 4.0 Hz, 1H), 5.78 – 5.71 (m, 1H), 5.15 (dd, *J* = 6.2, 3.5 Hz, 1H), 4.79 – 4.61 (m, 3H) ppm.

3,4,6-Tri-*O*-acetyl-D-glucal 8a: Synthesized in light of the reported reference.[6] ^{1}H NMR (400 MHz, Chloroform-*d*) δ 6.47 (dd, *J* = 6.2, 1.4 Hz, 1H), 5.35 (dd, *J* = 6.3, 3.2 Hz, 1H), 5.23 (dd, *J* = 7.6, 5.6 Hz, 1H), 4.85 (dd, *J* = 6.2, 3.3 Hz, 1H), 4.41 (dd, *J* = 12.0, 5.7 Hz, 1H), 4.30 – 4.16 (m, 2H), 2.14 – 2.06 (m, 6H), 2.05 (s, 3H) ppm.

3,4,6-Tri-*O*-ethyl-D-glucal 9a: Synthesized in light of the reported reference.[3] ^{1}H NMR (400 MHz, Chloroform-d) δ 6.38 (dd, J = 6.1, 1.5 Hz, 1H), 4.80 (dd, J = 6.1, 2.6 Hz, 1H), 4.02 – 3.89 (m, 2H), 3.84 (dq, J = 9.3, 7.0 Hz, 1H), 3.76 – 3.64 (m, 4H), 3.63 – 3.48 (m, 4H), 1.27 – 1.18 (m, 9H) ppm.

3,4,6-Tri-*O*-*tert*-butyl-dimethylsily-D-glucal 10a: Synthesized in light of the reported reference.[3] ^{1}H NMR (400 MHz, Chloroform-*d*) δ 6.23 (d, *J* = 6.3 Hz, 1H), 4.63 – 4.56 (m, 1H), 3.93 – 3.77 (m, 3H), 3.72 – 3.62 (m, 2H), 0.96 – 0.18 (m, 27H), 0.13 – 0.01 (m, 18H) ppm.

3,4,6-Tri-*O*-*p*-methoxybenzyl-*α*-D-glucal 11a: Synthesized in light of the reported reference.[3] ^{1}H NMR (400 MHz, Chloroform-d) δ 7.30 – 7.20 (m, 4H), 7.17 – 7.09 (m, 2H), 6.89 – 6.80 (m, 6H), 6.43 – 6.37 (m, 1H), 4.84 (dd, J = 6.1, 2.7 Hz, 1H), 4.73 (d, J = 10.9 Hz, 1H), 4.60 – 4.45 (m, 5H), 4.16 (dt, J = 6.3, 2.0 Hz, 1H), 4.01 (ddd, J = 8.4, 4.9, 2.9 Hz, 1H), 3.85 – 3.75 (m, 10H), 3.74 – 3.66 (m, 2H) ppm.

3,4,6-Tri-*O*-benzyl-*α*-D-galactal 12a: Synthesized in light of the reported reference.[5] ^{1}H NMR (400 MHz, Chloroform-*d*) δ 7.41 – 7.18 (m, 15H), 6.36 (d, *J* = 6.2 Hz, 1H), 4.90 – 4.80 (m, 2H), 4.69 – 4.56 (m, 3H), 4.52 – 4.35 (m, 2H), 4.20 – 4.15 (m, 2H), 3.94 (t, *J* = 3.1 Hz, 1H), 3.78 (dd, *J* = 10.1, 7.2 Hz, 1H), 3.64 (dd, *J* = 10.1, 5.0 Hz, 1H) ppm.

3,4-Di-*O*-benzyl-6-*O*-tert-butyl-dimethylsily-D-galactal 13a: Synthesized in light of the reported reference.[7] ^{1}H NMR (400 MHz, Chloroform-*d*) δ 7.44 – 7.20 (m, 10H), 6.34 (d, *J* = 6.2 Hz, 1H), 4.90 (d, *J* = 11.9 Hz, 1H), 4.86 – 4.81 (m, 1H), 4.70 – 4.60 (dd, *J* = 17.9, 10.3 Hz, 3H), 4.20 (s, 1H), 4.03 – 3.95 (m, 2H), 3.87 (dd, *J* = 10.7, 6.8 Hz, 1H), 3.81 (dd, *J* = 10.7, 6.0 Hz, 1H), 0.88 (s, 9H), 0.04 (s, 6H) ppm.

3-*O*-Benzyl-4-*O*-acetyl-6-*O*-tert-butyl-dimethylsily-D-galactal 14a: Synthesized in light of the reported reference.[8] ^{1}H NMR (400 MHz, Chloroform-*d*) δ 7.40 – 7.17 (m, 5H), 6.31 (dd, J = 6.3, 2.0 Hz, 1H), 5.55 (dt, J = 3.4, 1.7 Hz, 1H), 4.67 (dt, J = 6.4, 2.0 Hz, 1H), 4.63 (d, J = 11.7 Hz, 1H), 4.43 (d, J = 11.8 Hz, 1H), 4.19 (dd, J = 5.1, 2.5 Hz, 1H), 3.97 (t, J = 6.8 Hz, 1H), 3.70 (dd, J = 10.2, 6.2 Hz, 1H), 3.62 (dd, J = 10.2, 7.3 Hz, 1H), 2.07 (s, 3H), 0.84 (s, 9H), 0.00 (s, 6H) ppm.

3,4-Di-*O*-benzyl-D-xylal 15a: Synthesized in light of the reported reference.[9] ^{1}H NMR (400 MHz, Chloroform-*d*) δ 7.36 – 7.26 (m, 10H), 6.55 (d, *J* = 6.2 Hz, 1H), 4.93 (t, *J* = 5.5 Hz, 1H), 4.64

(s, 2H), 4.60 (d, *J* = 11.8 Hz, 1H), 4.52 (d, *J* = 11.8 Hz, 1H), 4.11 (dd, *J* = 11.7, 4.0 Hz, 1H), 3.95 (dd, *J* = 11.7, 2.0 Hz, 1H), 3.84 (d, *J* = 4.1 Hz, 1H), 3.67 (s, 1H) ppm.

2,3,3′,4,6,6′-Hexa-*O*-benzyl-D-lactal 16a: Synthesized in light of the reported reference.[9] ^{1}H NMR (400 MHz, Chloroform-*d*) δ 7.37 – 7.21 (m, 30H), 6.44 (d, *J* = 6.2 Hz, 1H), 4.93 (d, *J* = 11.6 Hz, 1H), 4.87 (dd, *J* = 6.3, 3.6 Hz, 1H), 4.83 (d, *J* = 10.8 Hz, 1H), 4.76 – 4.65 (m, 3H), 4.62 – 4.52 (m, 4H), 4.47 (s, 2H), 4.40 – 4.29 (m, 2H), 4.27 (q, *J* = 5.3 Hz, 1H), 4.16 – 4.06 (m, 2H), 3.90 – 3.72 (m, 3H), 3.67 (dd, *J* = 10.7, 3.5 Hz, 1H), 3.54 (t, *J* = 8.0 Hz, 1H), 3.51 – 3.39 (m, 3H) ppm.

3. Reference

(1) Chennaiah, A.; Verma, A. K.; Vankar, Y. D. *J. Org. Chem.* **2018**, *83*, 10535–10540.

(2) Lellouche, J.-P.; Koeller, S. *J. Org. Chem.* **2001**, *66*, 693-696.

(3) Balijepalli, A. S.; McNeely, J. H.; Hamoud, A.; Grinstaff, M. W. *J. Org. Chem.* **2020**, *85,* 12044–12057.

(4) Kim, Y.; Oh, K.; Song, H.; Lee, D.-S.; Park, S. B. *J. Med. Chem.* **2013**, *56*, 7100–7109.

(5) Bi, J.-J.; Tan, Q.; Wu, H.; Liu, Q.-F.; Zhang, G.-S. *Org. Lett.* **2021**, *23*, 6357–6361.

(6) Chen, H.; Xian, T.; Zhang, W.; Si, W.; Luo, X.; Zhang, B.; Zhang, M.; Wang, Z.; Zhang, J.-B. *Carbohydr. Res.* **2016**, *431*, 42–46.

(7) Aurrecoechea, J. M.; Arrate, M.; Gil, J. H.; Lopez, B. *Tetrahedron*. **2003**, *59*, 5515-5522.

(8) Bieg, T.; Kral, K.; Paszkowska, J.; Szeja, W.; Wandzik, I. *J. Carbohydr. Chem.* **2012**, *31,* 593-601.

(9) Liu, M.; Luo, Z.-X.; Li, T.; Xiong, D.-C.; Ye, X.-S. *J. Org. Chem*. **2021**, *86*, 16187-16194.

4. Copy of NMR Spectra

3,4,6-Tri-*O*-benzyl-α-D-glucal 2

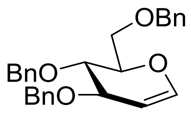

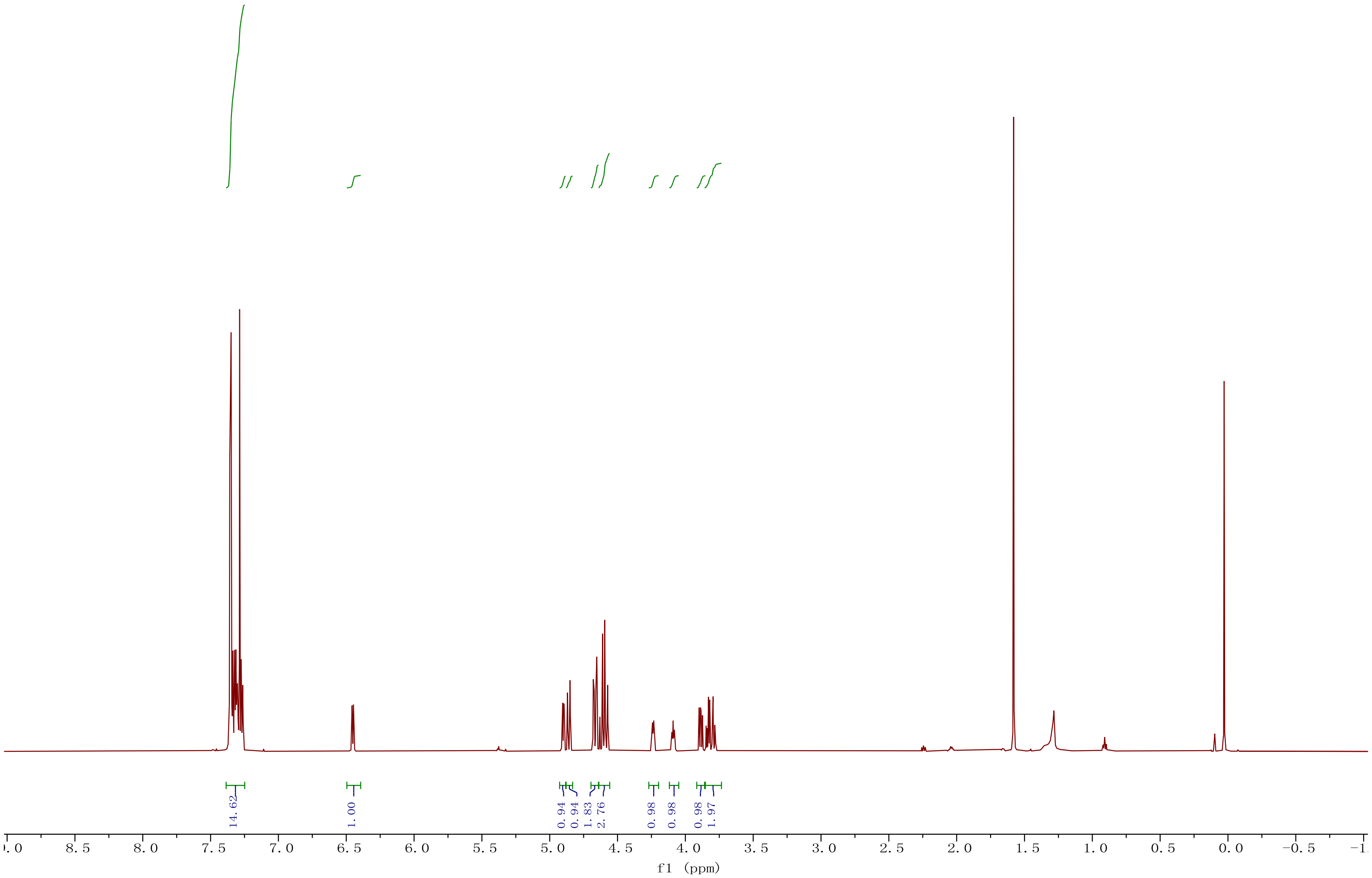

Figure S2. ^{1}H NMR spectrum (400 MHz) of **2** in $CDCl_3$

3,4-Di-*O*-benzyl-6-*O*-tert-butyl-dimethylsily-D-glucal 4a

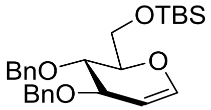

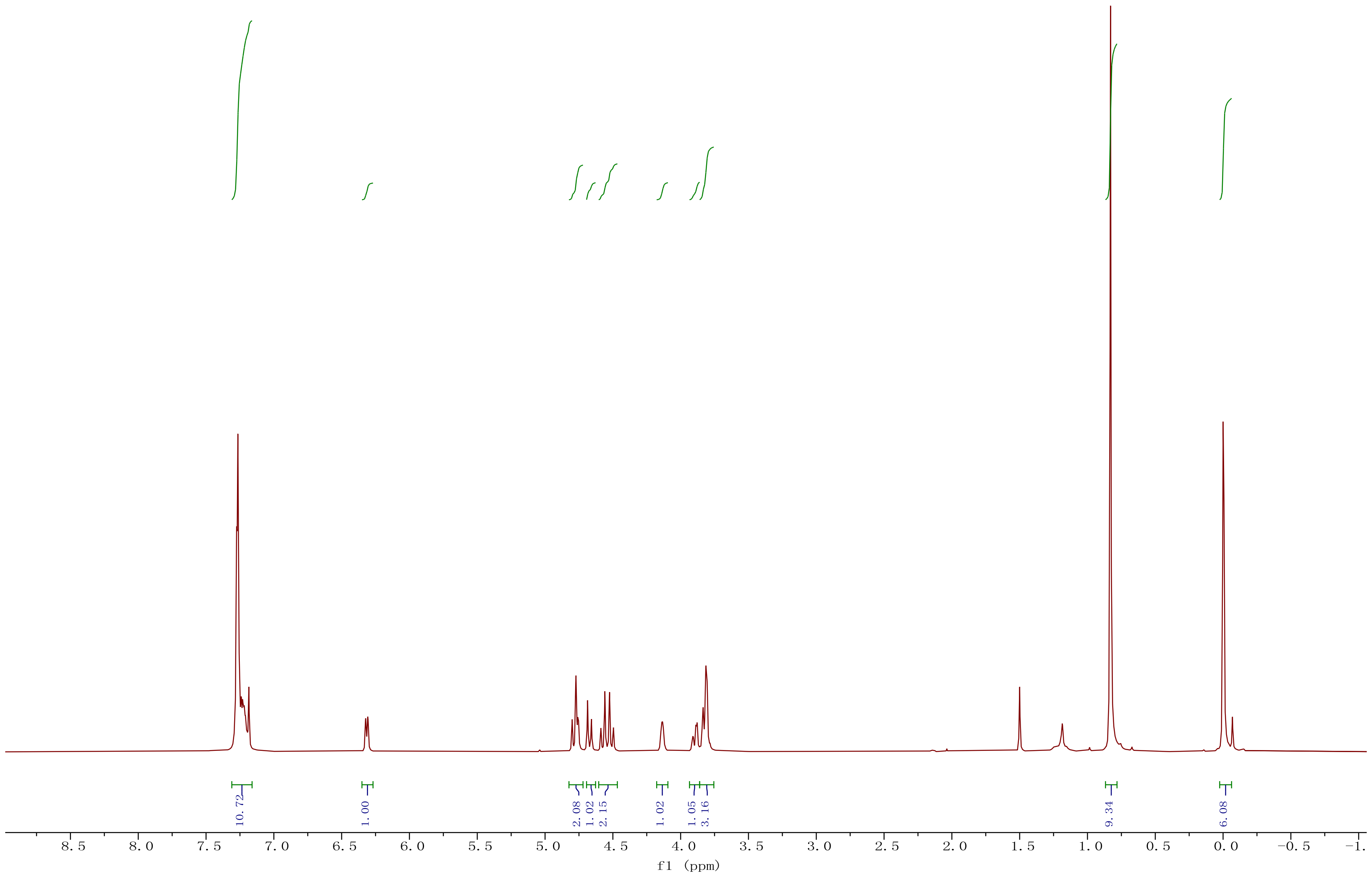

Figure S3. ^{1}H NMR spectrum (400 MHz) of **4a** in $CDCl_3$

3,4-Di-*O*-benzyl-6-*O*-acetyl-α-D-glucal 5a

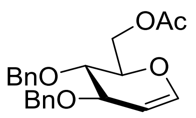

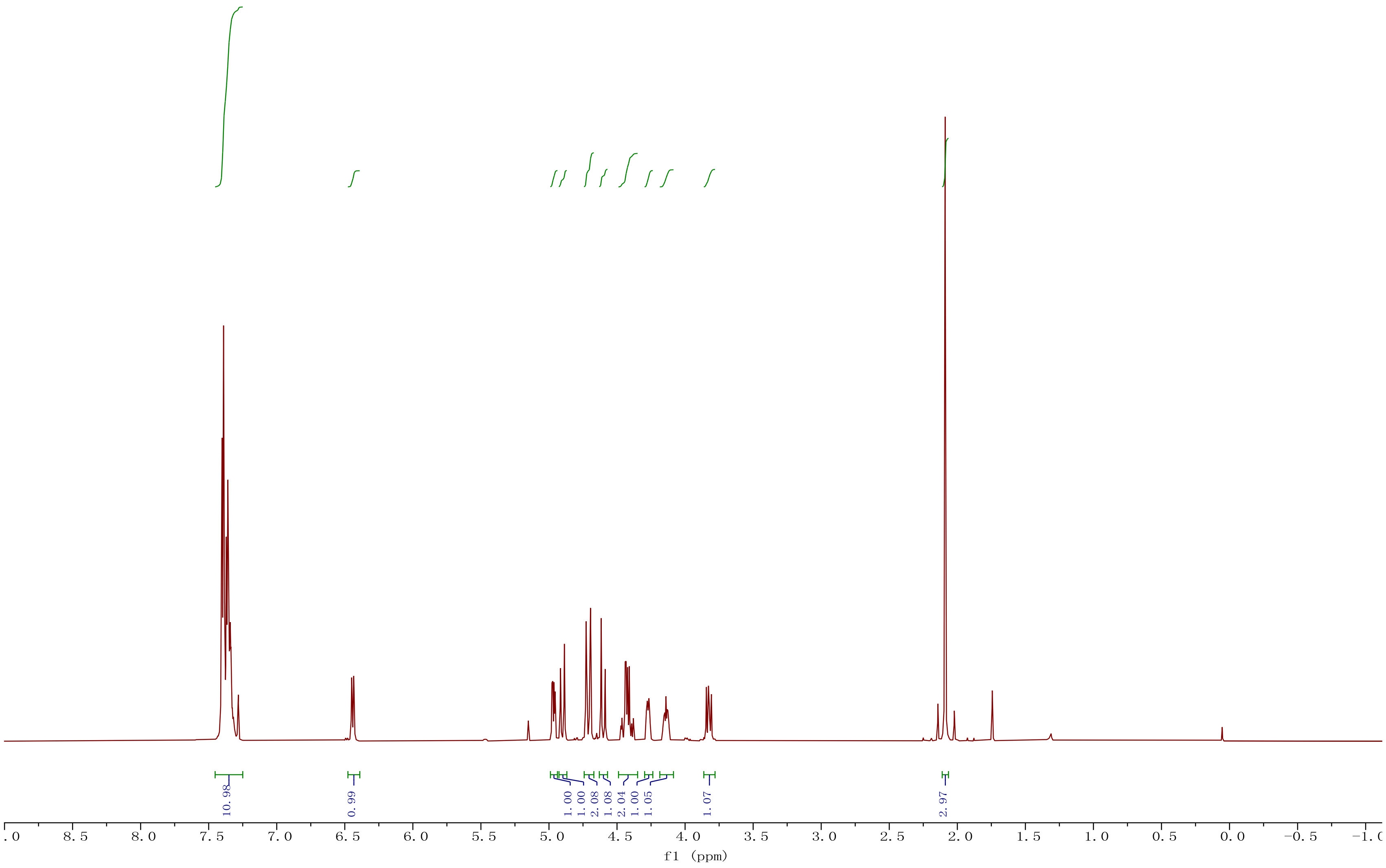

Figure S4. ^{1}H NMR spectrum (400 MHz) of **5a** in $CDCl_3$

3,4-Di-*O*-benzyl-6-*O*-benzoyl-α-D-glucal 6a

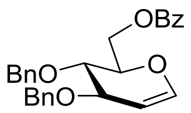

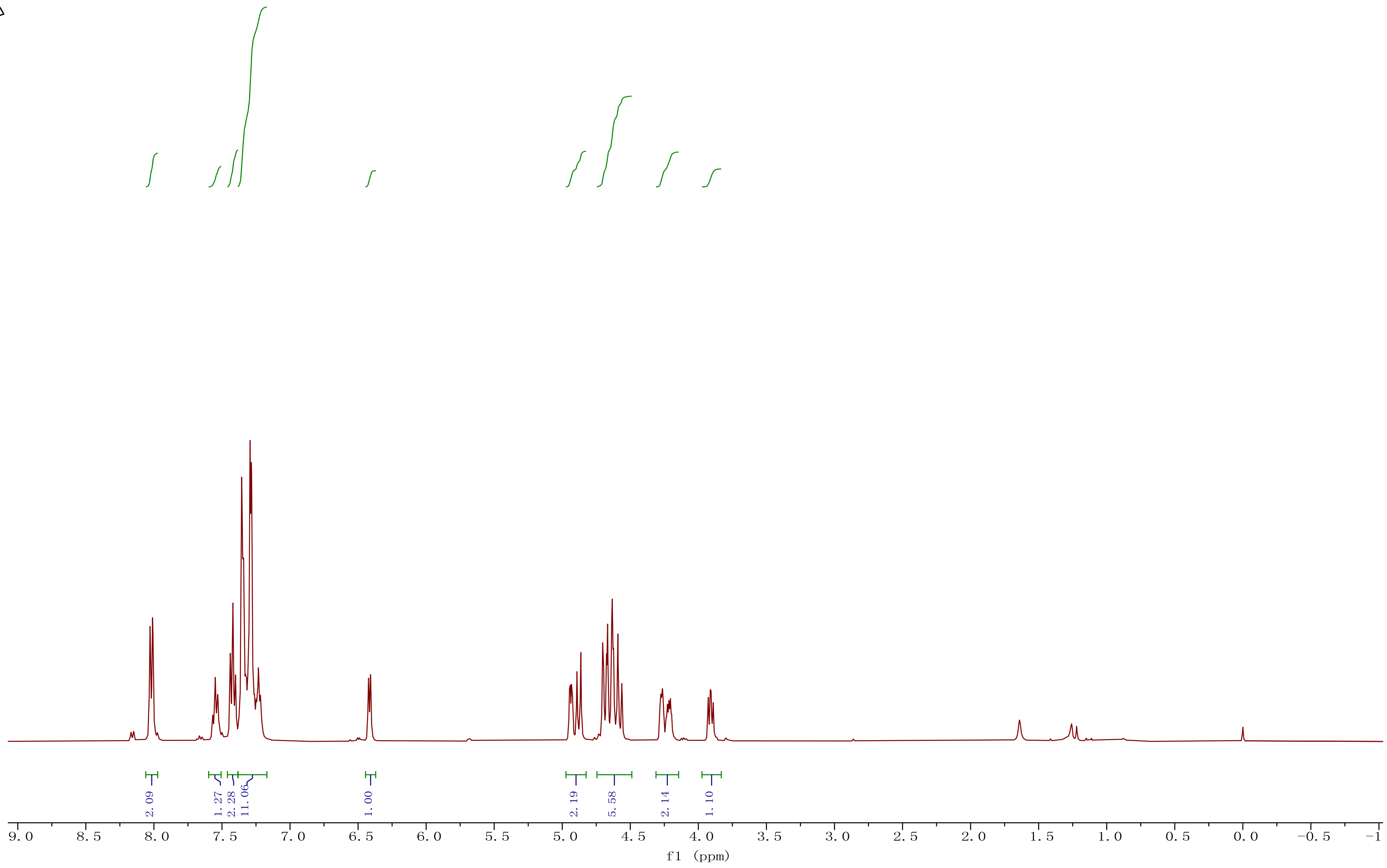

Figure S5. ^{1}H NMR spectrum (400 MHz) of **6a** in $CDCl_3$

3,4-Di-*O*-benzyl-6-*O*-benzoyl-α-D-glucal 6a

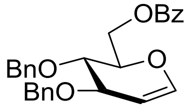

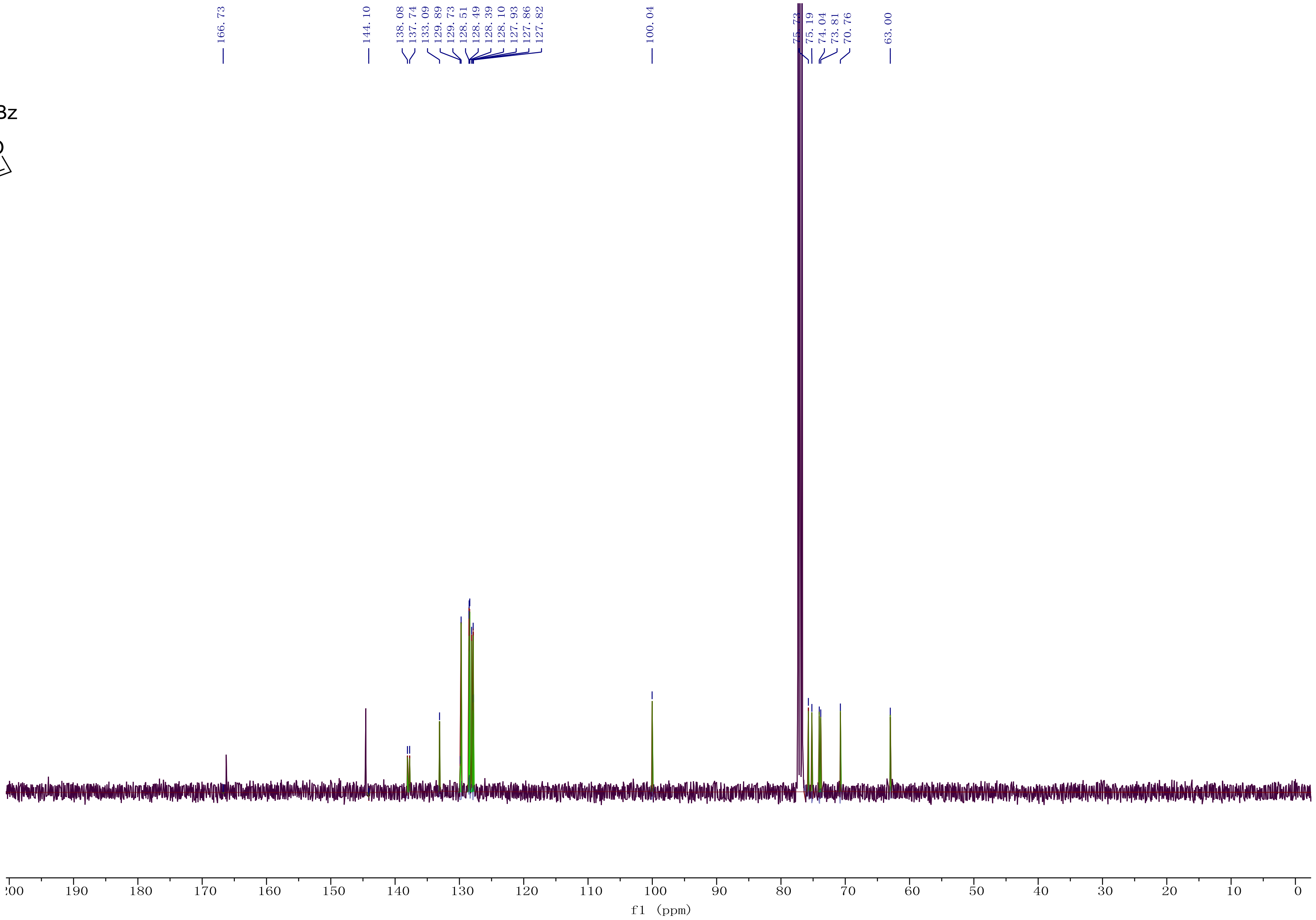

Figure S6. ^{13}C NMR spectrum (100 MHz) of **6a** in $CDCl_3$

3,4-Di-*O*-benzyl-6-*O*-benzoyl-α-D-glucal 6a

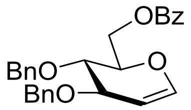

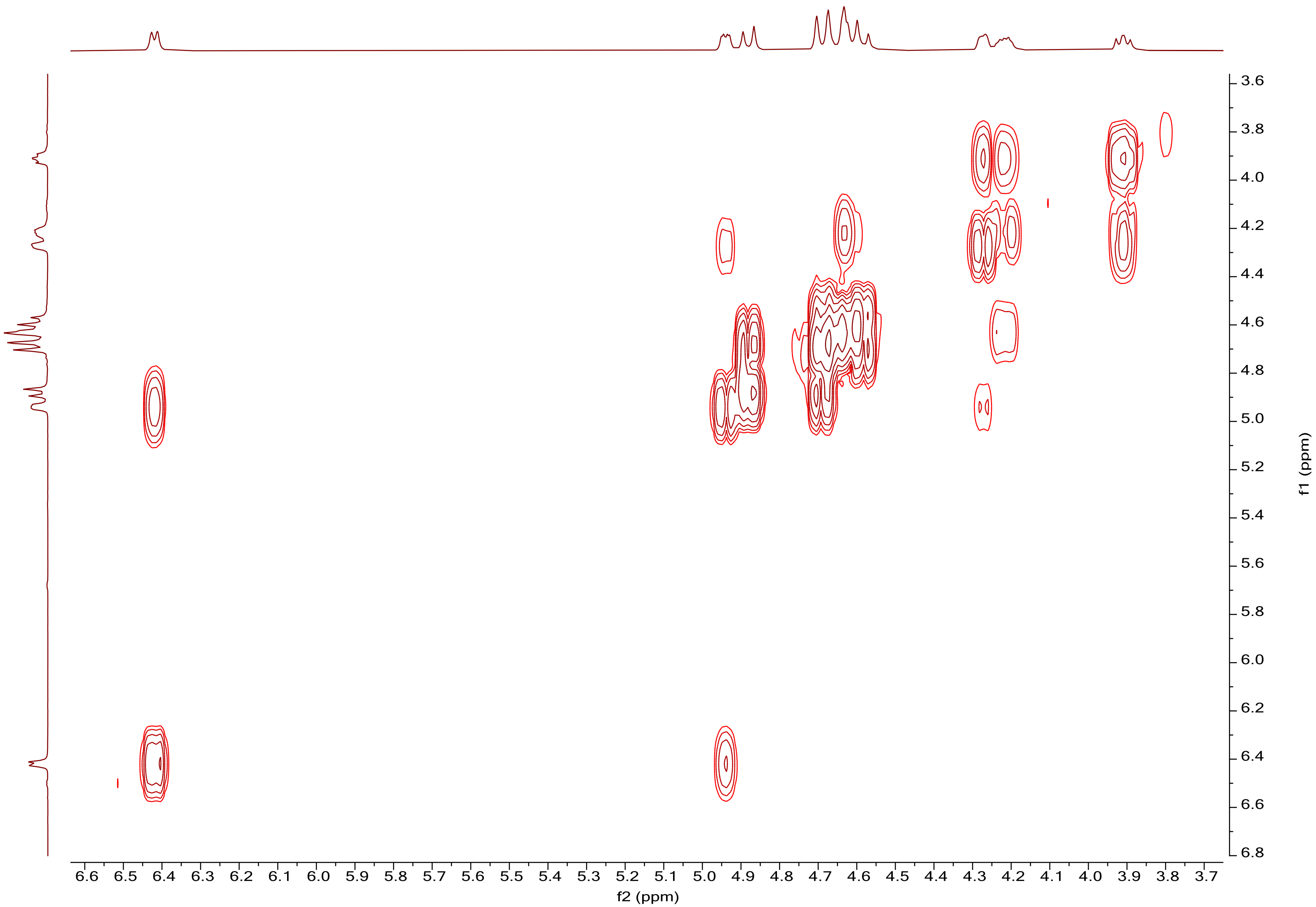

Figure S7. ¹H-¹H COSY spectrum (400 MHz) of **6a** in $CDCl_3$

3,4,6-Tri-*O*-benzoyl-α-D-glucal 7a

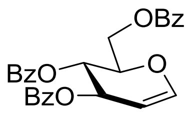

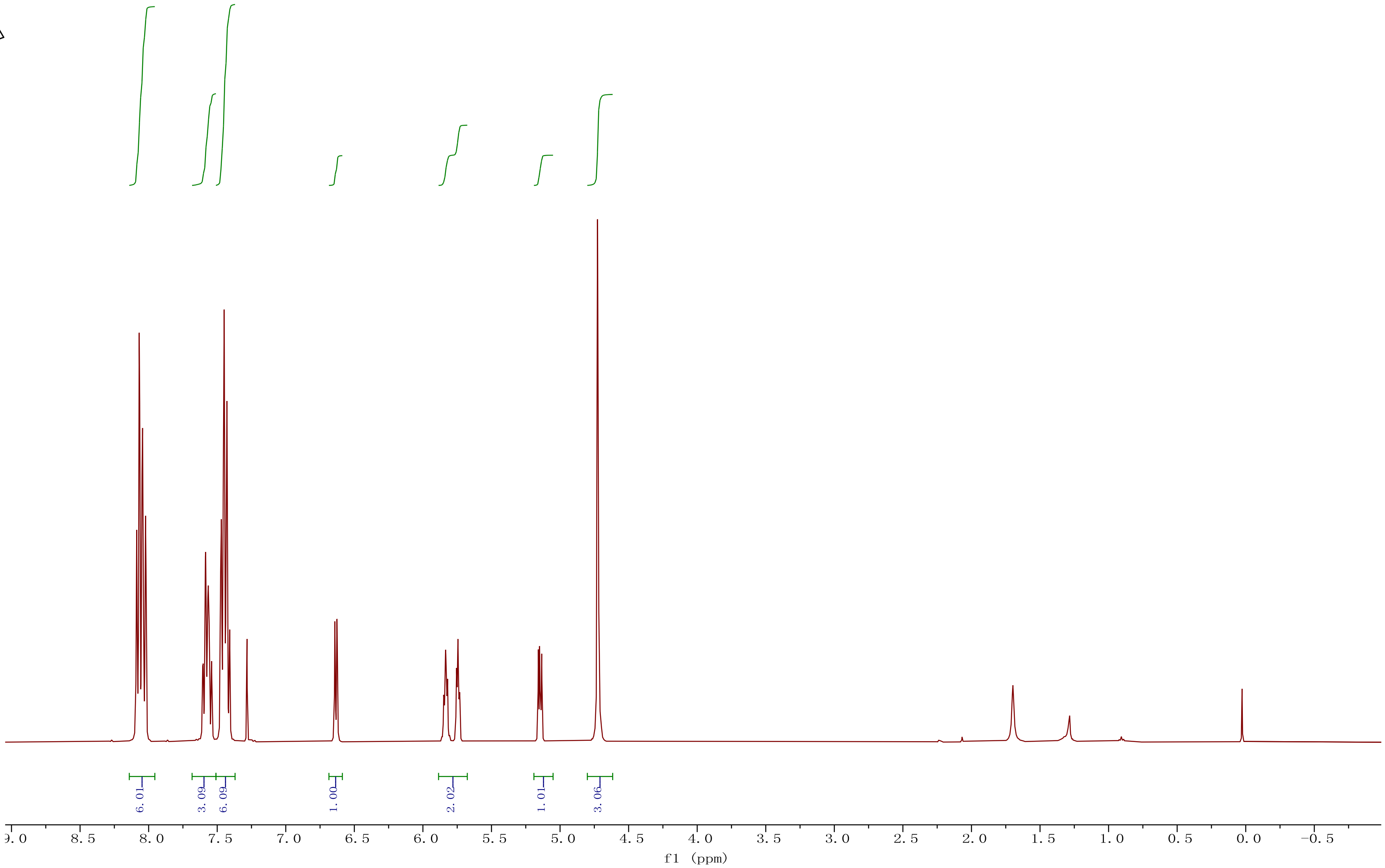

Figure S8. ^{1}H NMR spectrum (400 MHz) of **7a** in $CDCl_3$

3,4,6-Tri-*O*-acetyl-D-glucal 8a

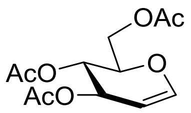

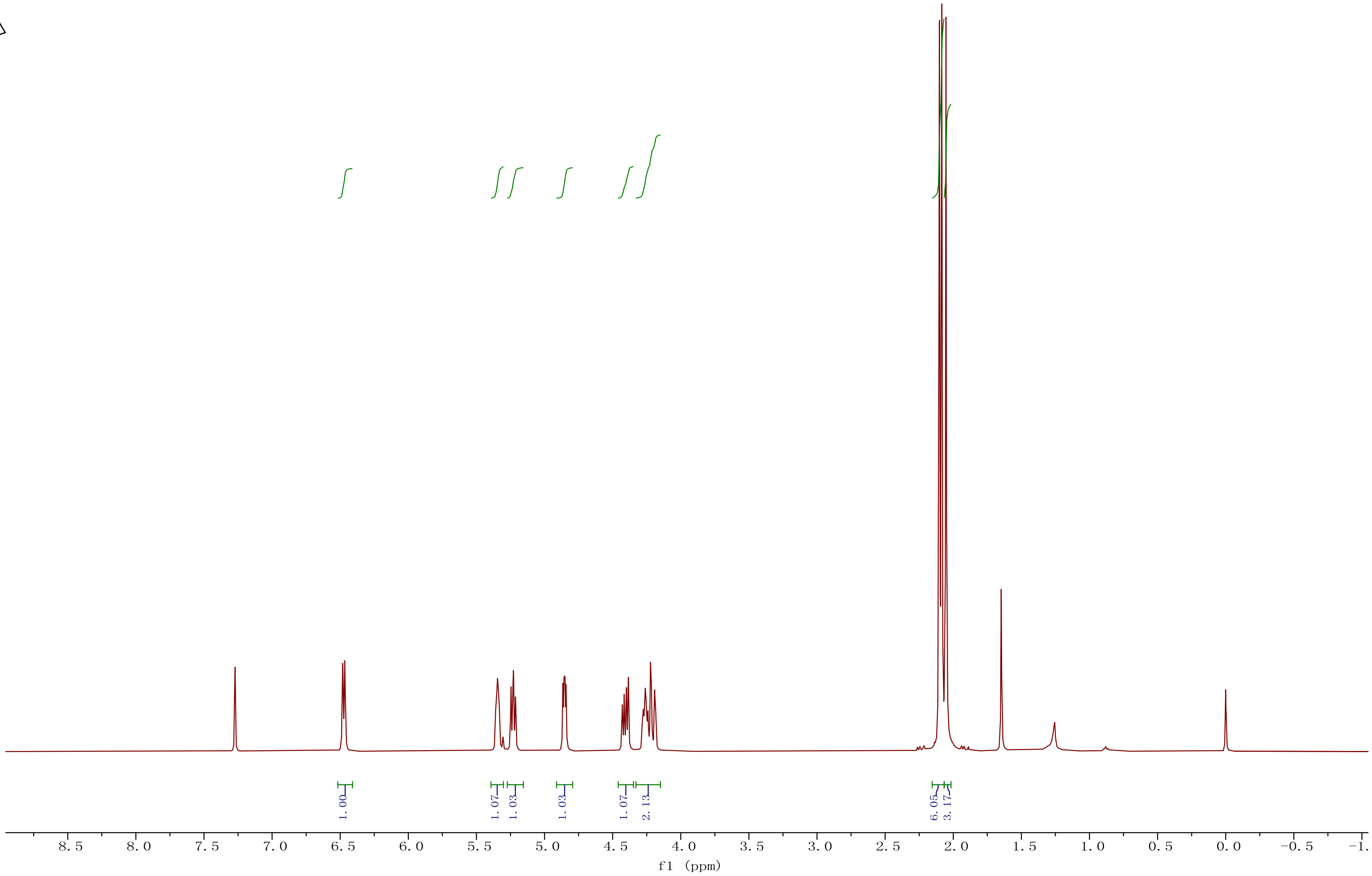

Figure S9. ^{1}H NMR spectrum (400 MHz) of **8a** in $CDCl_3$

3,4,6-Tri-*O*-ethyl-D-glucal 9a

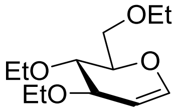

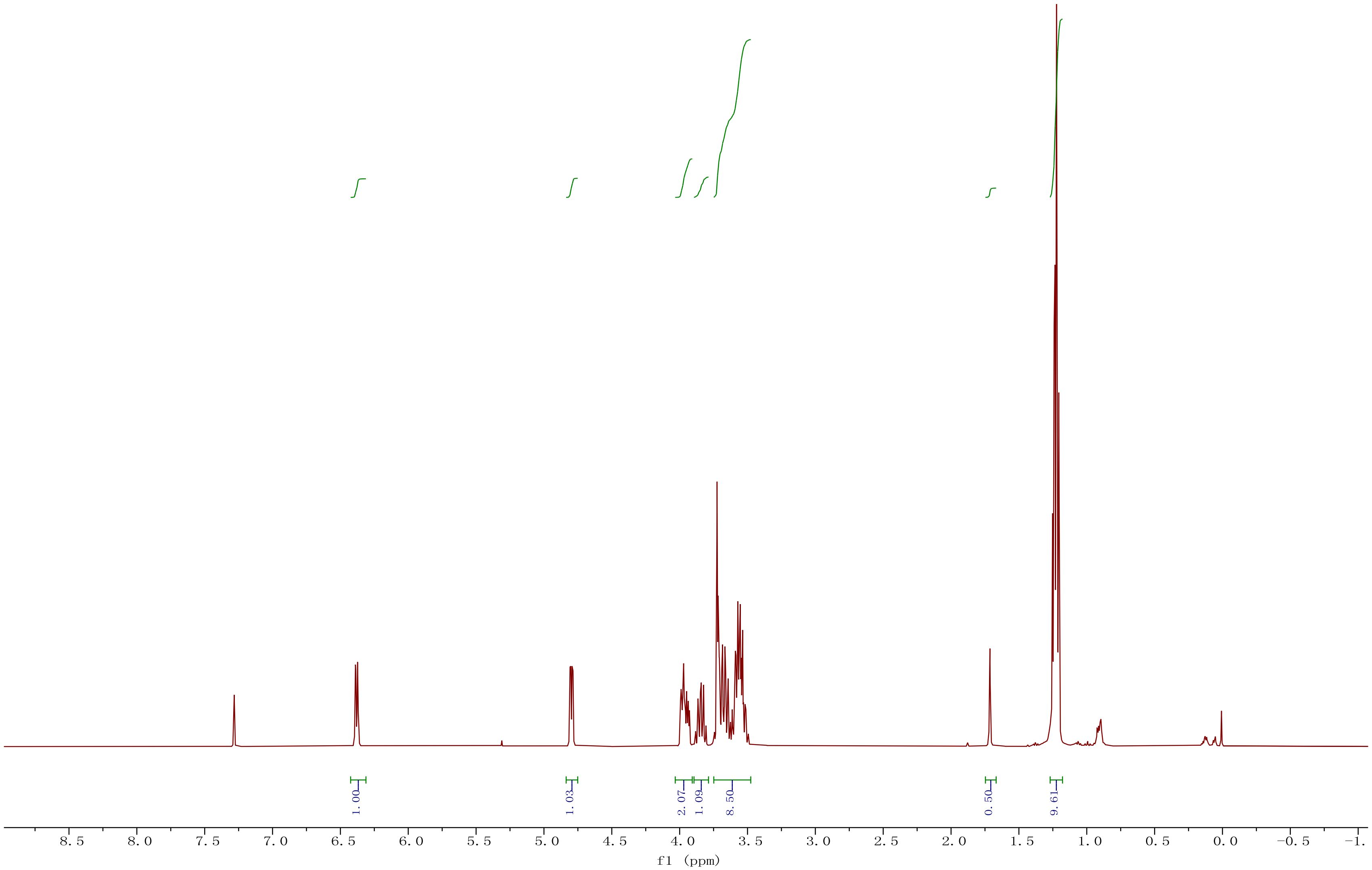

Figure S10. ^{1}H NMR spectrum (400 MHz) of **9a** in $CDCl_3$

3,4,6-Tri-*O*-tert-butyl-dimethylsily-D-glucal 10a

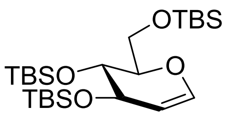

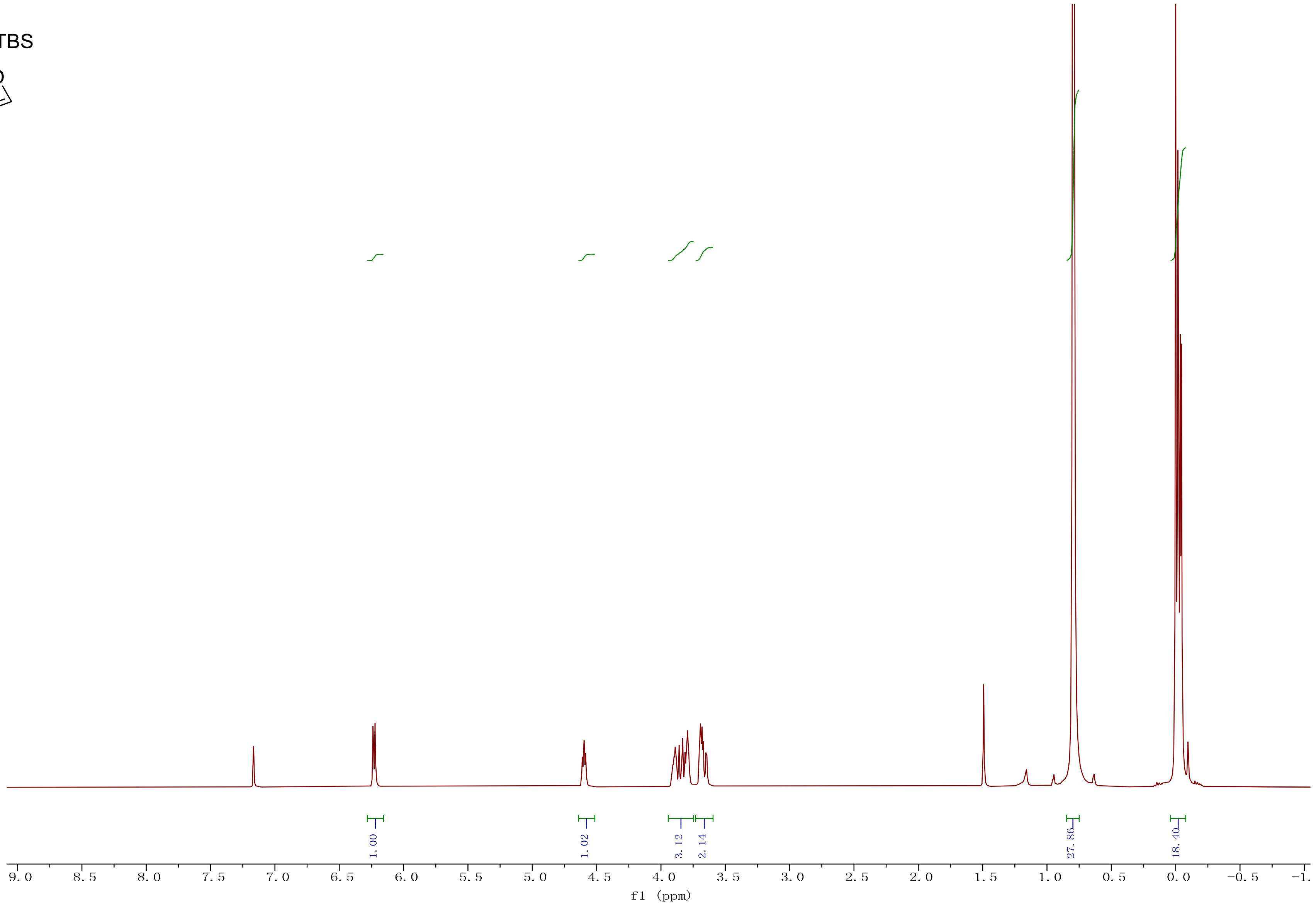

Figure S11. ^{1}H NMR spectrum (400 MHz) of **10a** in $CDCl_3$

3,4,6-Tri-*O*-*p*-methoxybenzyl-D-glucal 11a

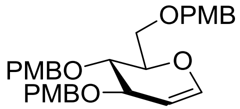

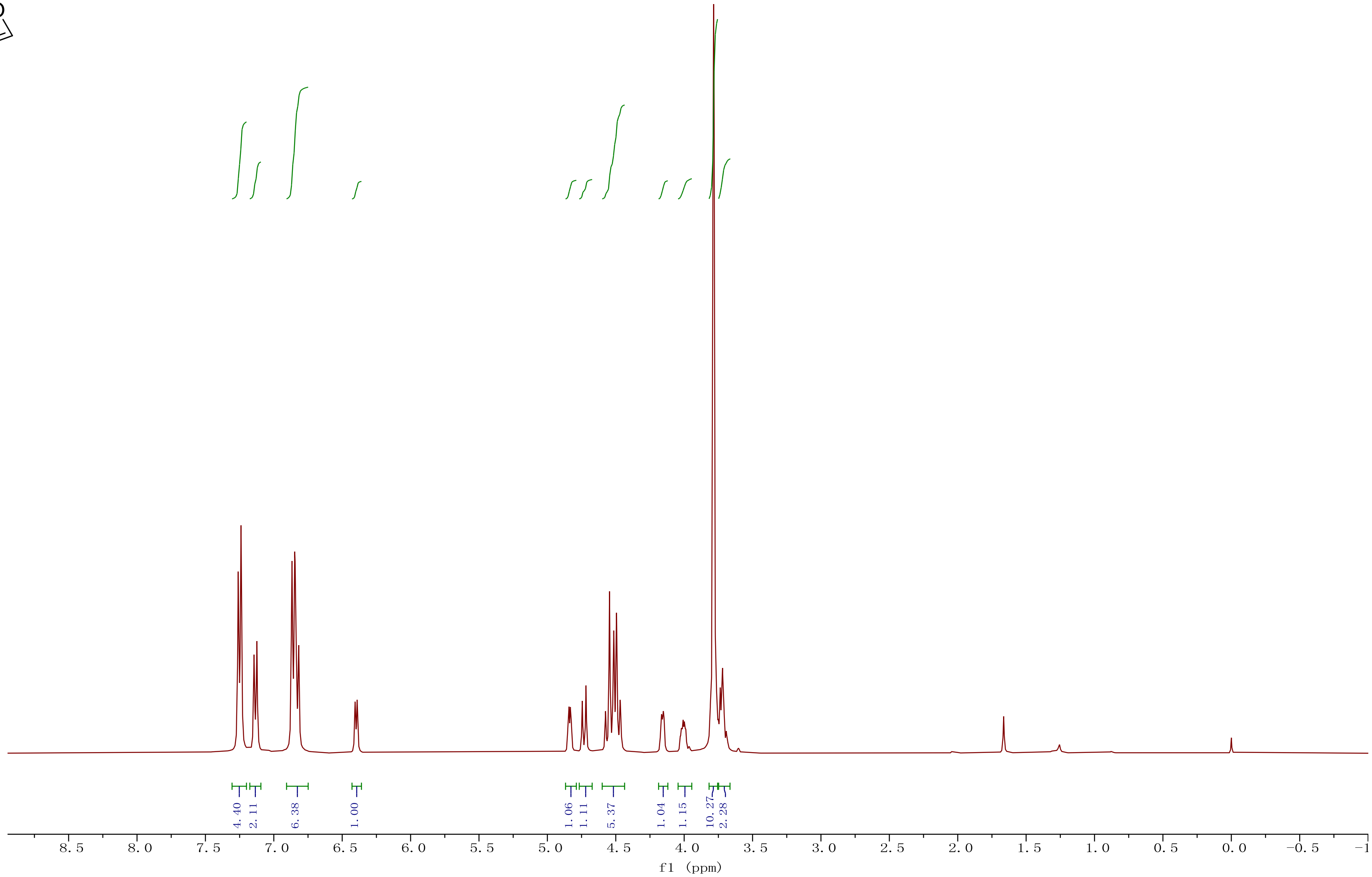

Figure S12. ^{1}H NMR spectrum (400 MHz) of **11a** in $CDCl_3$

3,4,6-Tri-*O*-benzyl-D-galactal 12a

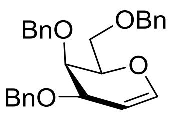

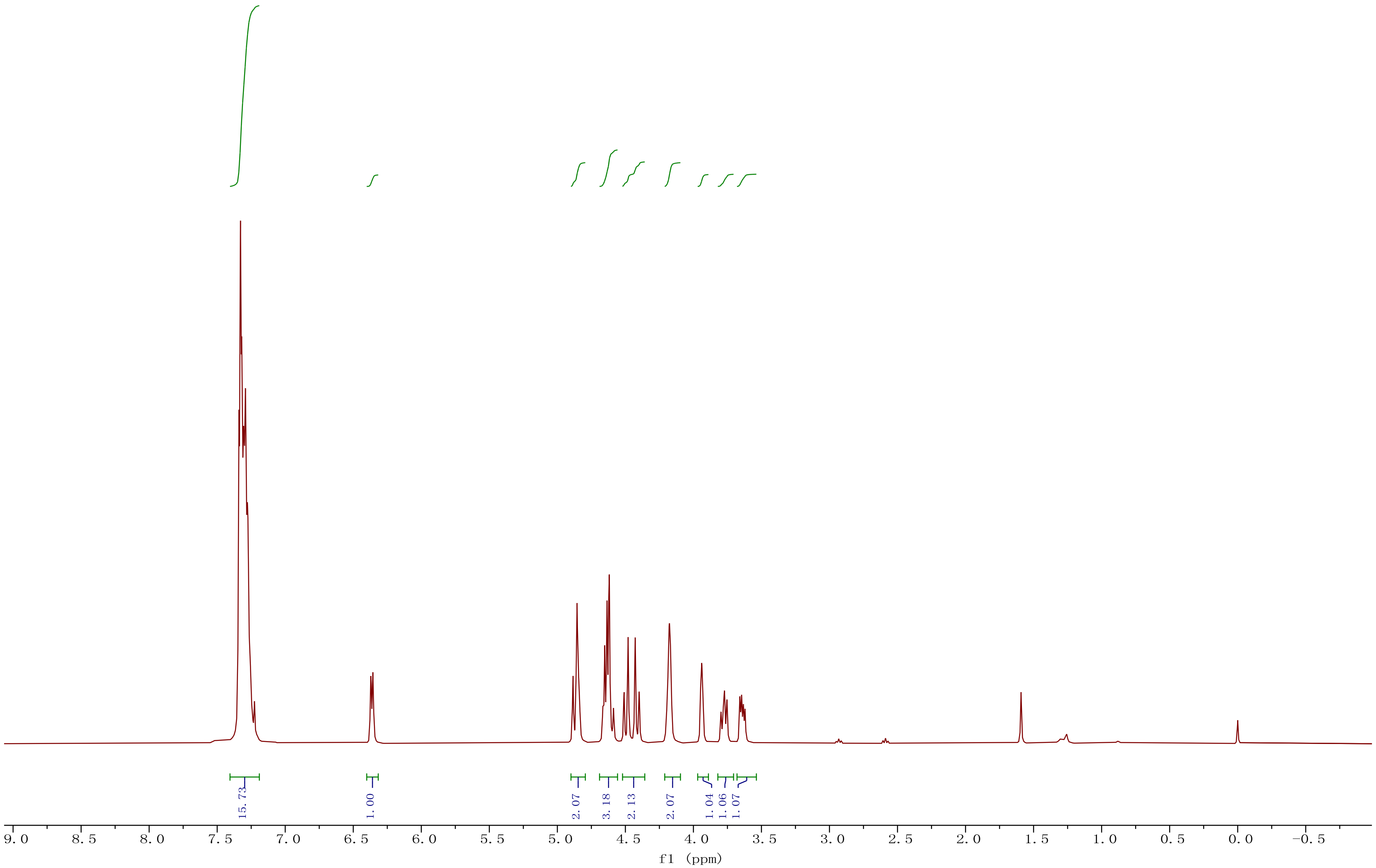

Figure S13. ^{1}H NMR spectrum (400 MHz) of **12a** in $CDCl_3$

3,4-Di-*O*-benzyl-6-*O*-tert-butyl-dimethylsily-D-galactal 13a

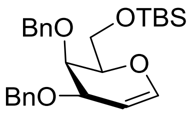

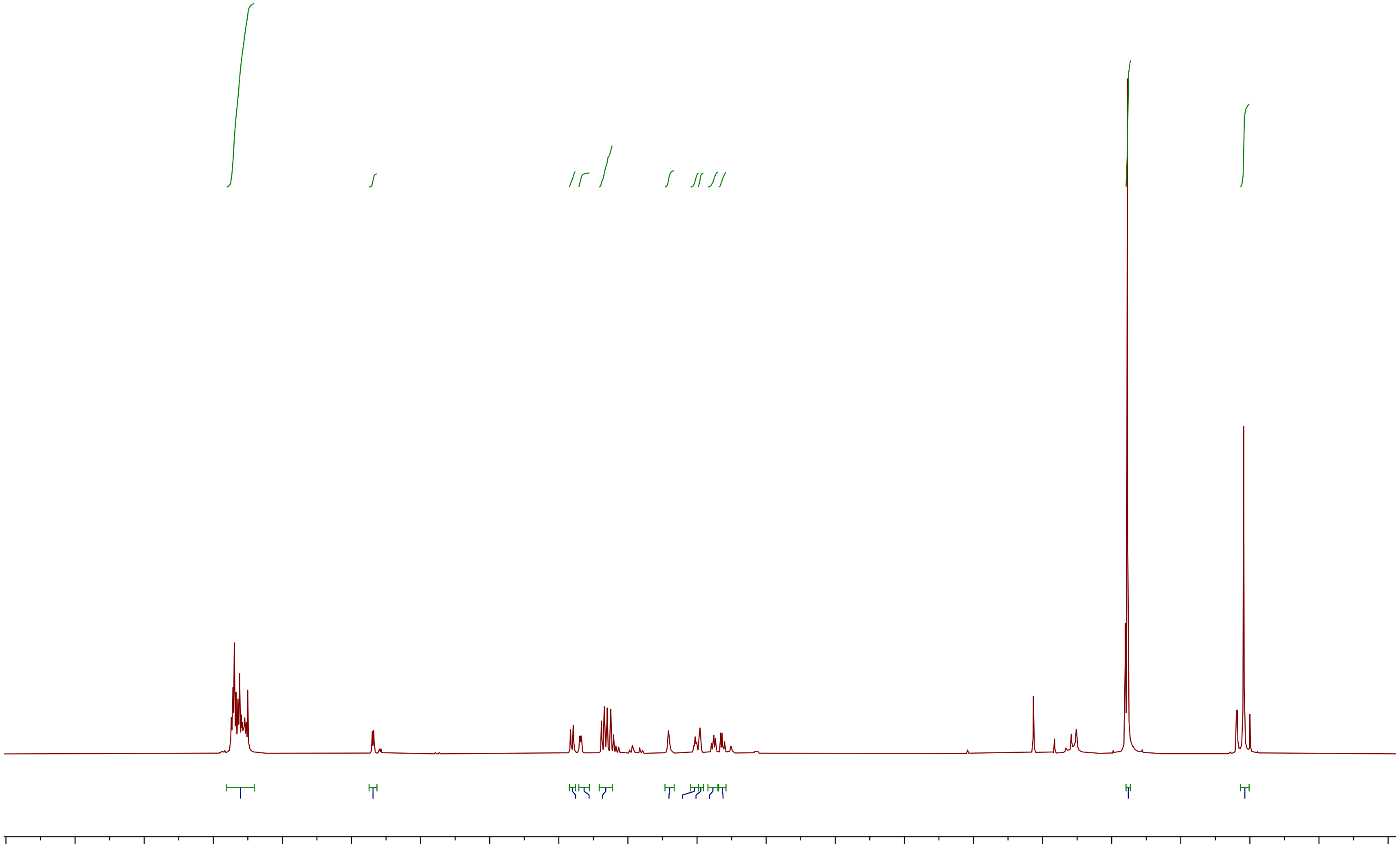

Figure S14. ^{1}H NMR spectrum (400 MHz) of **13a** in $CDCl_3$

3-*O*-Benzyl-4-*O*-acetyl-6-*O*-tert-butyl-dimethylsily-D-galactal 14a

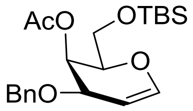

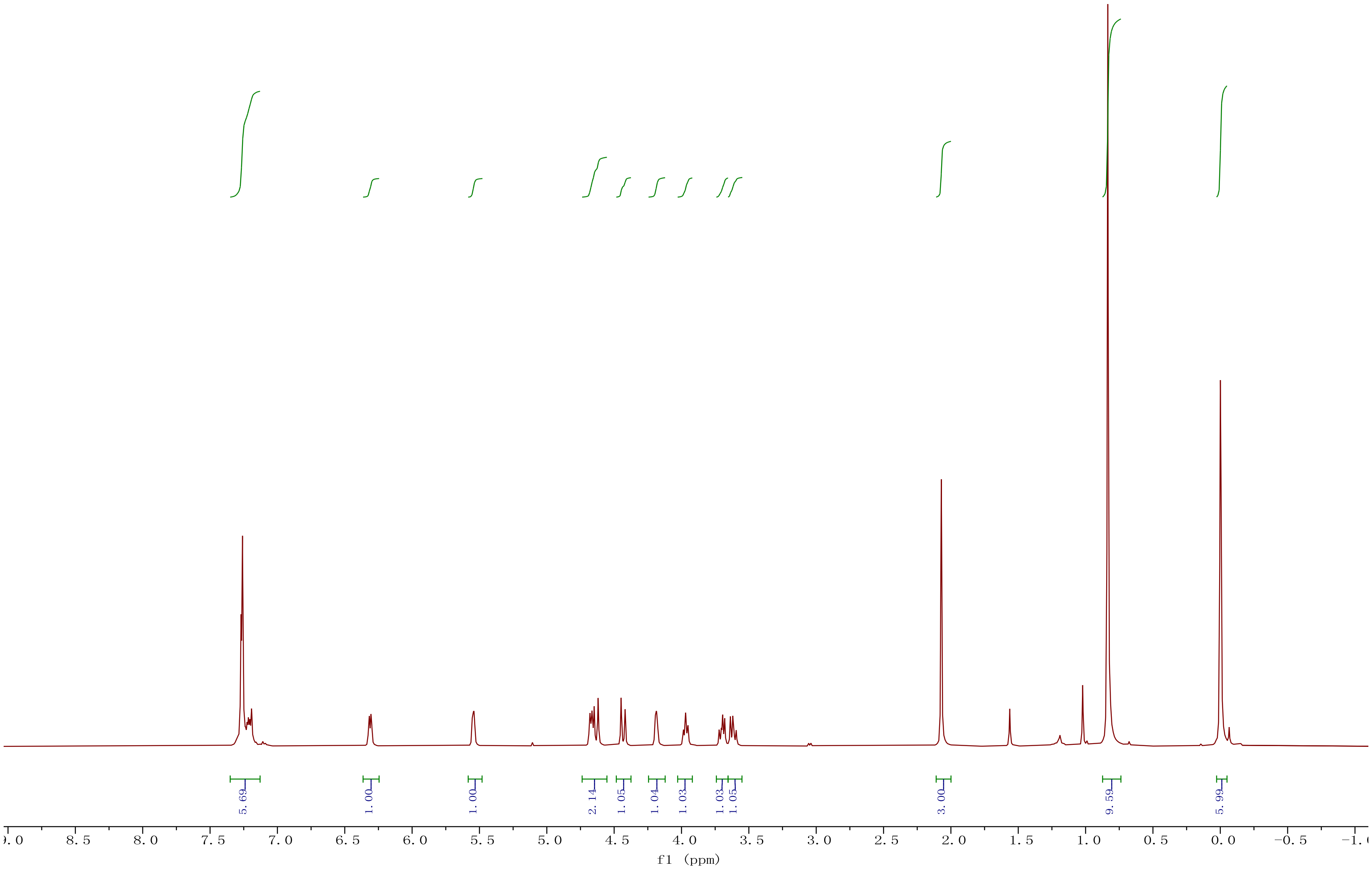

Figure S15. ^{1}H NMR spectrum (400 MHz) of **14a** in $CDCl_3$

3,4-Di-*O*-benzyl-D-xylal 15a

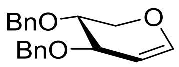

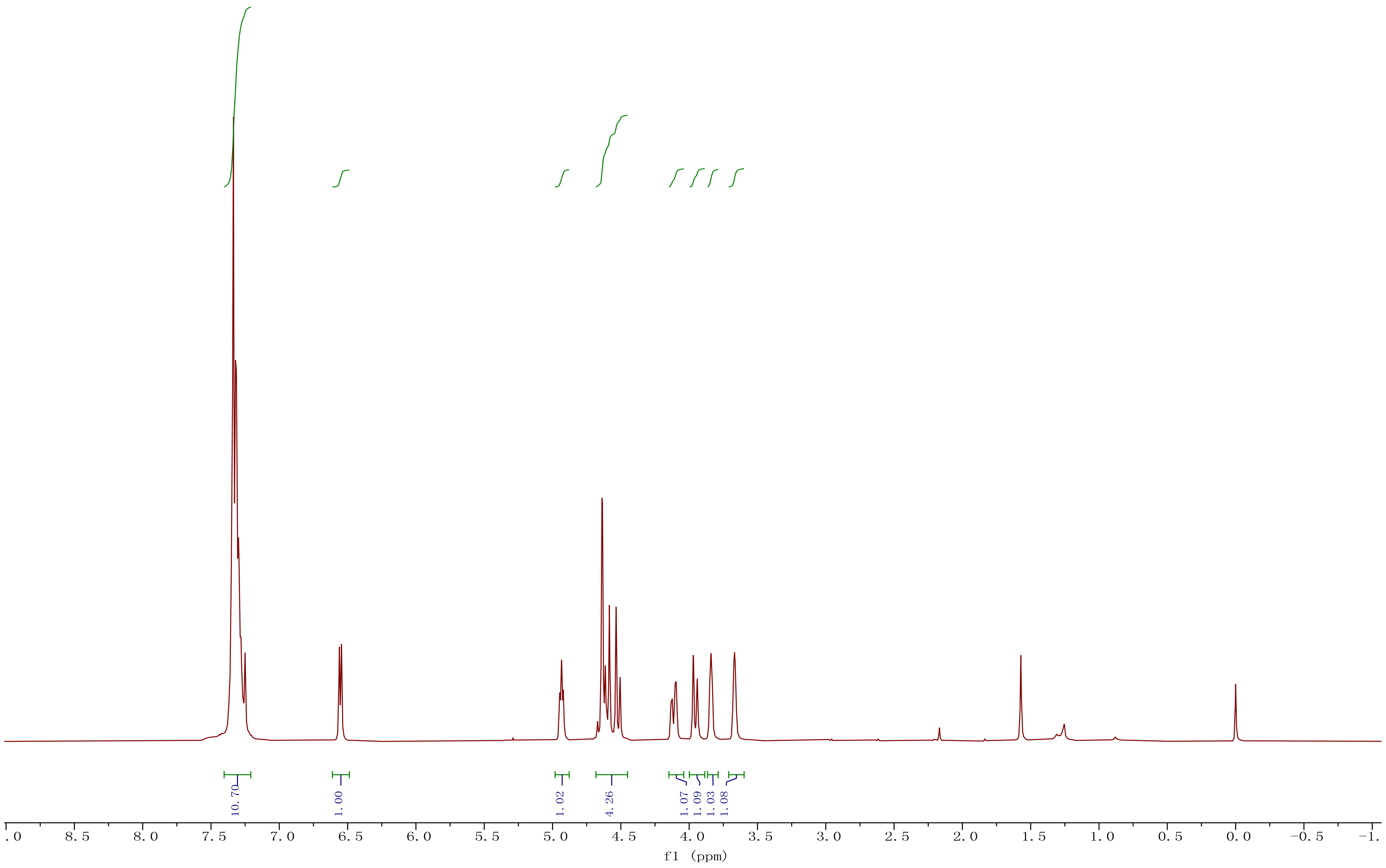

Figure S16. ^{1}H NMR spectrum (400 MHz) of **15a** in $CDCl_3$

2,3,3′,4,6,6′-Hexa-*O*-benzyl-D-lactal 16a

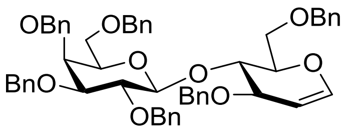

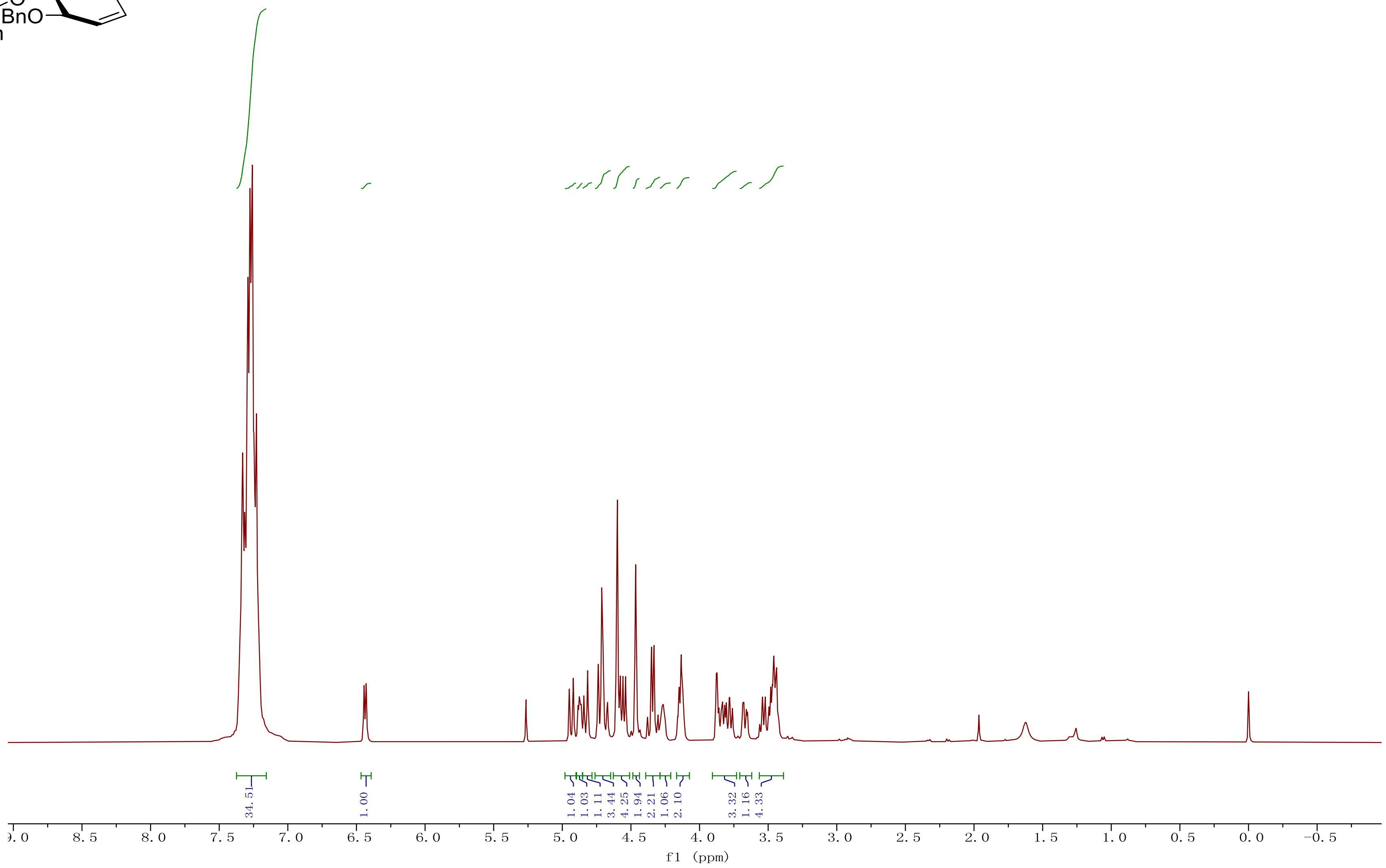

Figure S17. ^{1}H NMR spectrum (400 MHz) of **16a** in $CDCl_3$

Phenyl 3,4,6-tri-*O*-benzyl-1-thio-β-D-glucopyranoside 1

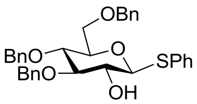

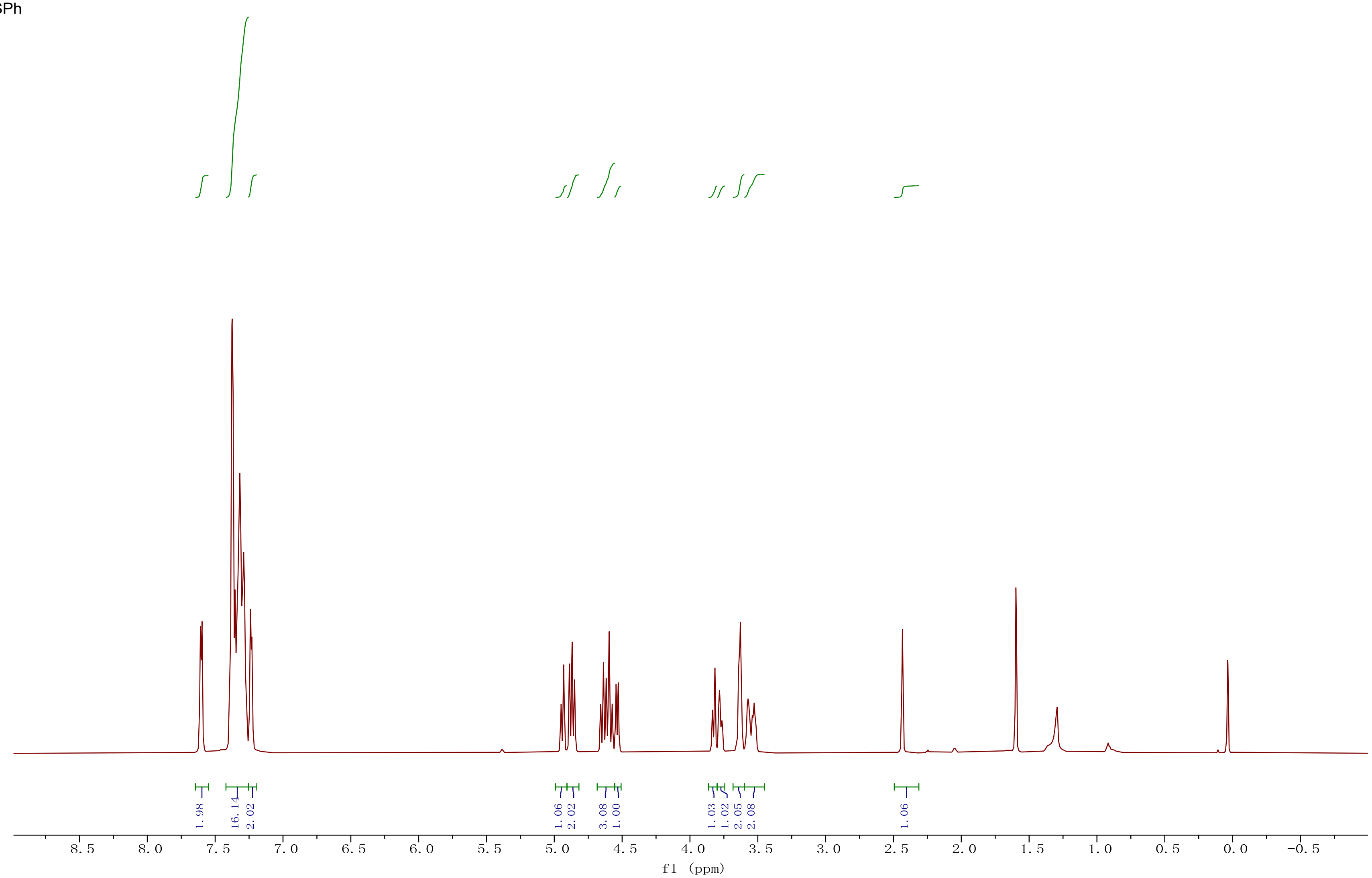

Figure S18. ^{1}H NMR spectrum (400 MHz) of **1** in $CDCl_3$

Phenyl 3,4-di-*O*-benzyl-6-*O*-tert-butyl-dimethylsily-1-thio-β-D-glucopyranoside 4

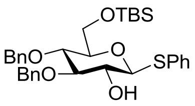

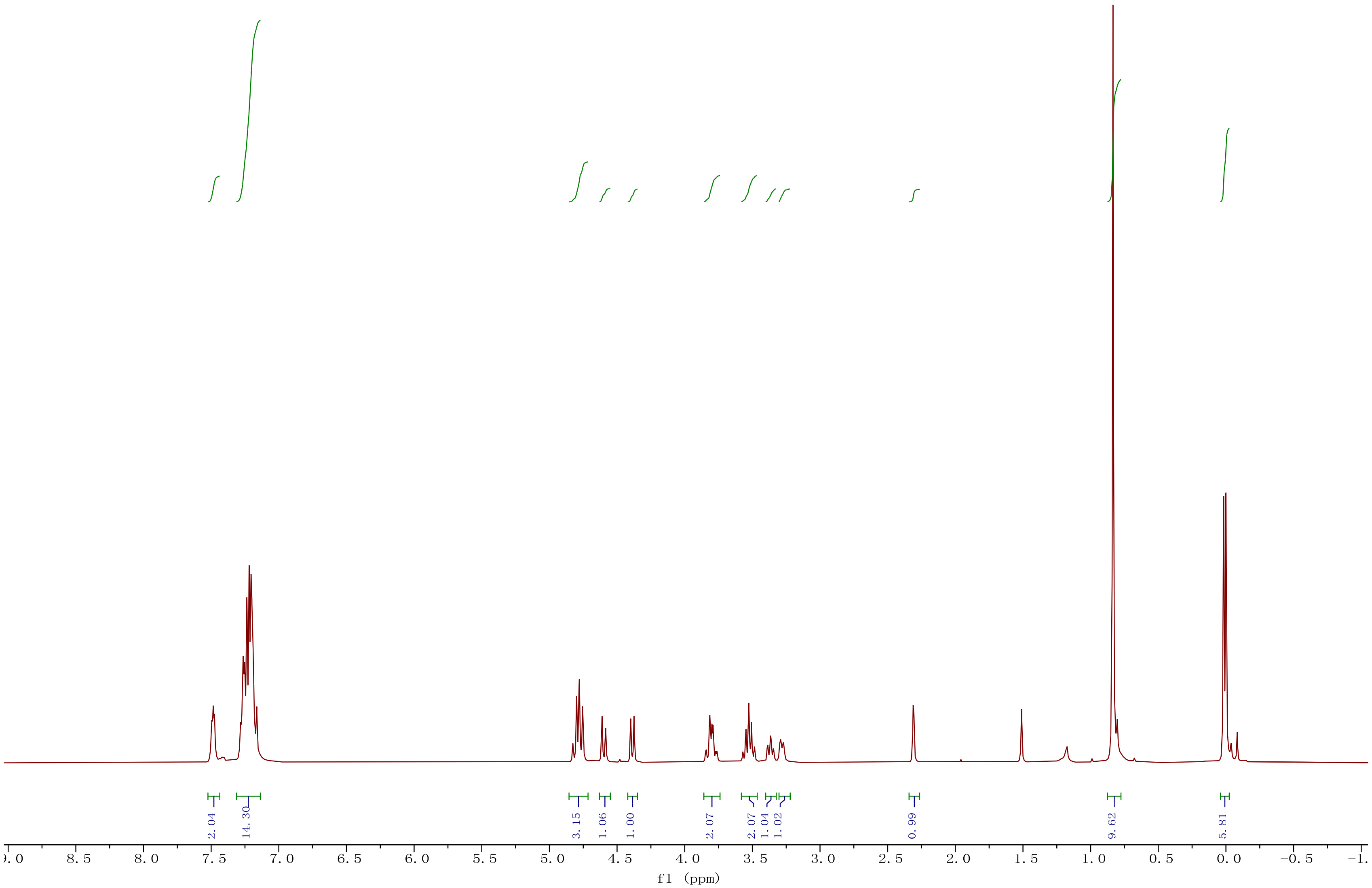

Figure S19. ¹H NMR spectrum (400 MHz) of **4** in $CDCl_3$

Phenyl 3,4-di-*O*-benzyl-6-*O*-tert-butyl-dimethylsily-1-thio-β-D-glucopyranoside 4

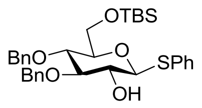

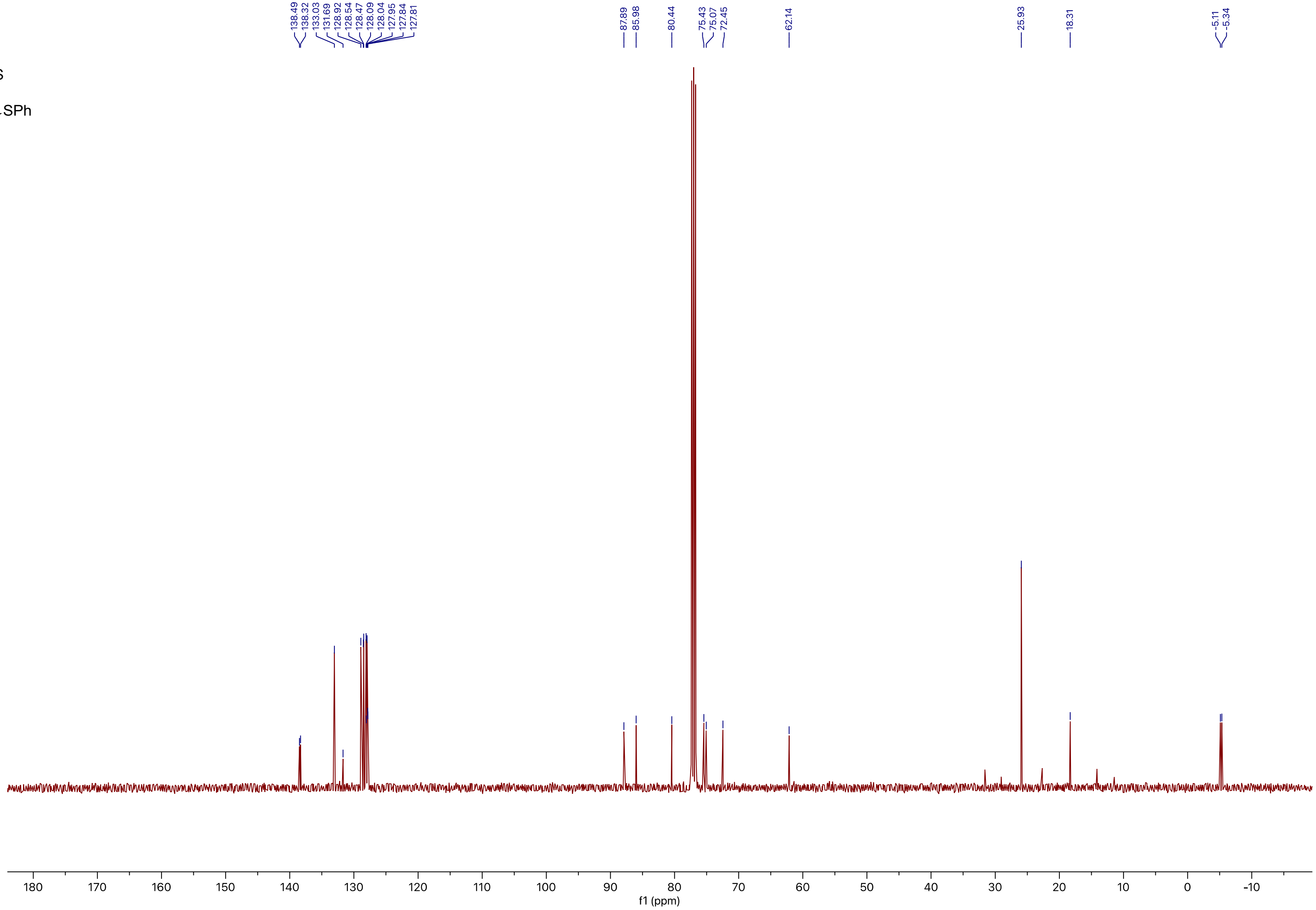

Figure S20. ^{13}C NMR spectrum (100 MHz) of **4** in $CDCl_3$

Phenyl 3,4-di-*O*-benzyl-6-*O*-acetyl-1-thio-β-D-glucopyranoside 5

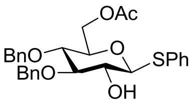

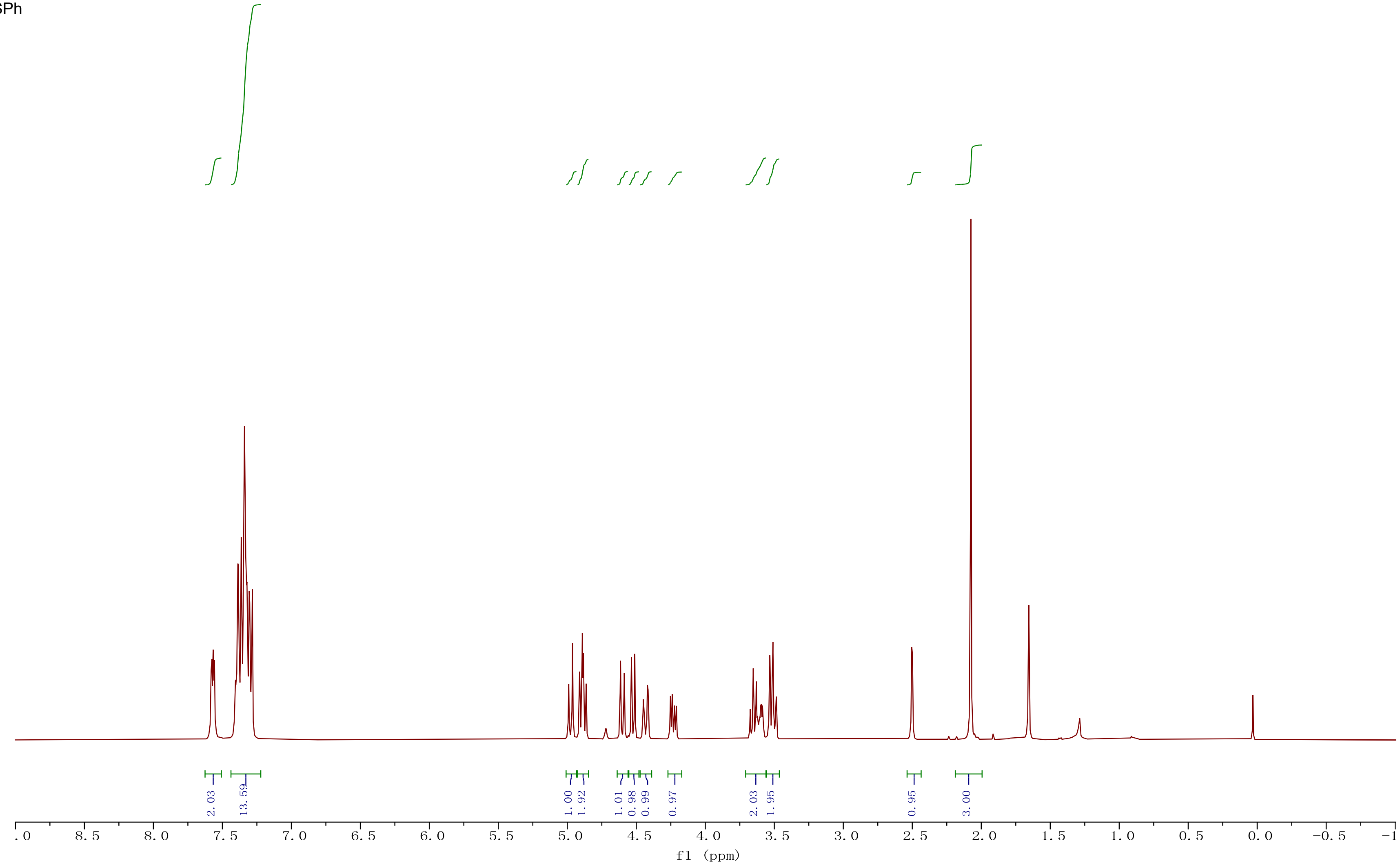

Figure S21. ¹H NMR spectrum (400 MHz) of **5** in $CDCl_3$

Phenyl 3,4-di-*O*-benzyl-6-*O*-acetyl-1-thio-β-D-glucopyranoside 5

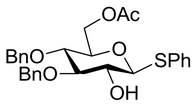

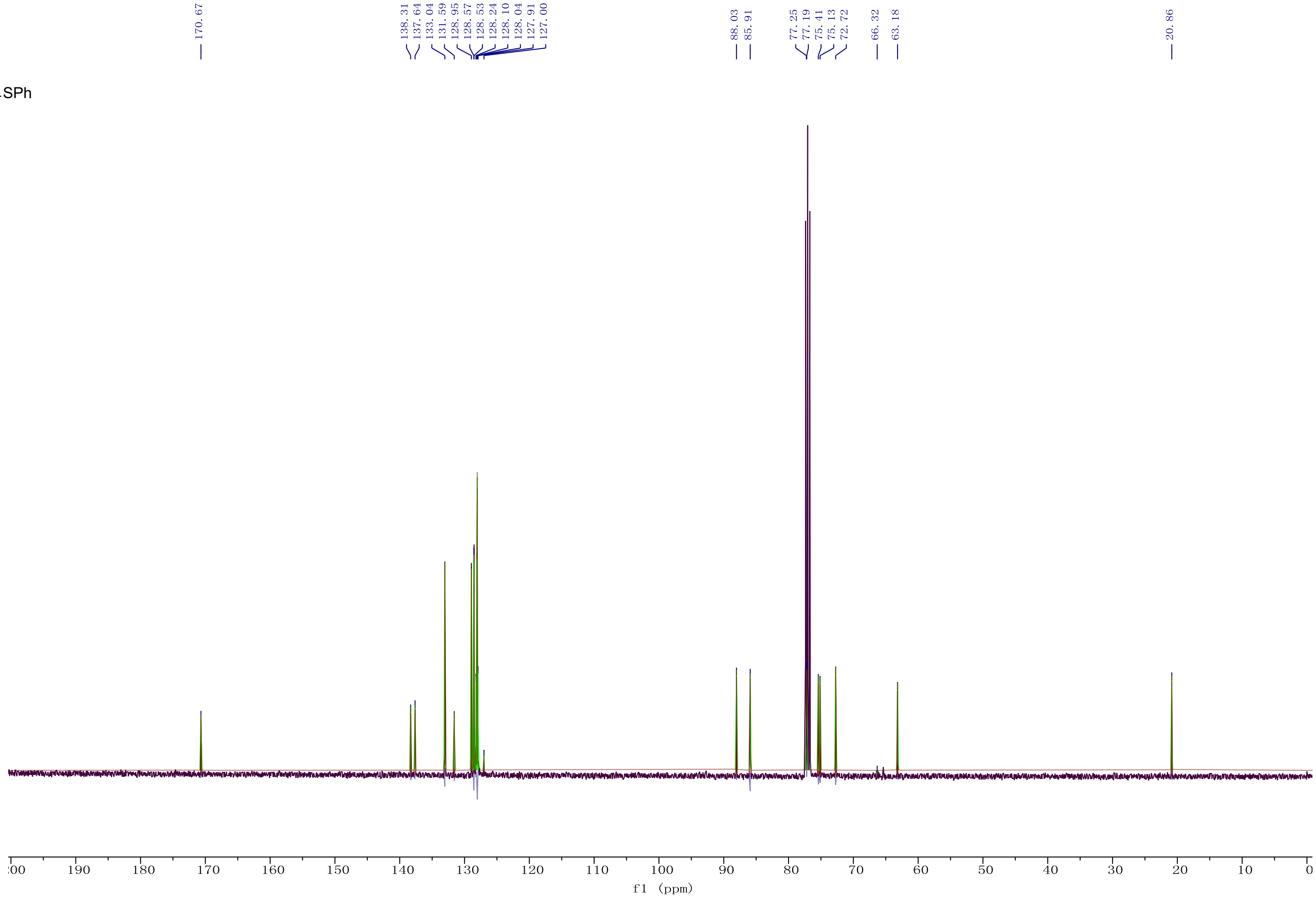

Figure S22. ^{13}C NMR spectrum (100 MHz) of **5** in $CDCl_3$

Phenyl 3,4-di-*O*-benzyl-6-*O*-benzoyl-1-thio-β-D-glucopyranoside 6

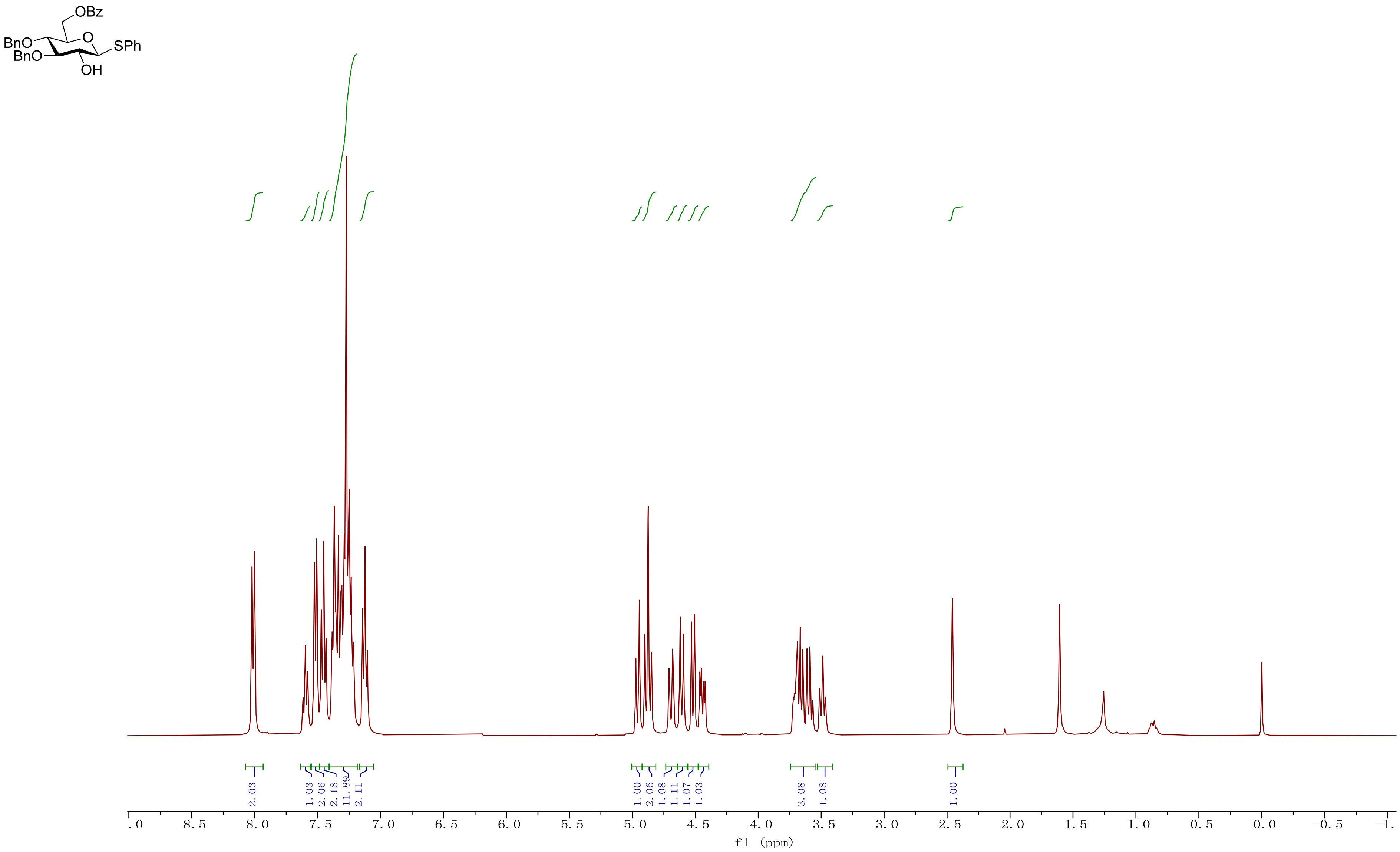

Figure S23. ^{1}H NMR spectrum (400 MHz) of **6** in $CDCl_3$

Phenyl 3,4-di-*O*-benzyl-6-*O*-benzoyl-1-thio-β-D-glucopyranoside 6

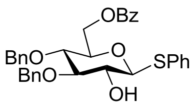

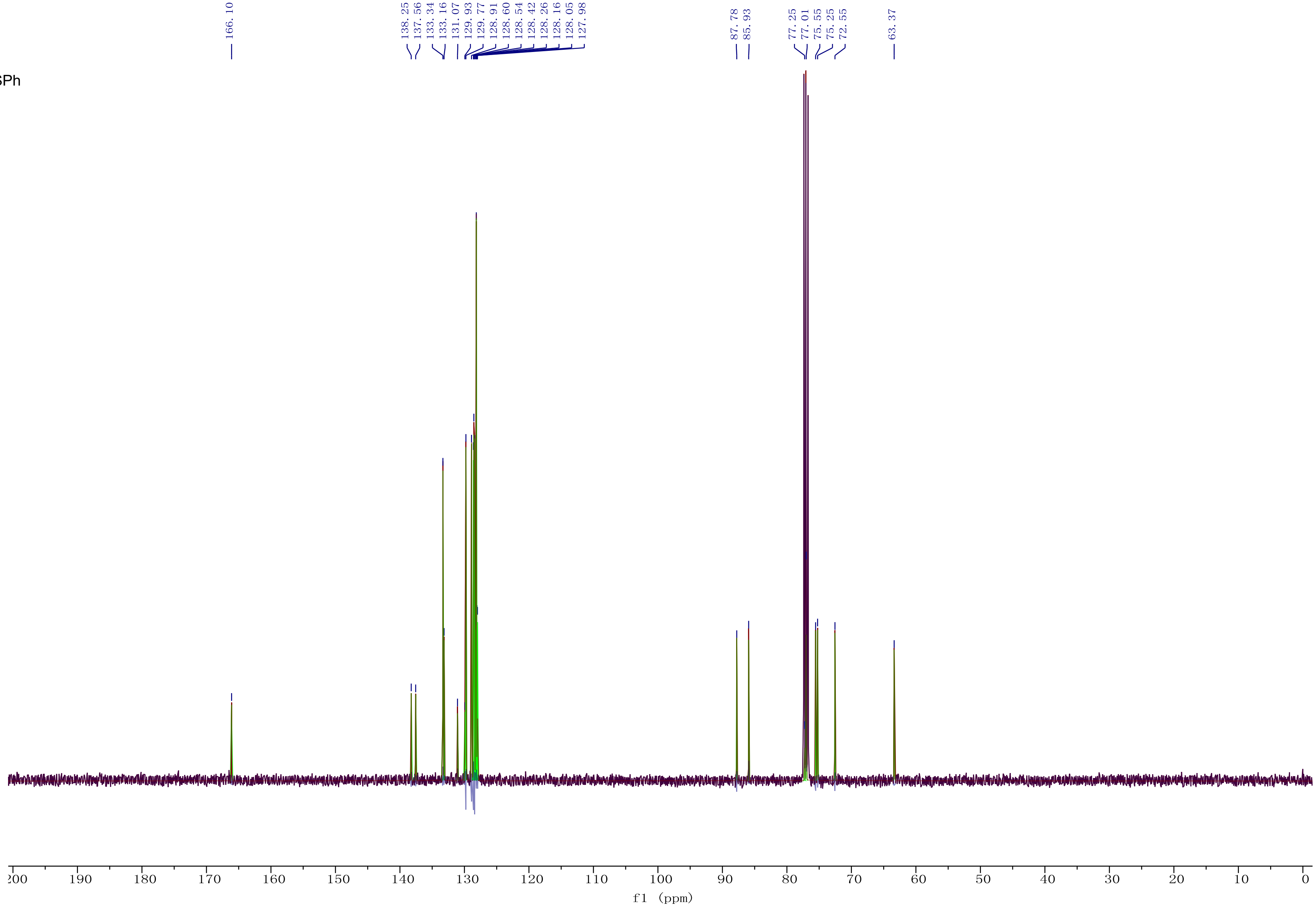

Figure S24. ^{13}C NMR spectrum (100 MHz) of **6** in $CDCl_3$

Phenyl 3,4,6-tri-*O*-benzoyl-1-thio-β-D-glucopyranoside 7

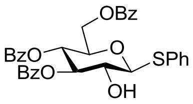

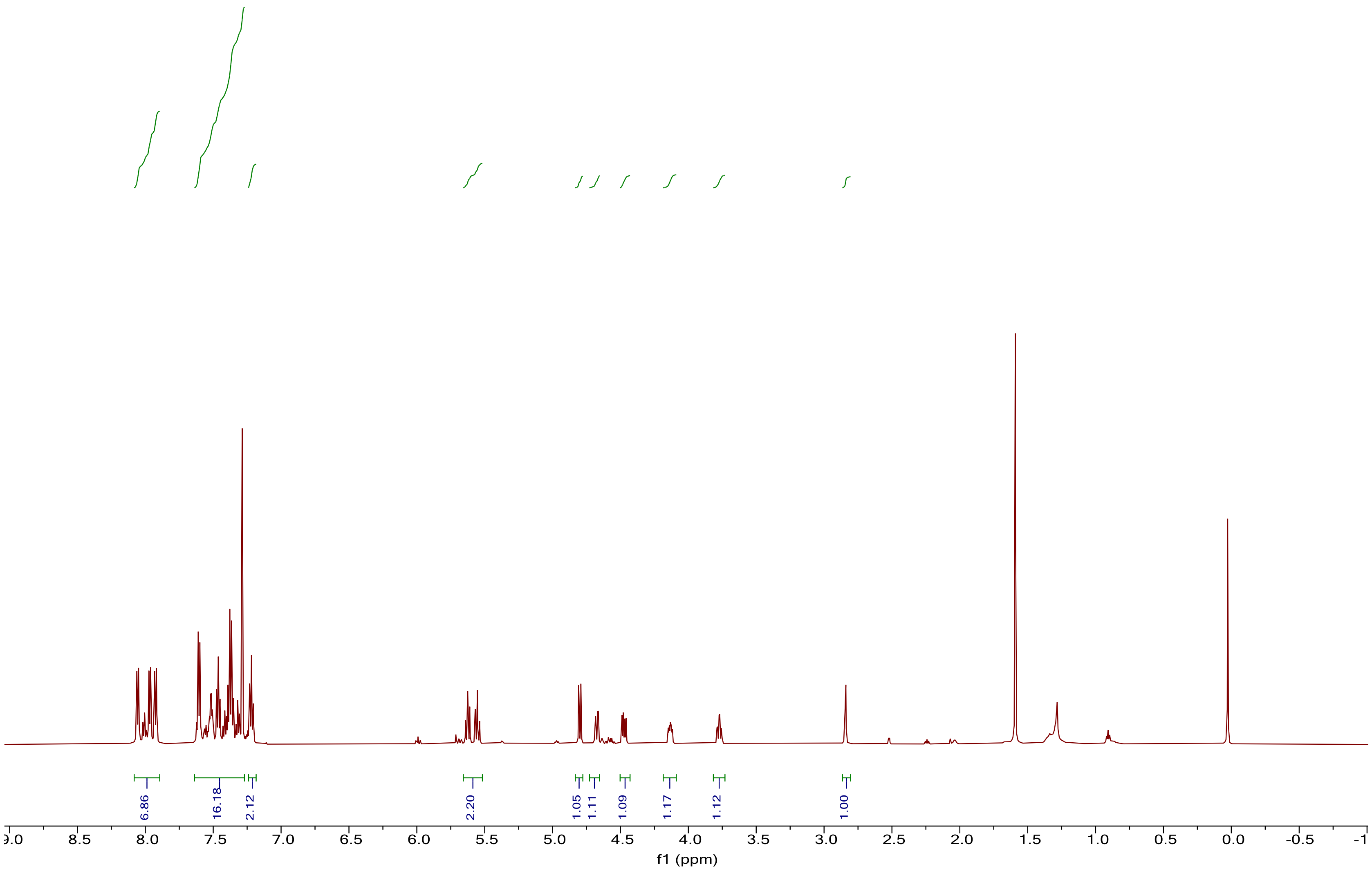

Figure S25. ^{1}H NMR spectrum (400 MHz) of **7** in $CDCl_3$

Phenyl 3,4,6-tri-*O*-benzoyl-1-thio-β-D-glucopyranoside 7

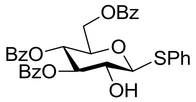

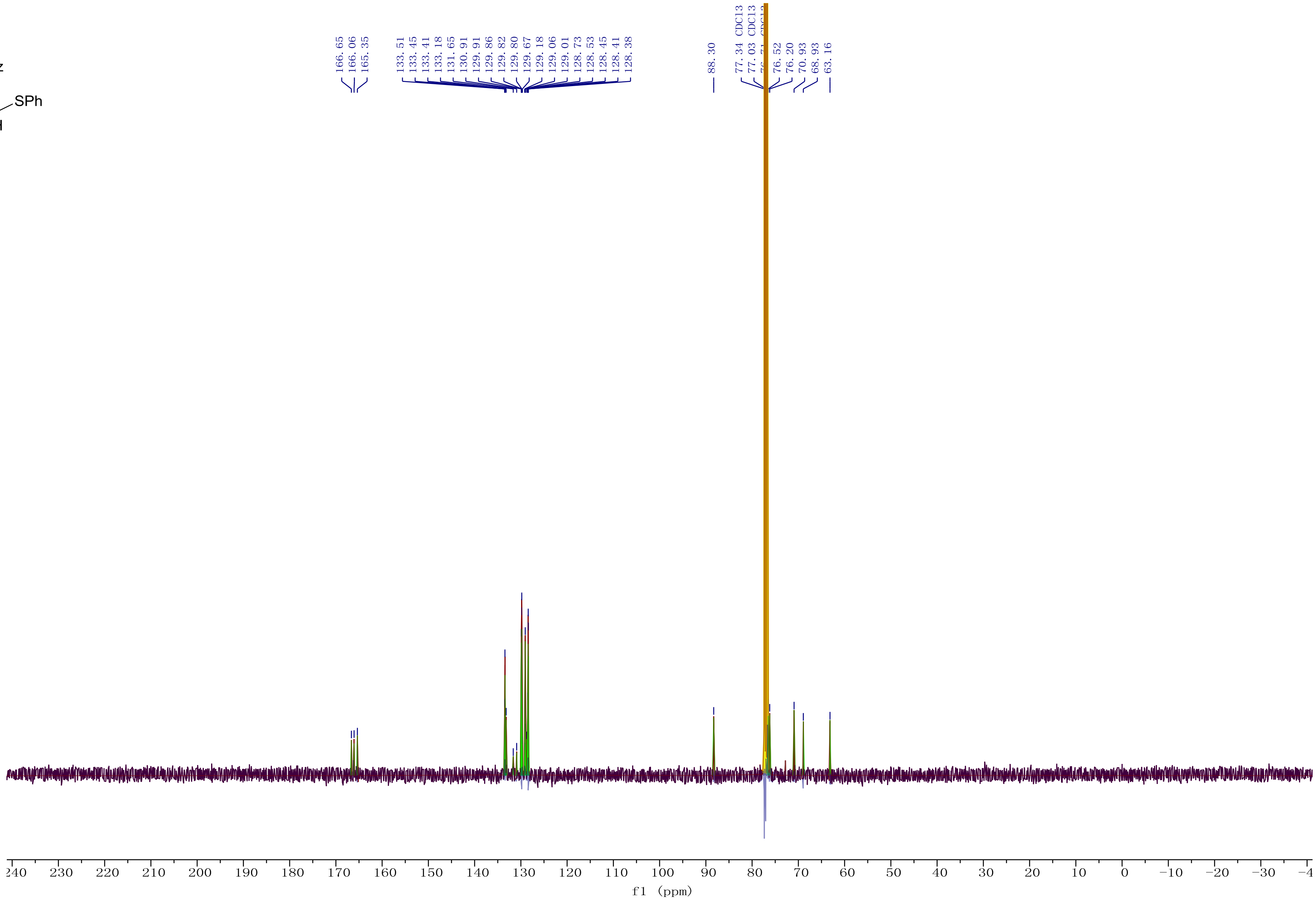

Figure S26. ^{13}C NMR spectrum (100 MHz) of **7** in $CDCl_3$

Phenyl 3,4,6-tri-*O*-acetyl-1-thio-β-D-glucopyranoside 8

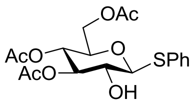

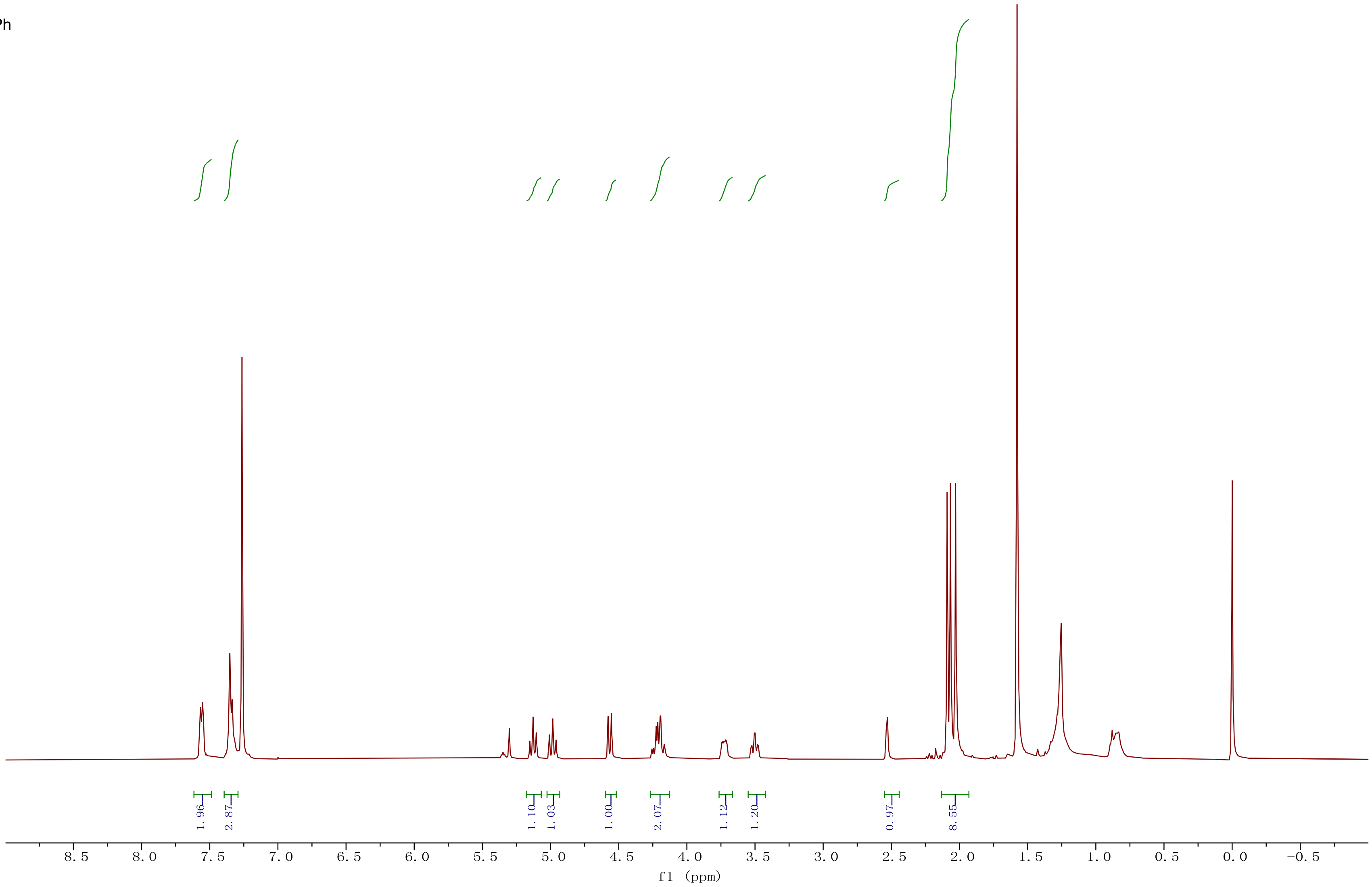

Figure S27. ^{1}H NMR spectrum (400 MHz) of **8** in $CDCl_3$

Phenyl 3,4,6-tri-*O*-acetyl-1-thio-β-D-glucopyranoside 8

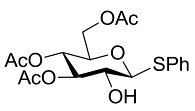

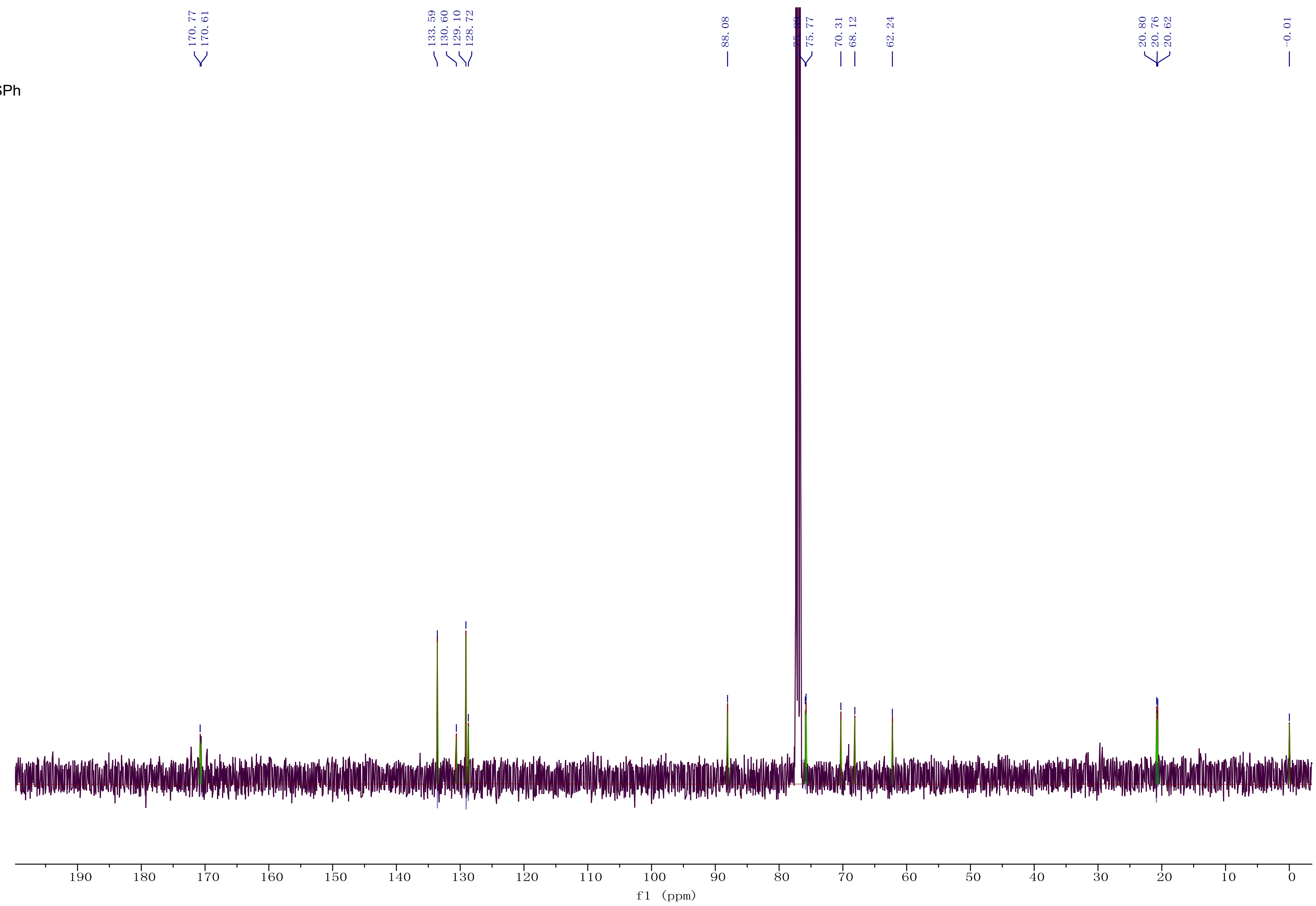

Figure S28. ^{13}C NMR spectrum (100 MHz) of **8** in $CDCl_3$

Phenyl 3,4,6-tri-*O*-ethyl-1-thio-β-D-glucopyranoside 9

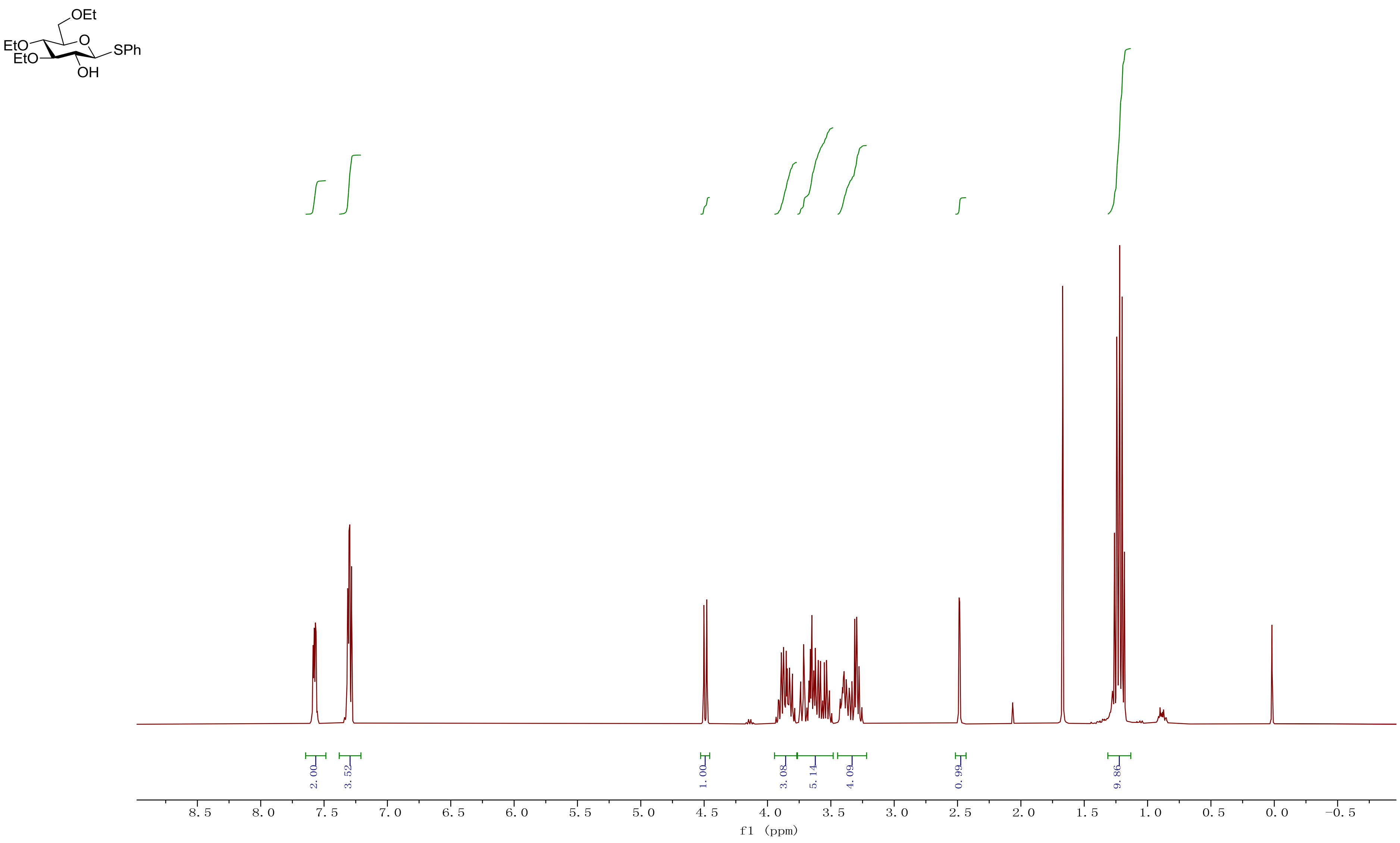

Figure S29. ^{1}H NMR spectrum (400 MHz) of **9** in $CDCl_3$

Phenyl 3,4,6-tri-*O*-ethyl-1-thio-β-D-glucopyranoside 9

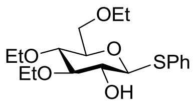

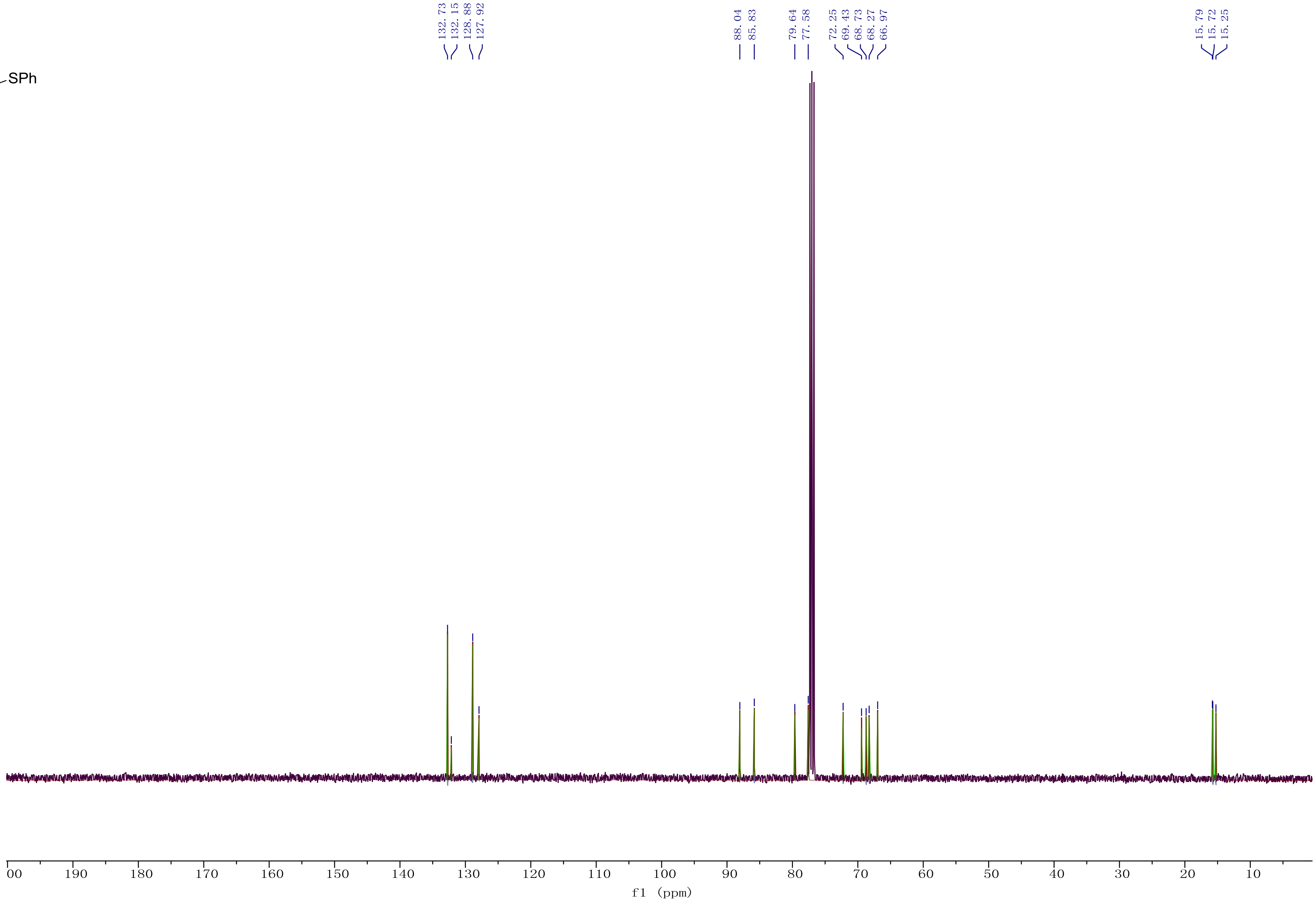

Figure S30. ^{13}C NMR spectrum (100 MHz) of **9** in $CDCl_3$

Phenyl 3,4,6-tri-*O*-tert-butyl-dimethylsily-1-thio-β-D-glucopyranoside 10

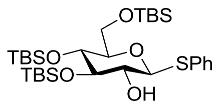

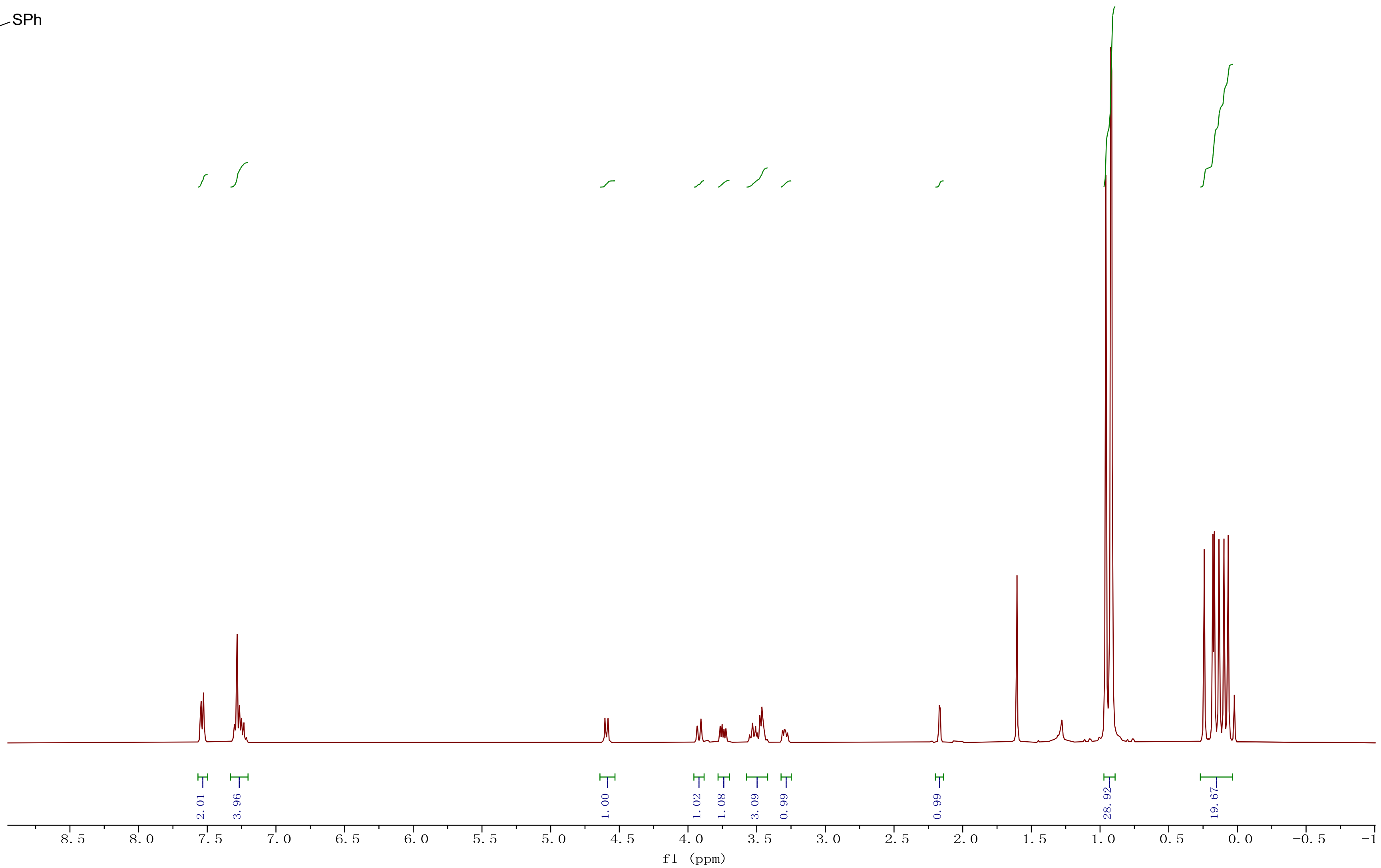

Figure S31. ^{1}H NMR spectrum (400 MHz) of **10** in $CDCl_3$

Phenyl 3,4,6-tri-*O*-tert-butyl-dimethylsily-1-thio-β-D-glucopyranoside 10

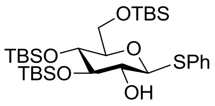

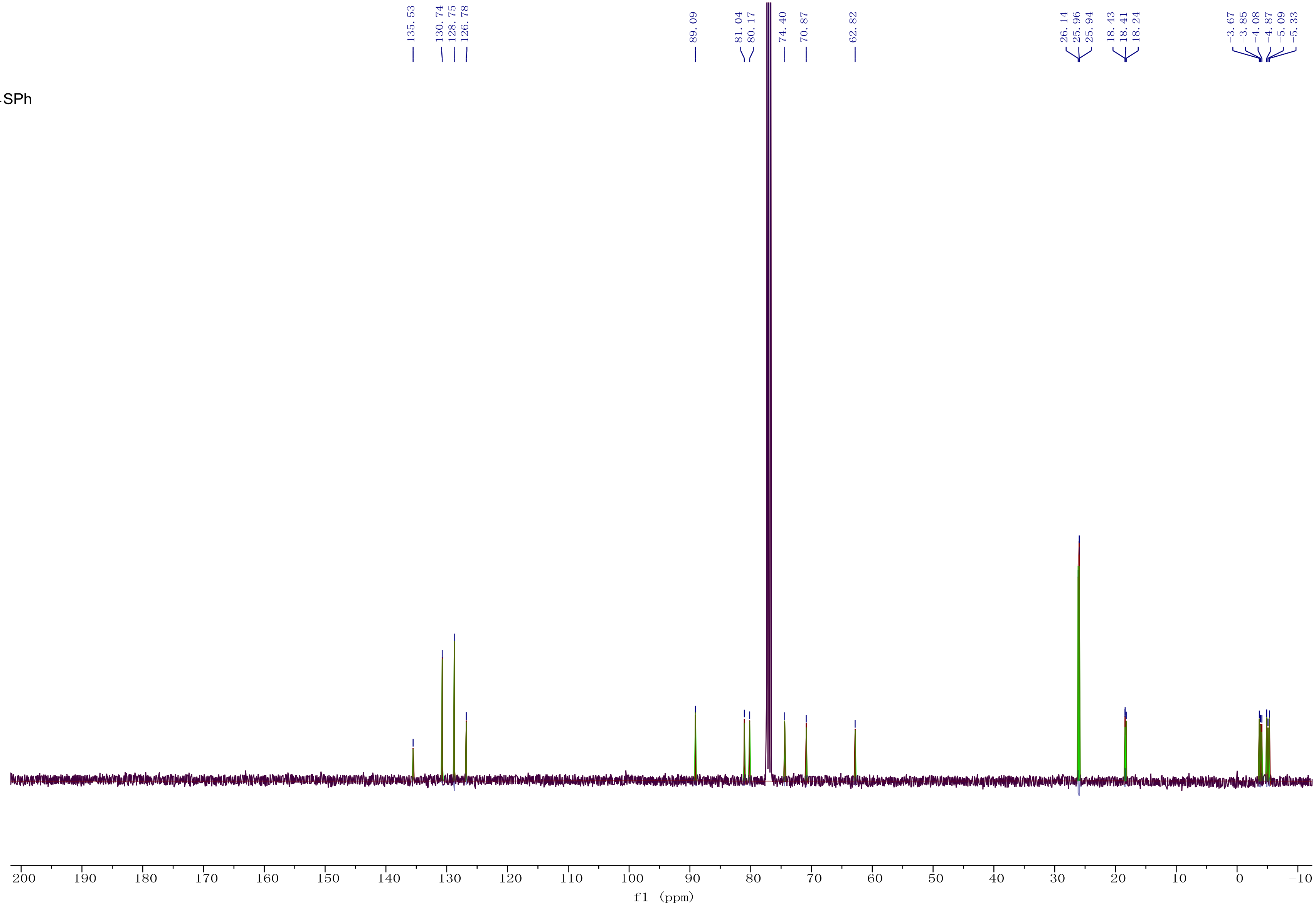

Figure S32. ^{13}C NMR spectrum (100 MHz) of **10** in $CDCl_3$

Phenyl 3,4,6-tri-*O*-*p*-methoxybenzyl-1-thio-β-D-glucopyranoside 11

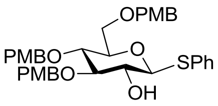

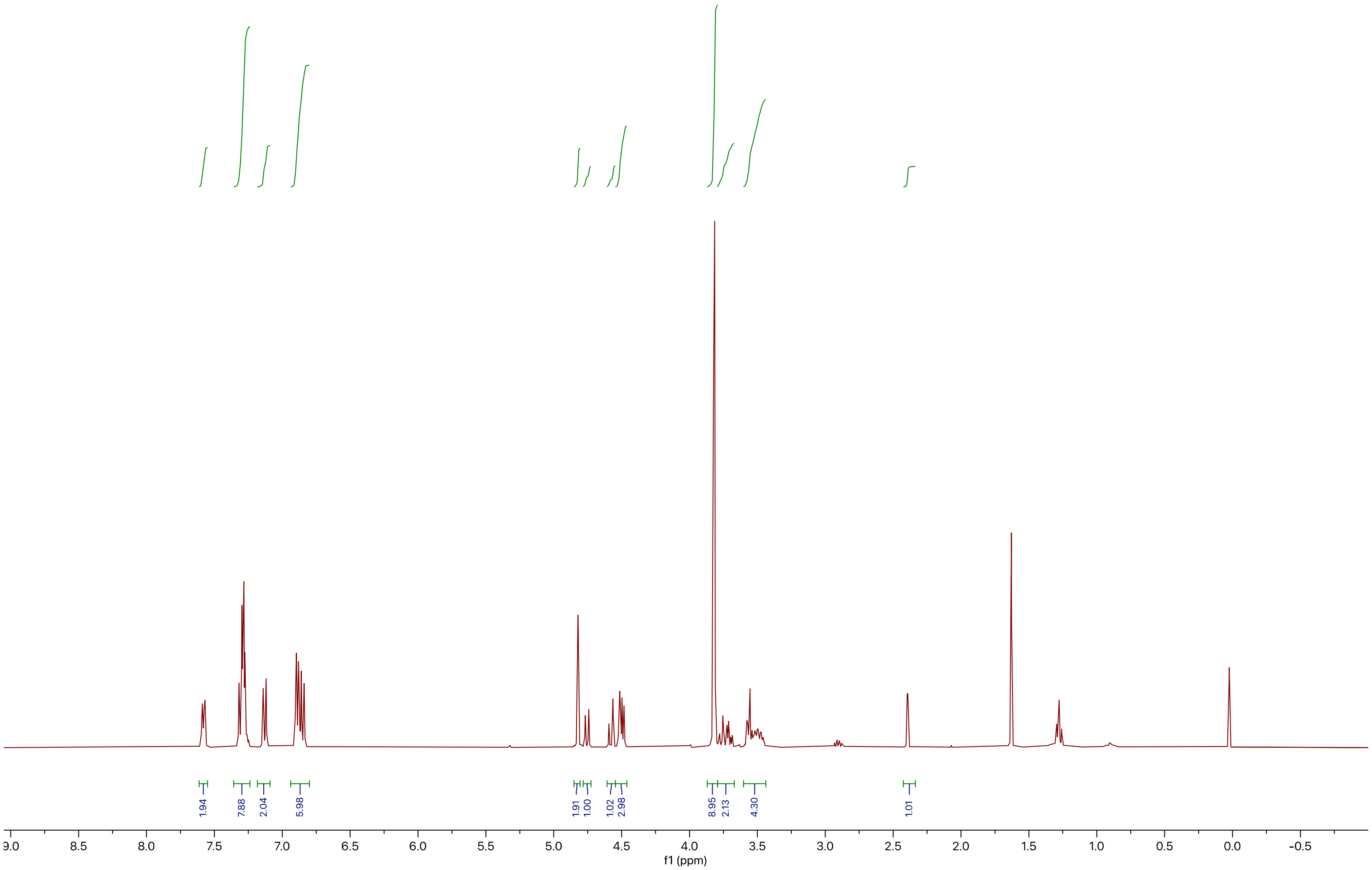

Figure S33. ^{1}H NMR spectrum (400 MHz) of **11** in $CDCl_3$

Phenyl 3,4,6-tri-*O*-*p*-methoxybenzyl-1-thio-β-D-glucopyranoside 11

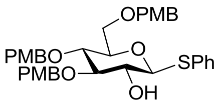

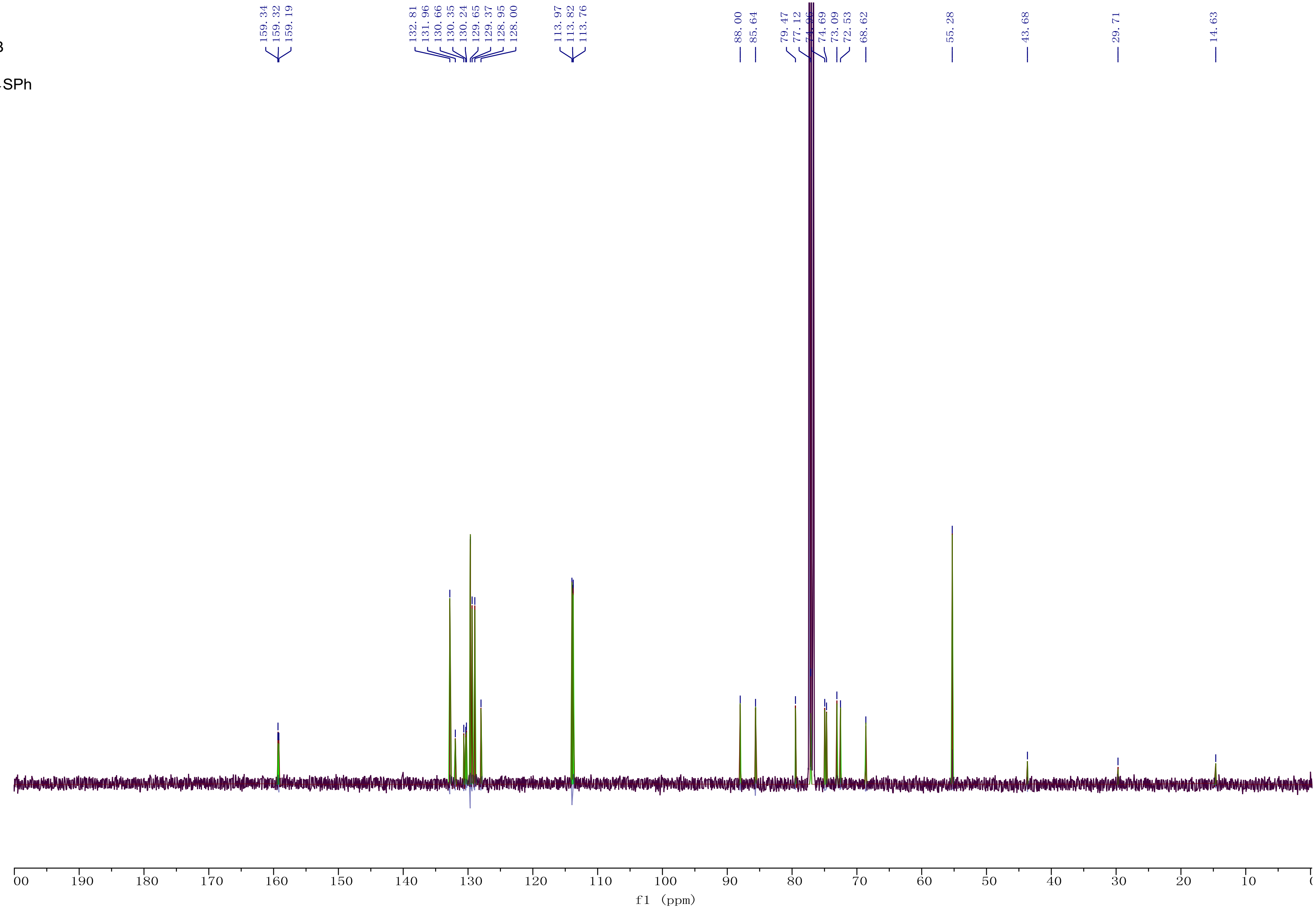

Figure S34. ^{13}C NMR spectrum (100 MHz) of **11** in $CDCl_3$

Phenyl 3,4,6-tri-*O*-benzyl-1-thio-β-D-galactopyranoside 12

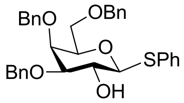

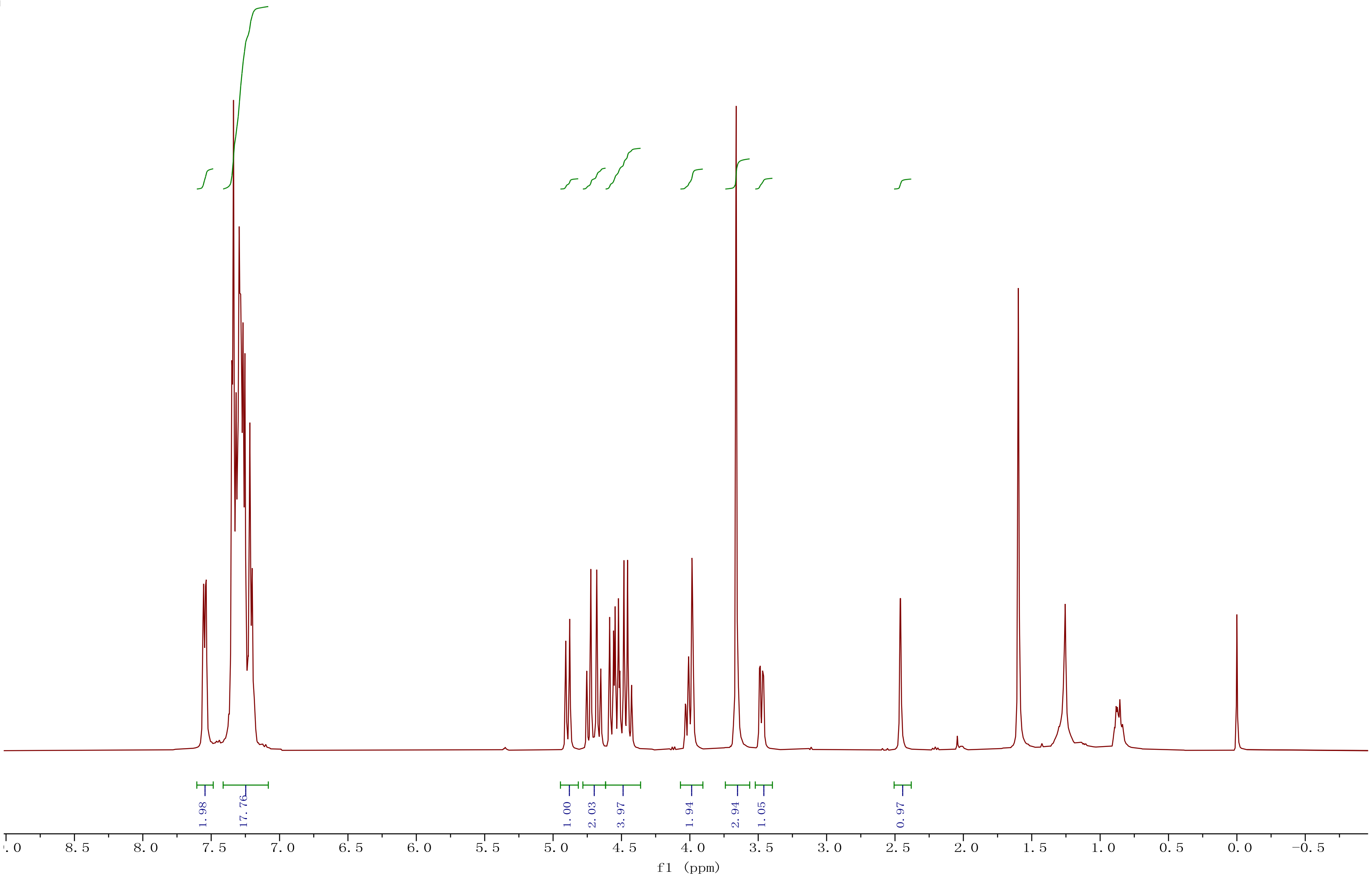

Figure S35. ¹H NMR spectrum (400 MHz) of **12** in $CDCl_3$

Phenyl 3,4,6-tri-*O*-benzyl-1-thio-β-D-galactopyranoside 12

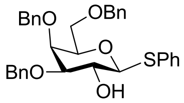

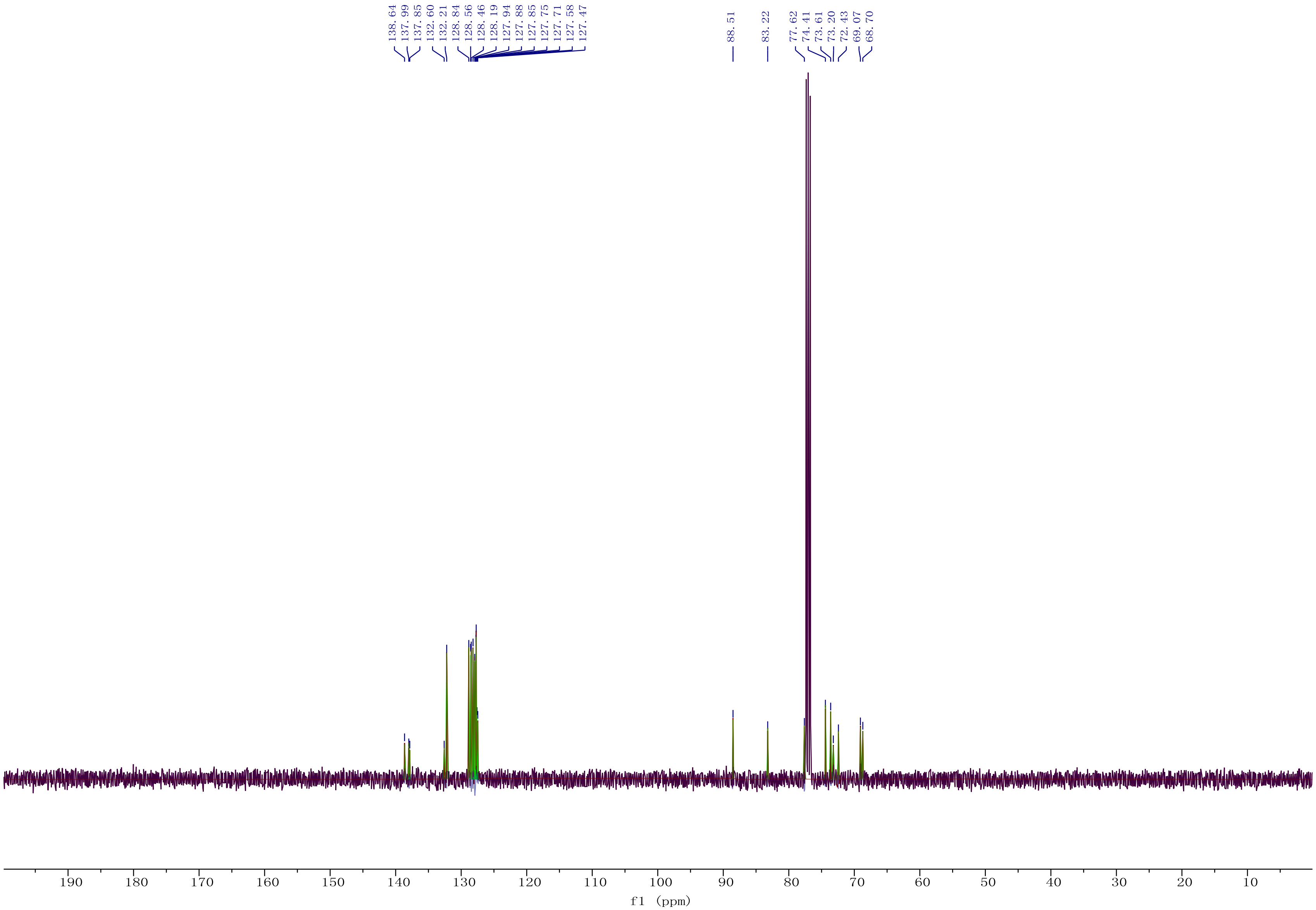

Figure S36. ^{13}C NMR spectrum (100 MHz) of **12** in $CDCl_3$

Phenyl 3,4,6-tri-*O*-benzyl-1-thio-β-D-galactopyranoside 12

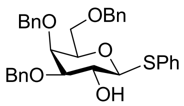

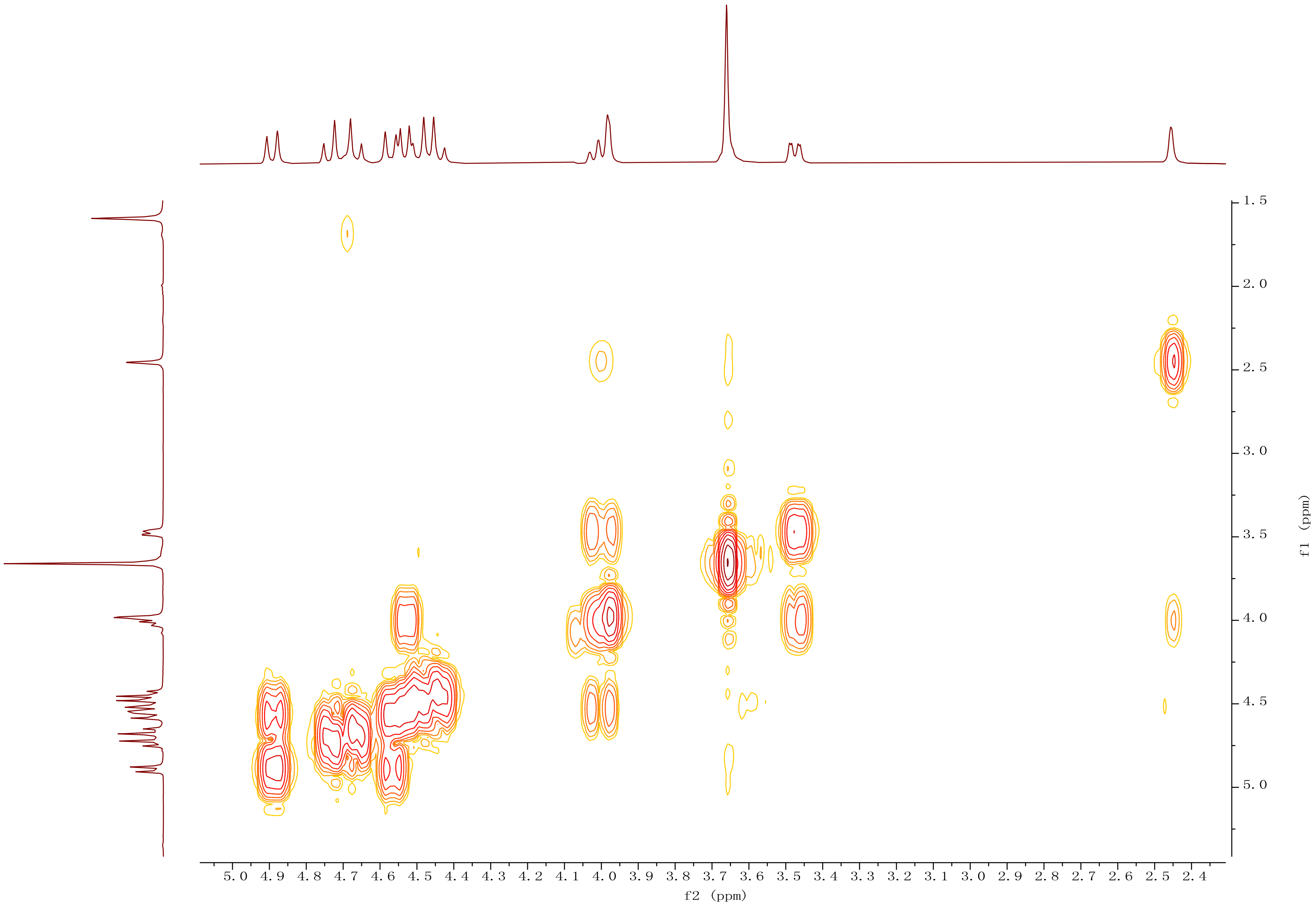

Figure S37. ^{1}H-^{1}H COSY spectrum of **12** in $CDCl_3$

Phenyl 3,4-di-*O*-benzyl-6-*O*-tert-butyl-dimethylsily-1-thio-β-D-galactopyranoside 13

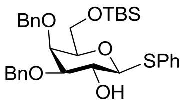

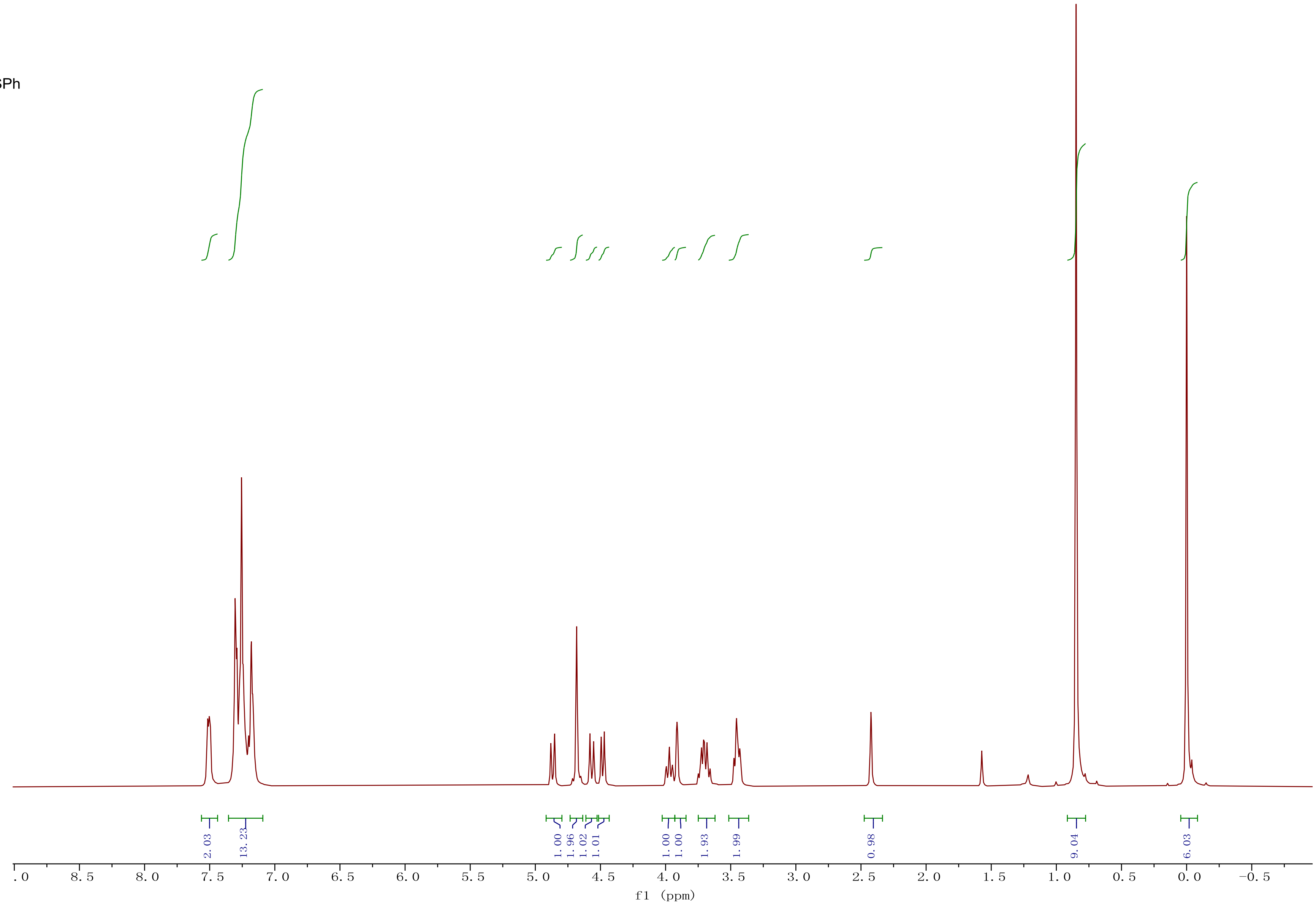

Figure S38. ^{1}H NMR spectrum (400 MHz) of **13** in CDCl

Phenyl 3,4-di-*O*-benzyl-6-*O*-tert-butyl-dimethylsily-1-thio-β-D-galactopyranoside 13

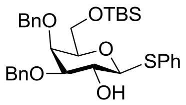

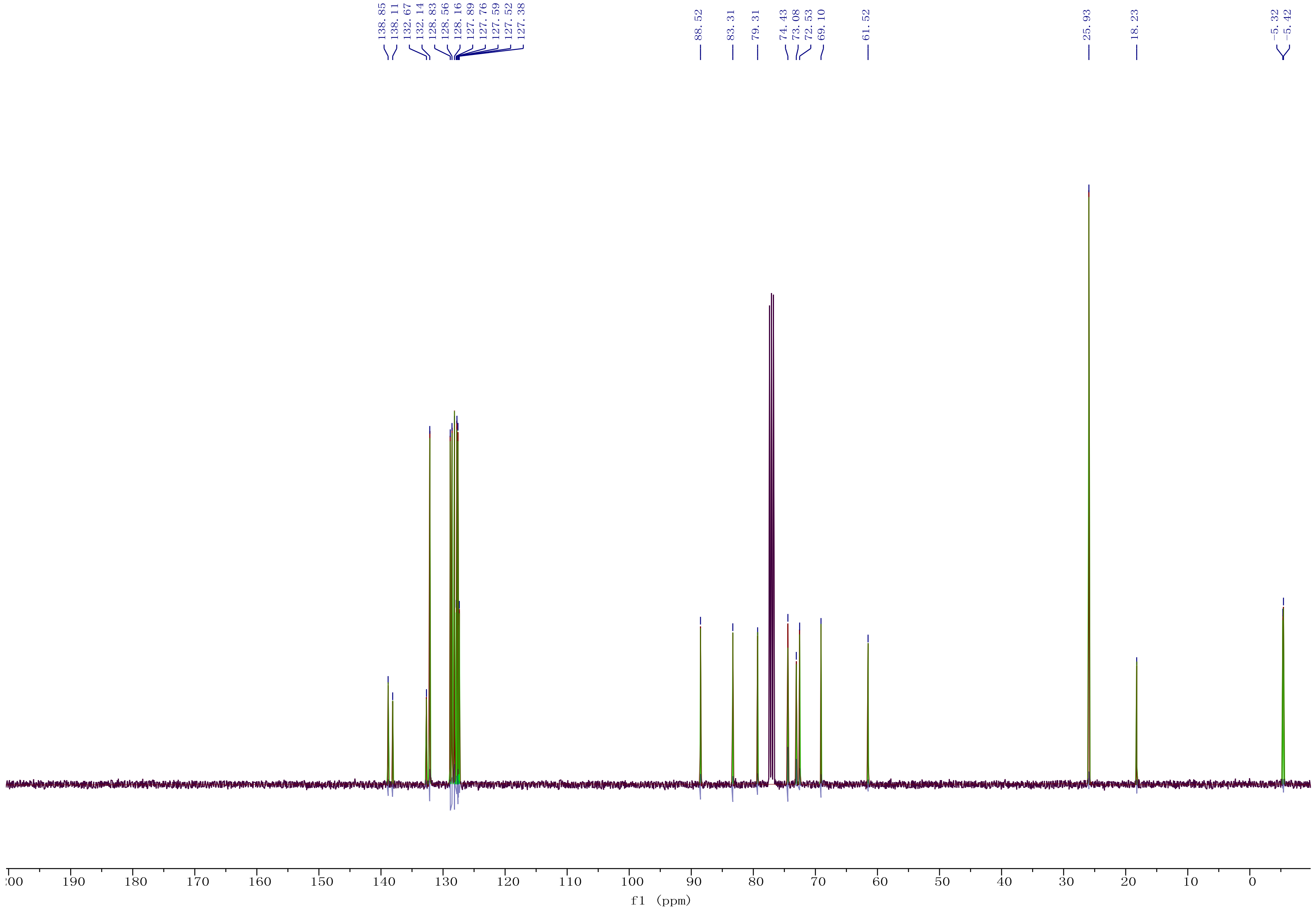

Figure S39. ^{13}C NMR spectrum (100 MHz) of **13** in $CDCl_3$

Phenyl 3-*O*-benzyl-4-*O*-acetyl-6-*O*-tert-butyl-dimethylsily-1-thio-β-D-galactopyranoside 14

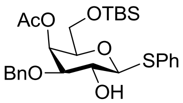

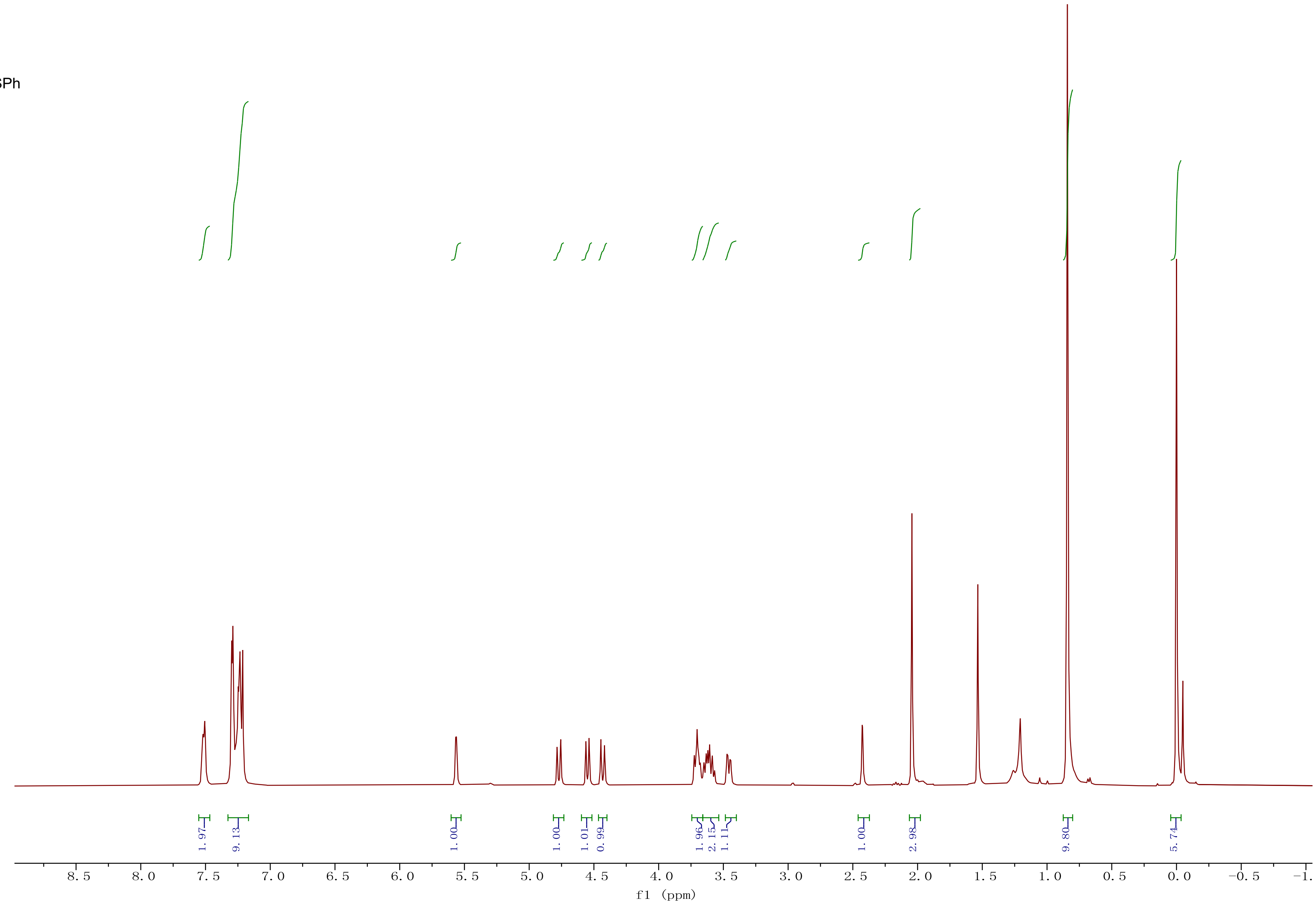

Figure S40. ^{1}H NMR spectrum (400 MHz) of **14** in $CDCl_3$

Phenyl 3,4-di-*O*-benzyl-6-*O*-tert-butyl-dimethylsily-1-thio-β-D-galactopyranoside 14

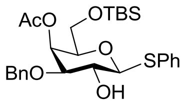

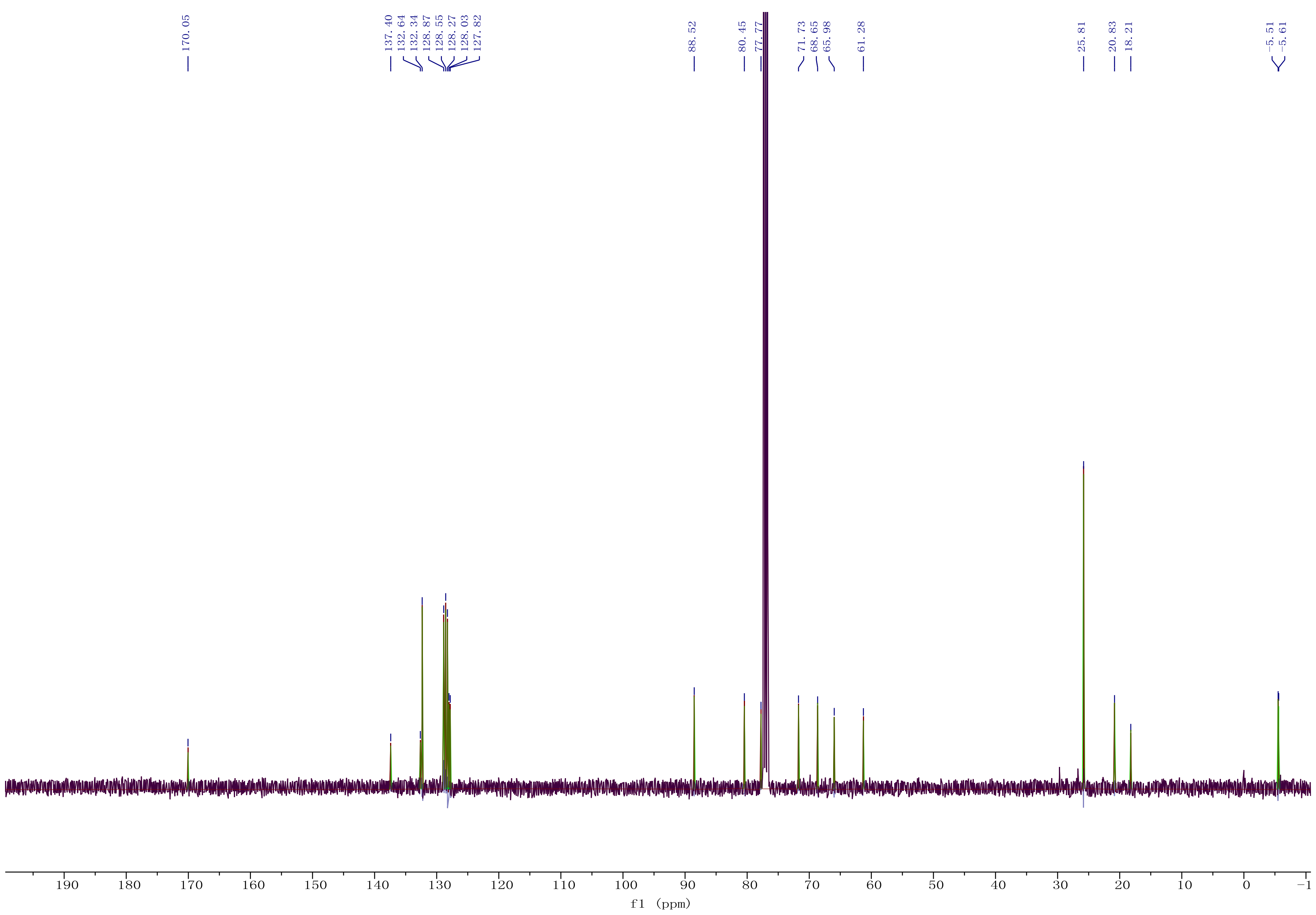

Figure S41. ^{13}C NMR spectrum (100 MHz) of **14** in $CDCl_3$

Phenyl 3,4-di-*O*-benzyl-1-thio-β-D-xylal 15

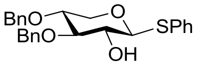

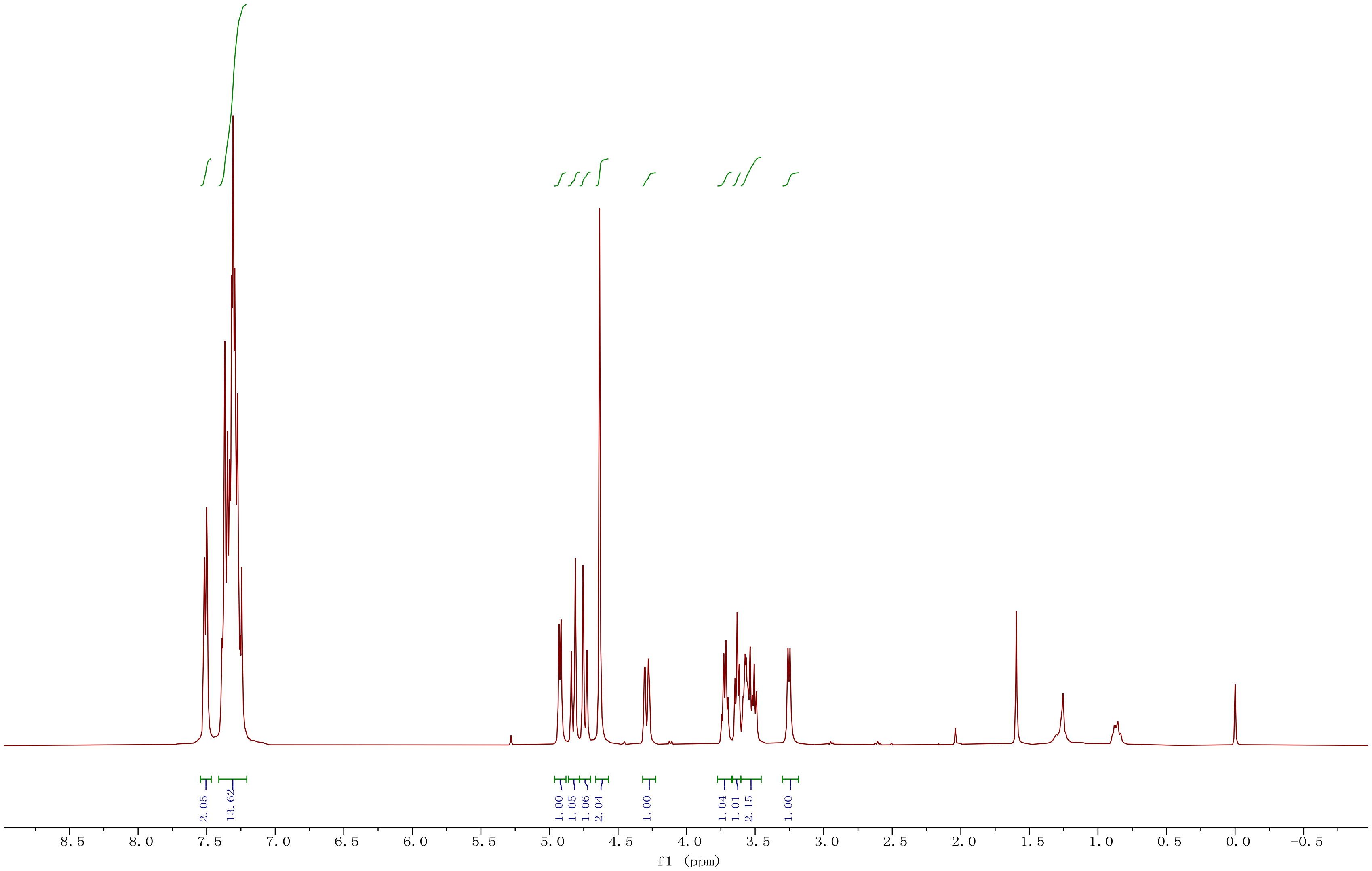

Figure S42. ^{1}H NMR spectrum (400 MHz) of **15** in $CDCl_3$

Phenyl 3,4-di-*O*-benzyl-1-thio-β-D-xylal 15

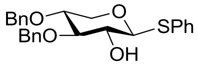

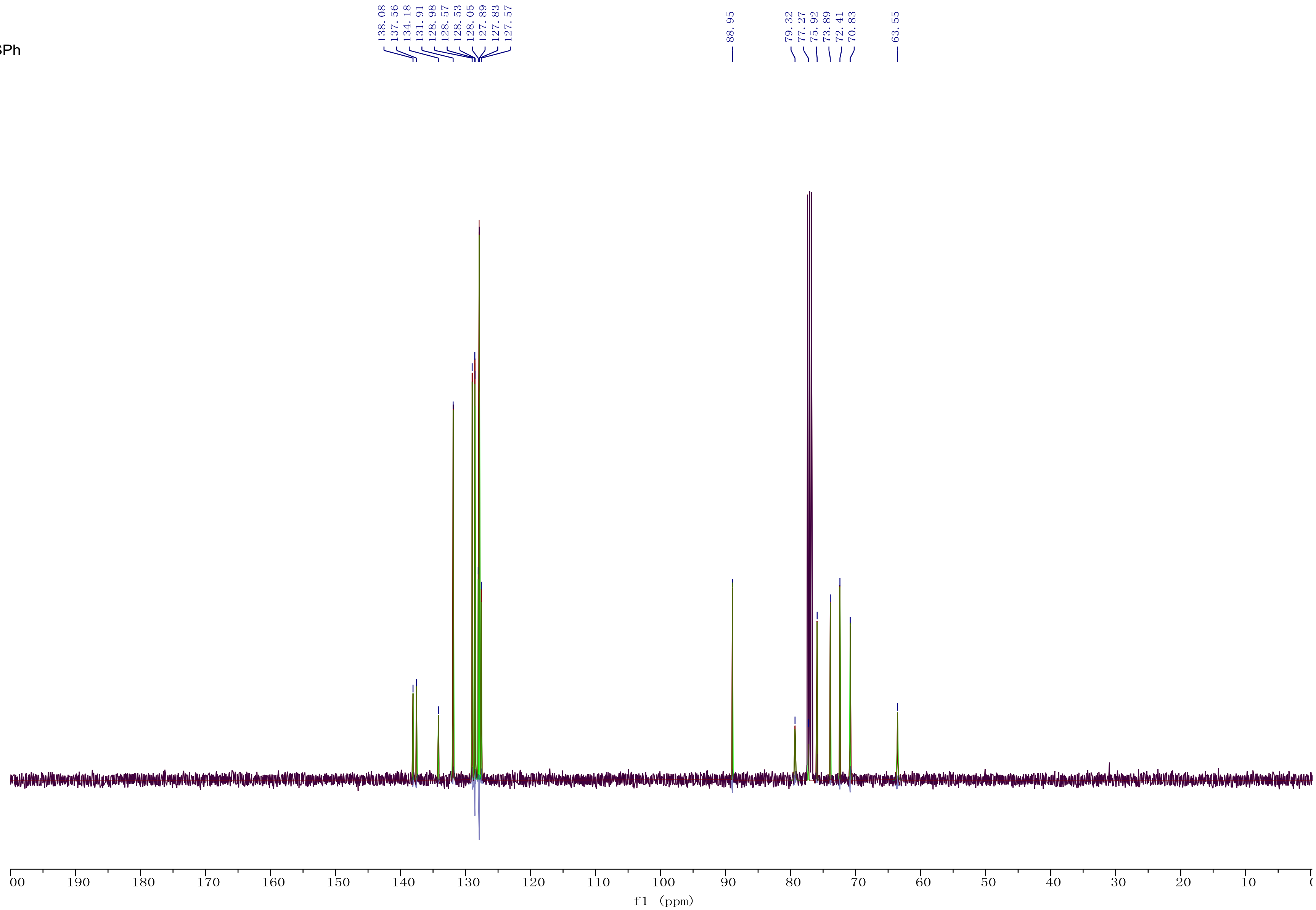

Figure S43. ^{13}C NMR spectrum (100 MHz) of **15** in $CDCl_3$

Phenyl 3,4-di-*O*-benzyl-1-thio-β-D-xylal 15

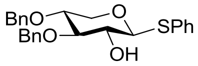

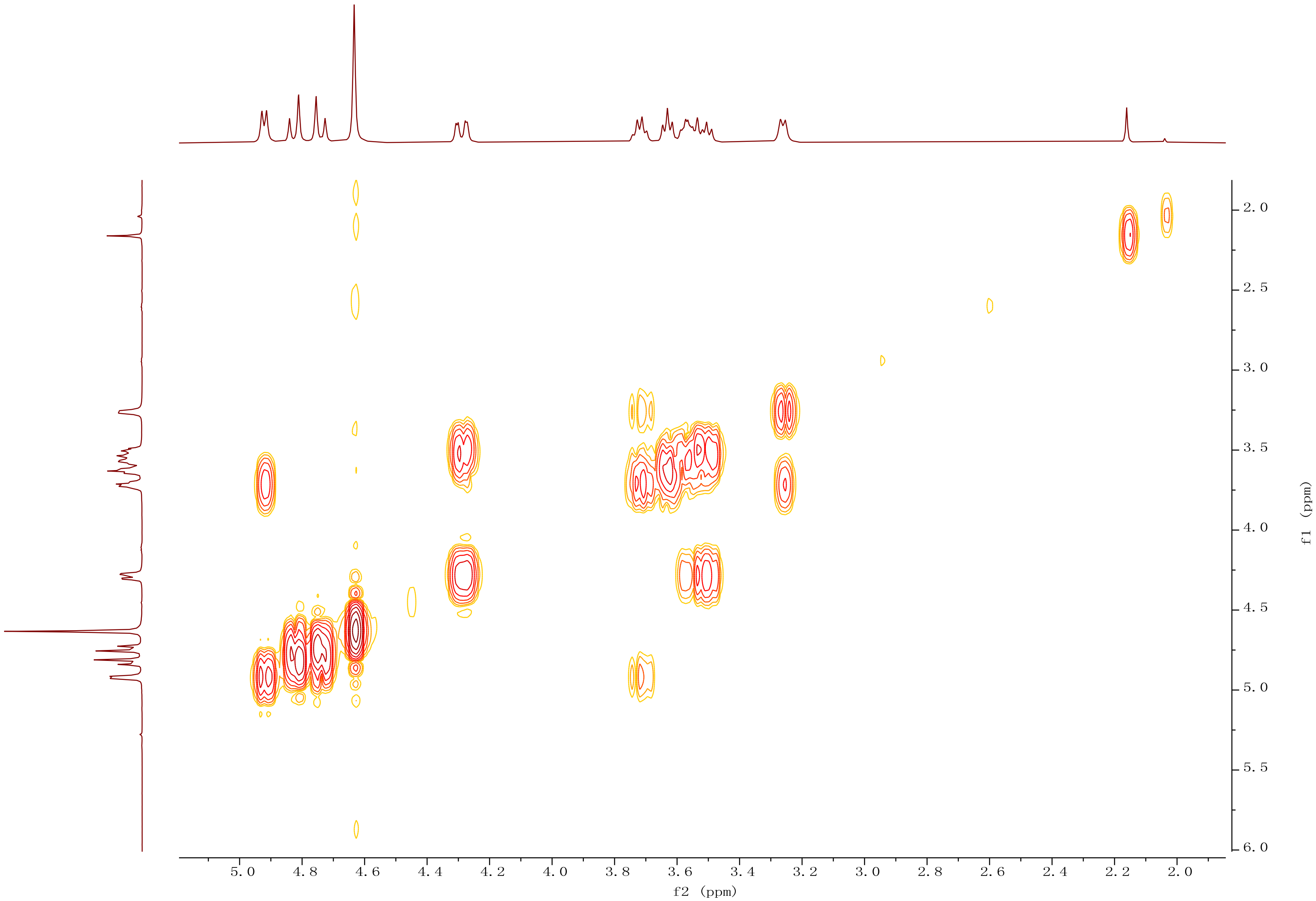

Figure S44. ${}^1H$-${}^1H$ COSY spectrum of **15** in $CDCl_3$

Phenyl 2,3,3′,4,6,6′-hexa-*O*-benzyl-D-1-thio-β-lactoside 16

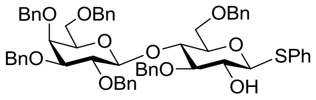

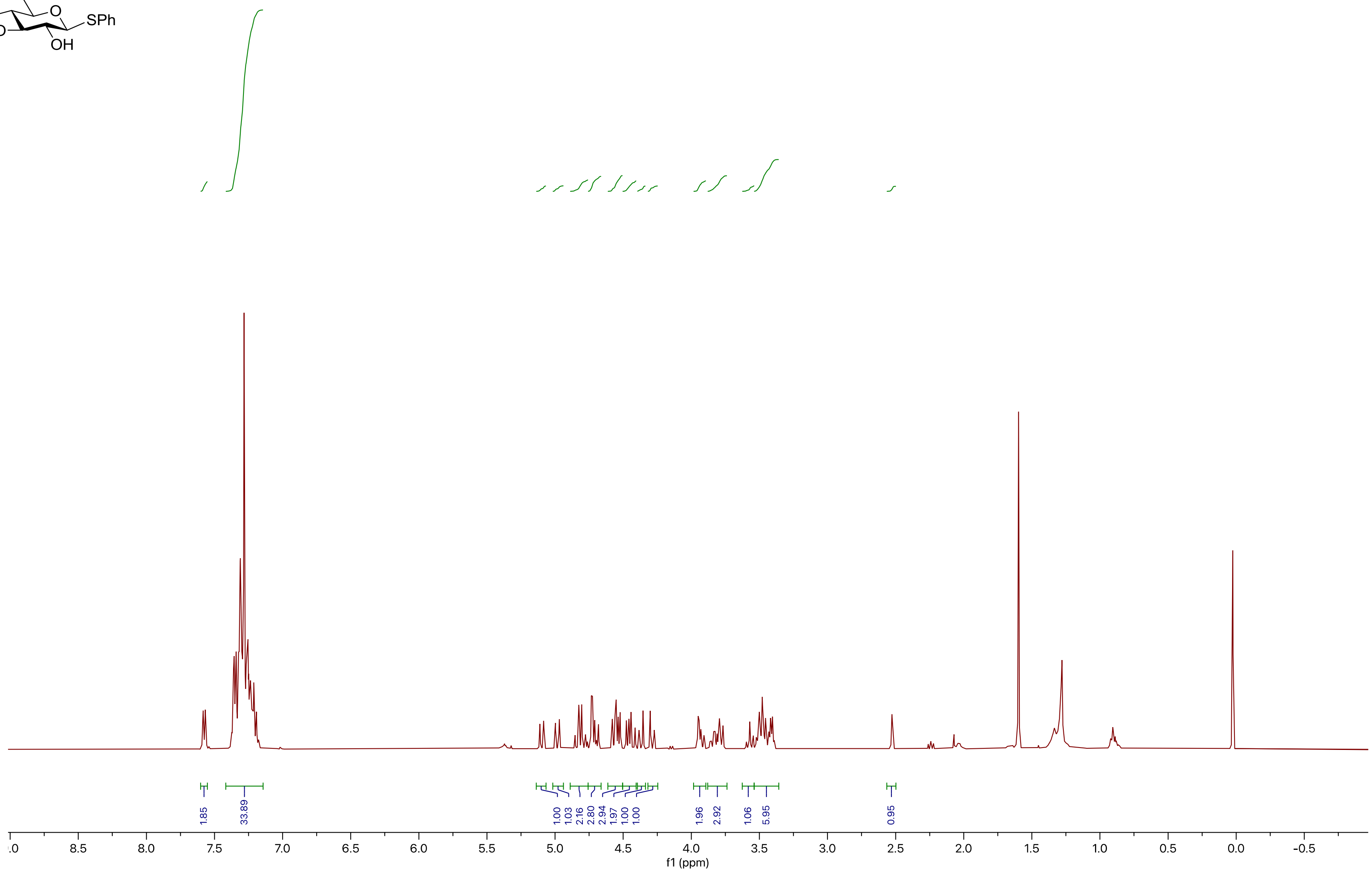

Figure S45. ^{1}H NMR spectrum (400 MHz) of **16** in $CDCl_3$

Phenyl 2,3,3′,4,6,6′-hexa-*O*-benzyl-D-1-thio-β-lactoside 16

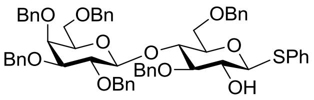

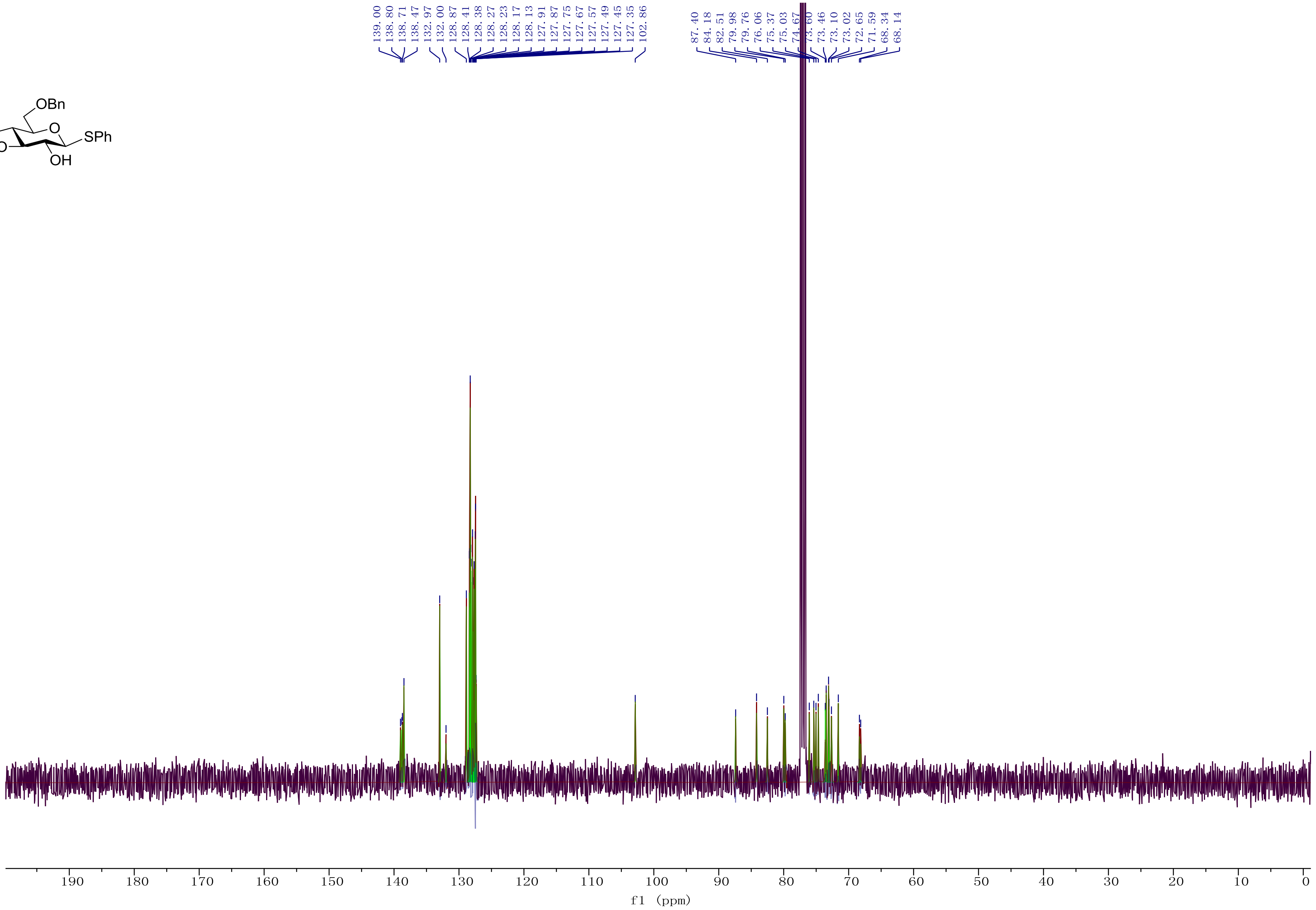

Figure S46. ^{13}C NMR spectrum (100 MHz) of **16** in $CDCl_3$

Phenyl 2,3,3′,4,6,6′-hexa-*O*-benzyl-D-1-thio-β-lactoside 16

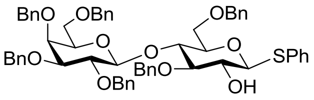

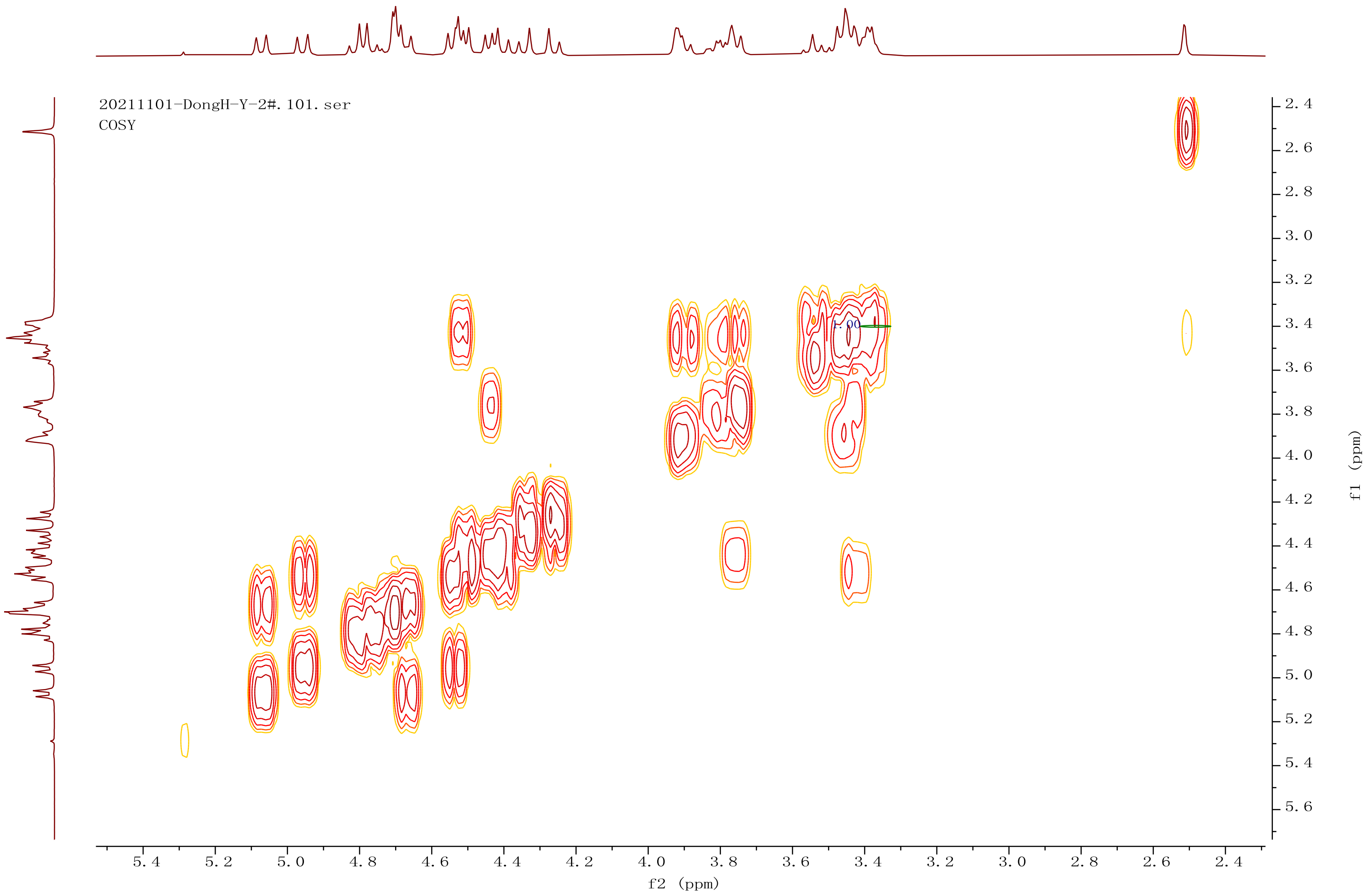

Figure S47. ^{1}H-^{1}H COSY spectrum of **16** in $CDCl_3$

4-Methylphenyl 3,4,6-tri-O-benzyl-1-thio-β-D-glucopyranoside 17

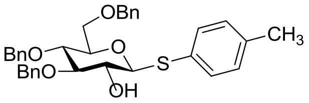

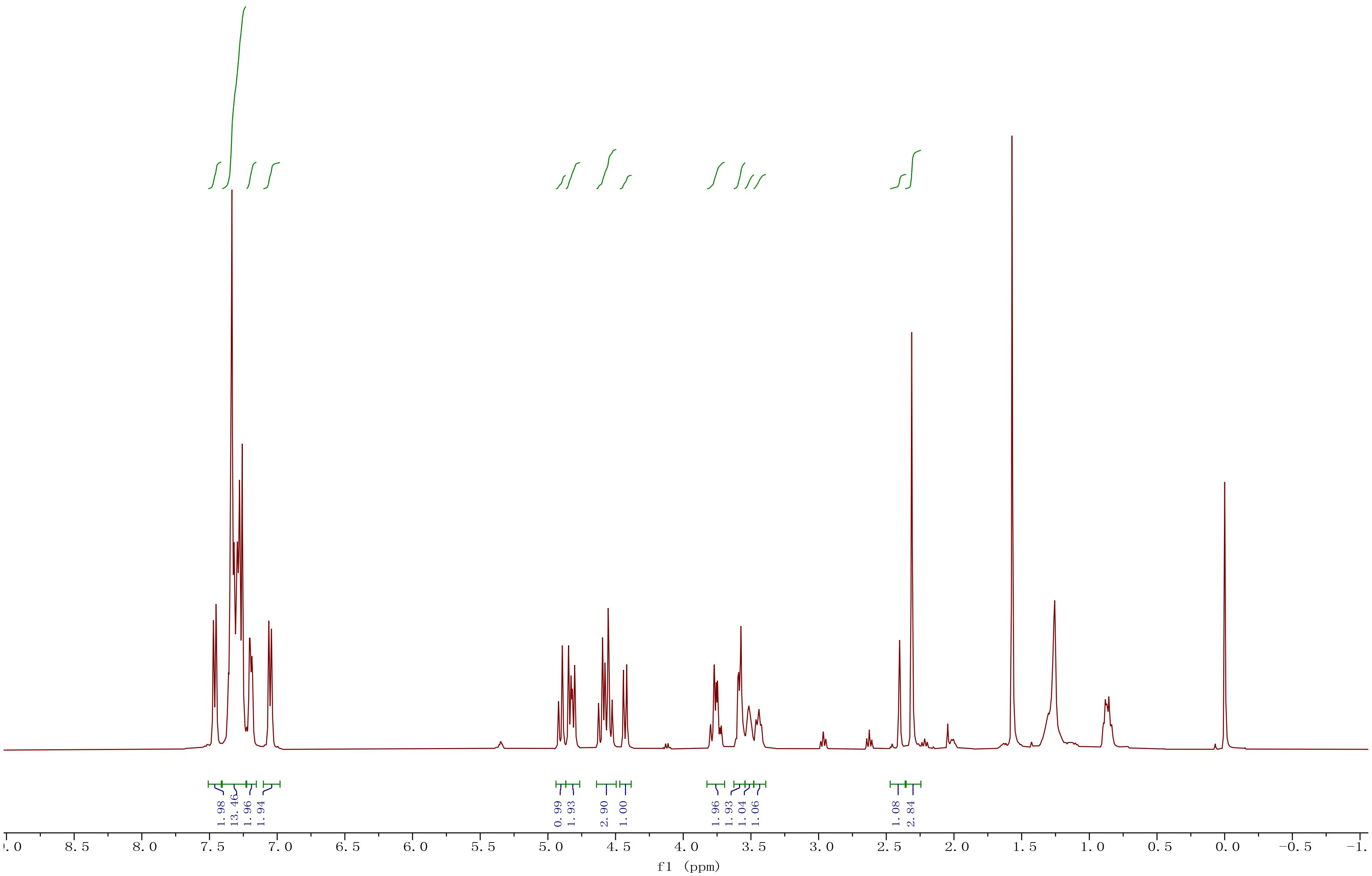

Figure S48. ^{1}H NMR spectrum (400 MHz) of **17** in $CDCl_3$

4-Methoxyphenyl 3,4,6-tris-*O*-benzyl-1-thio-β-D-glucopyranoside 18

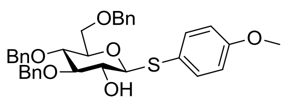

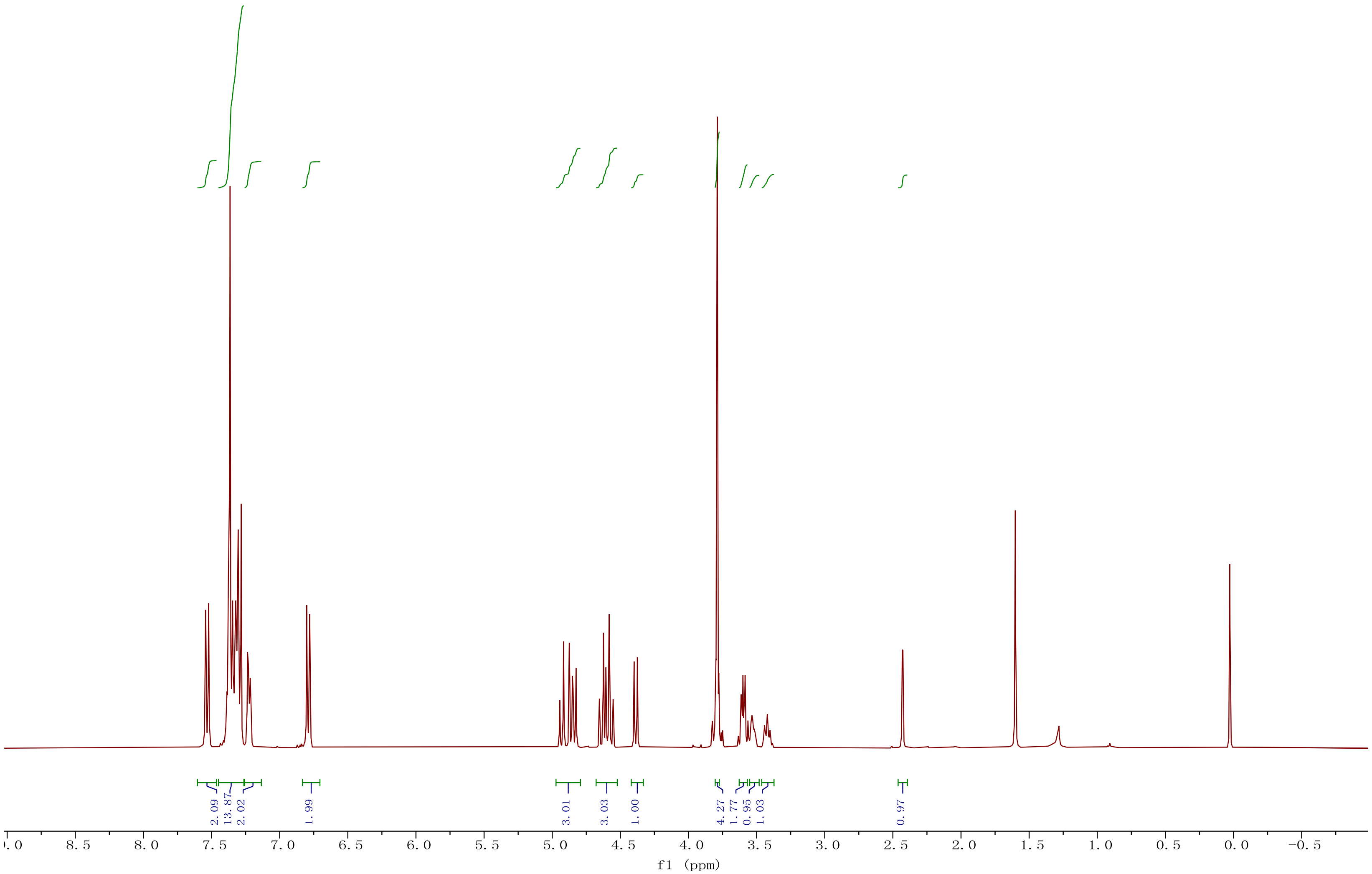

Figure S49. ^{1}H NMR spectrum (400 MHz) of **18** in $CDCl_3$

4-Chlorophenyl 3,4,6-tri-*O*-benzyl-1-thio-β-D-glucopyranoside 19

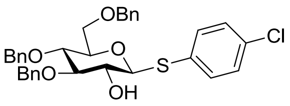

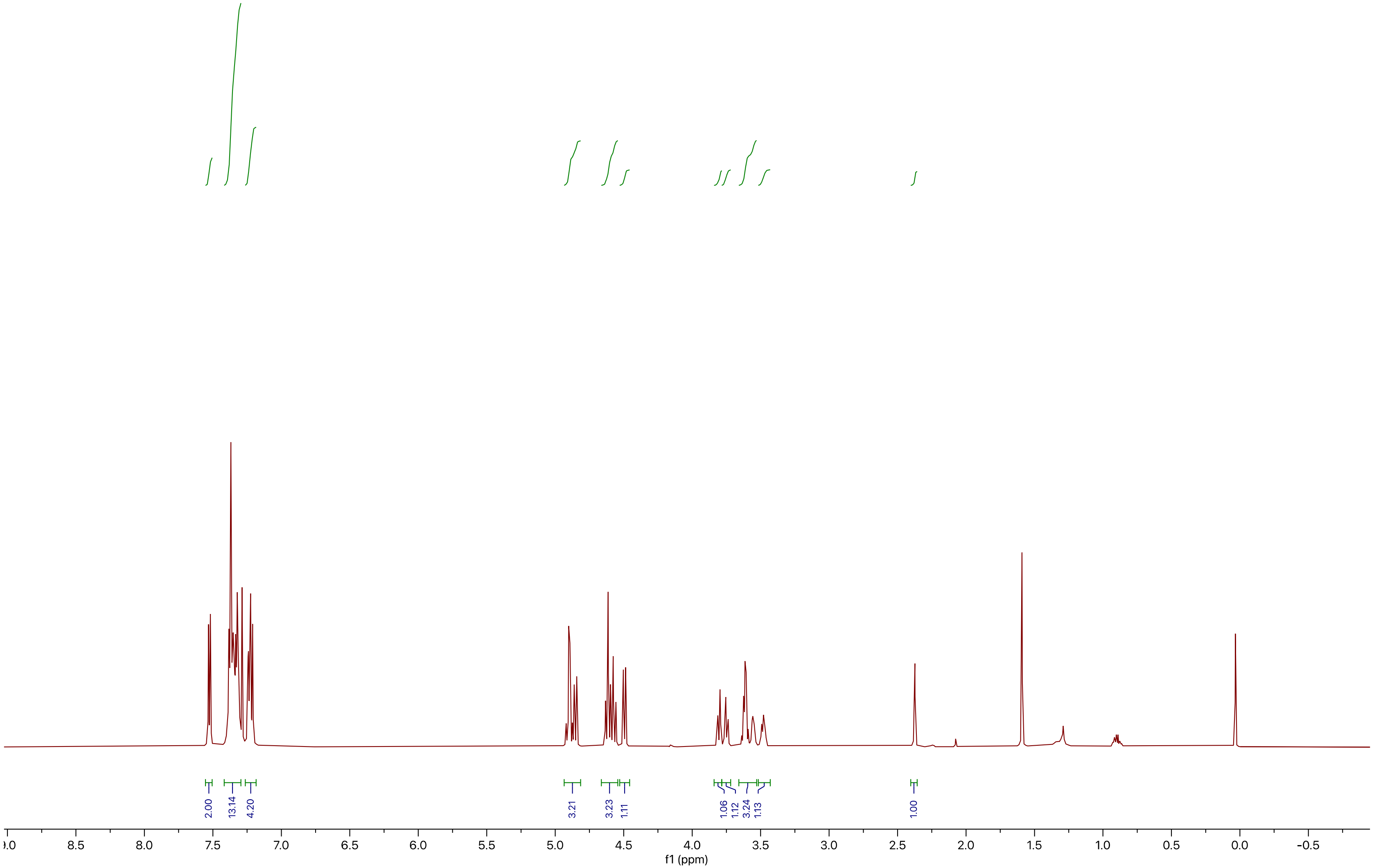

Figure S50. ¹H NMR spectrum (400 MHz) of **19** in $CDCl_3$

4-Chlorophenyl 3,4,6-tri-*O*-benzyl-1-thio-β-D-glucopyranoside 19

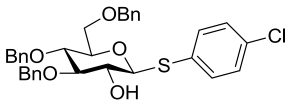

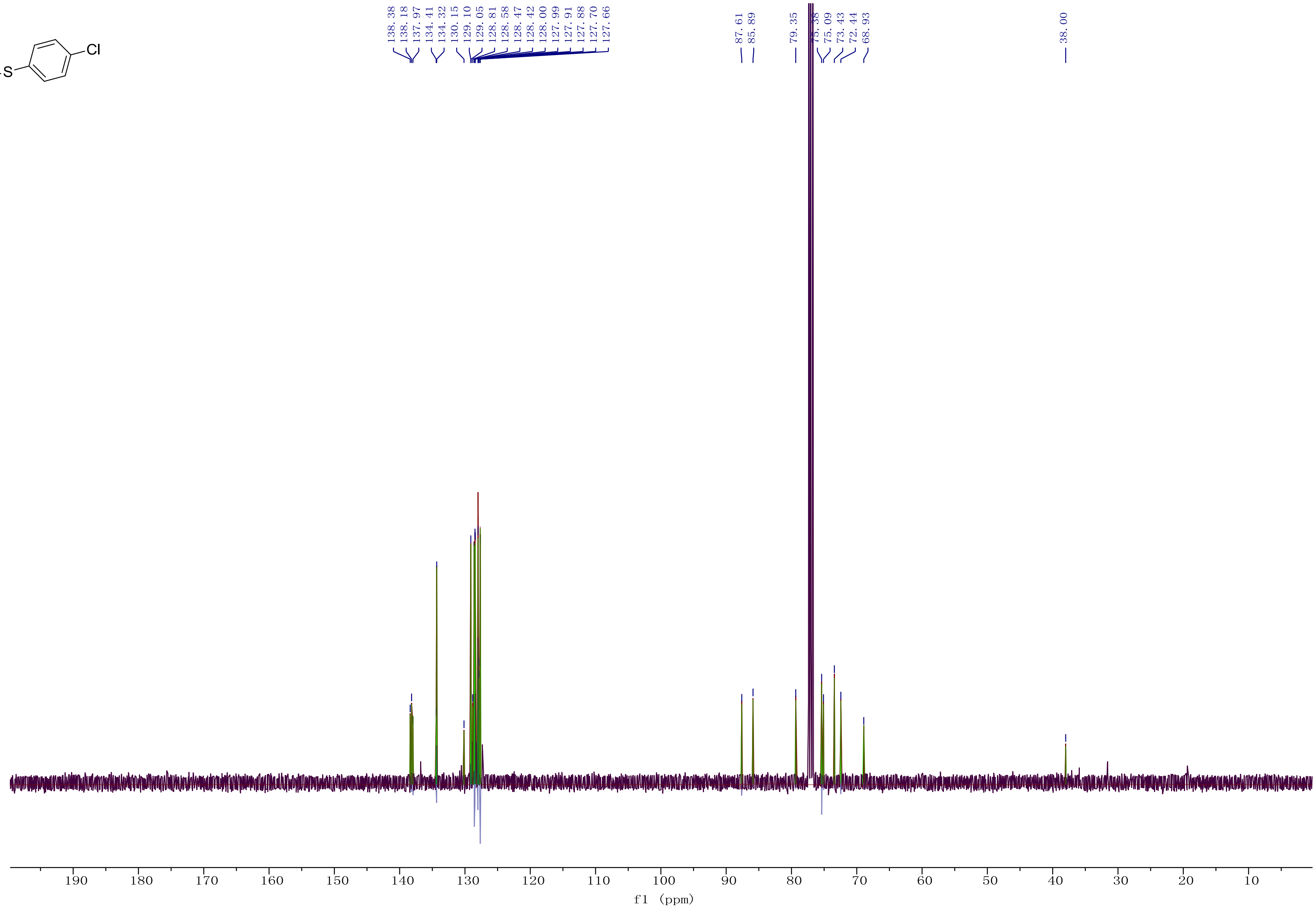

Figure S51. ^{13}C NMR spectrum (100 MHz) of **19** in $CDCl_3$

4-Aminophenyl 3,4,6-tri-*O*-benzyl-1-thio-β-D-glucopyranoside 20

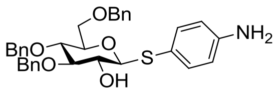

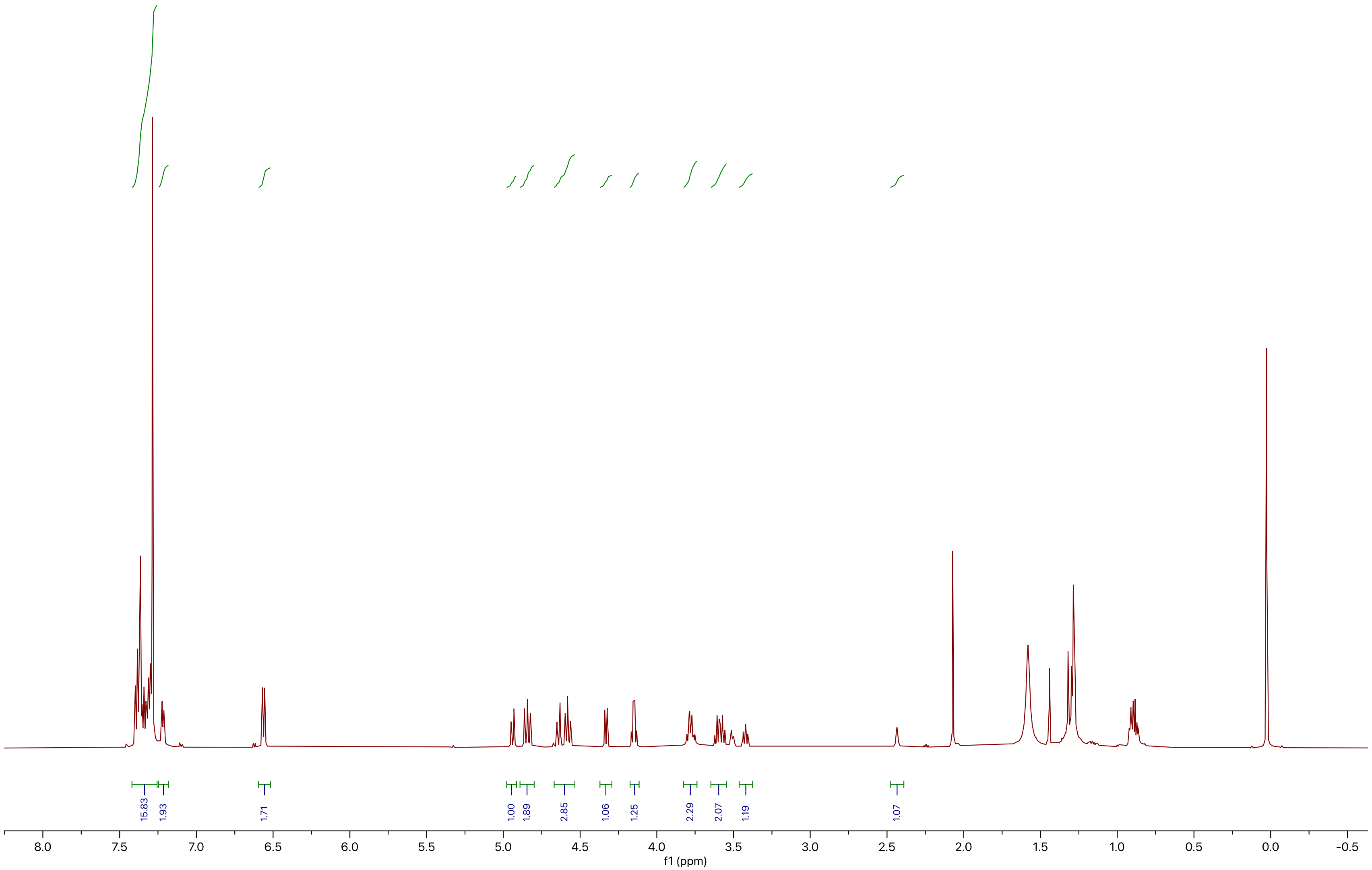

Figure S52. ^{1}H NMR spectrum (400 MHz) of **20** in $CDCl_3$

4-Aminophenyl 3,4,6-tri-*O*-benzyl-1-thio-β-D-glucopyranoside 20

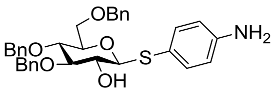

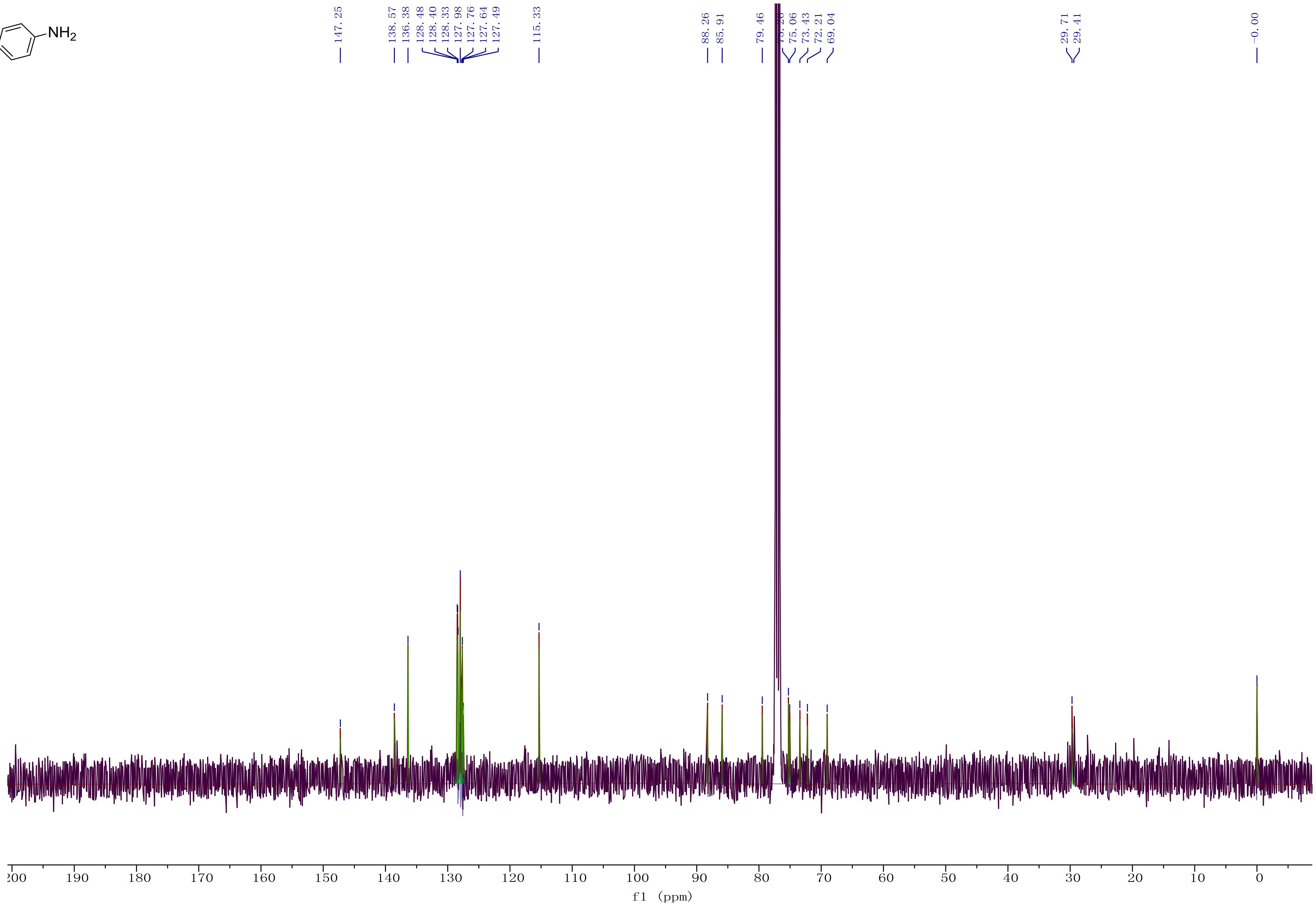

Figure S53. ^{13}C NMR spectrum (100 MHz) of **20** in $CDCl_3$

Thiophen 2-ylthio-3,4,6-tri-*O*-benzyl-1-thio-β-D-glucopyranoside 21

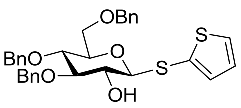

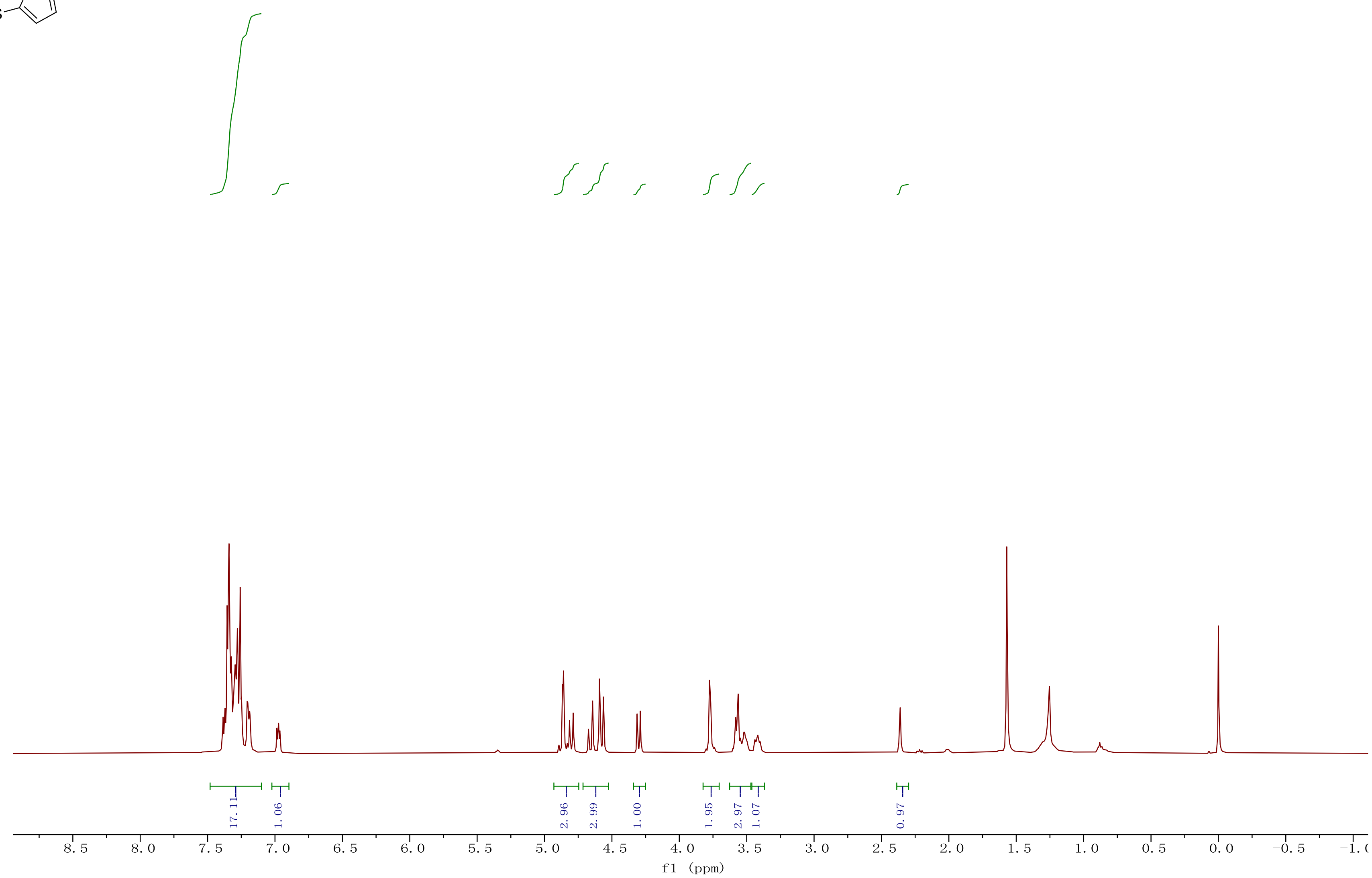

Figure S54. ^{1}H NMR spectrum (400 MHz) of **21** in $CDCl_3$

Thiophen 2-ylthio-3,4,6-tri-*O*-benzyl-1-thio-β-D-glucopyranoside 21

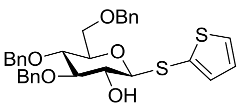

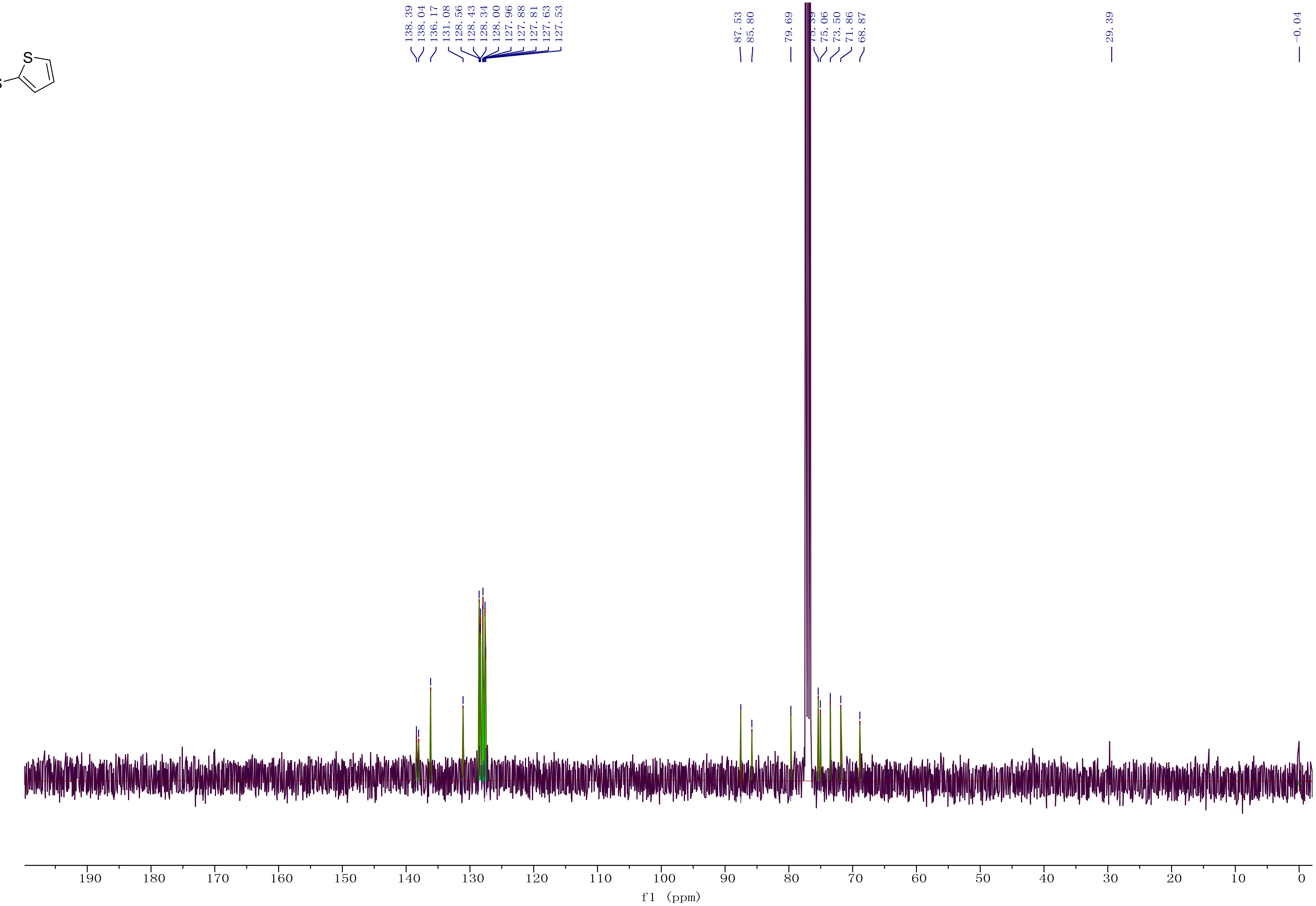

Figure S55. ^{13}C NMR spectrum (100 MHz) of **21** in $CDCl_3$

Benzothiazol-2-yl 3,4,6-tri-*O*-benzyl-1-thio-β-D-glucopyranoside 22

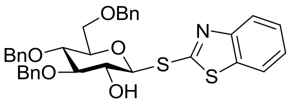

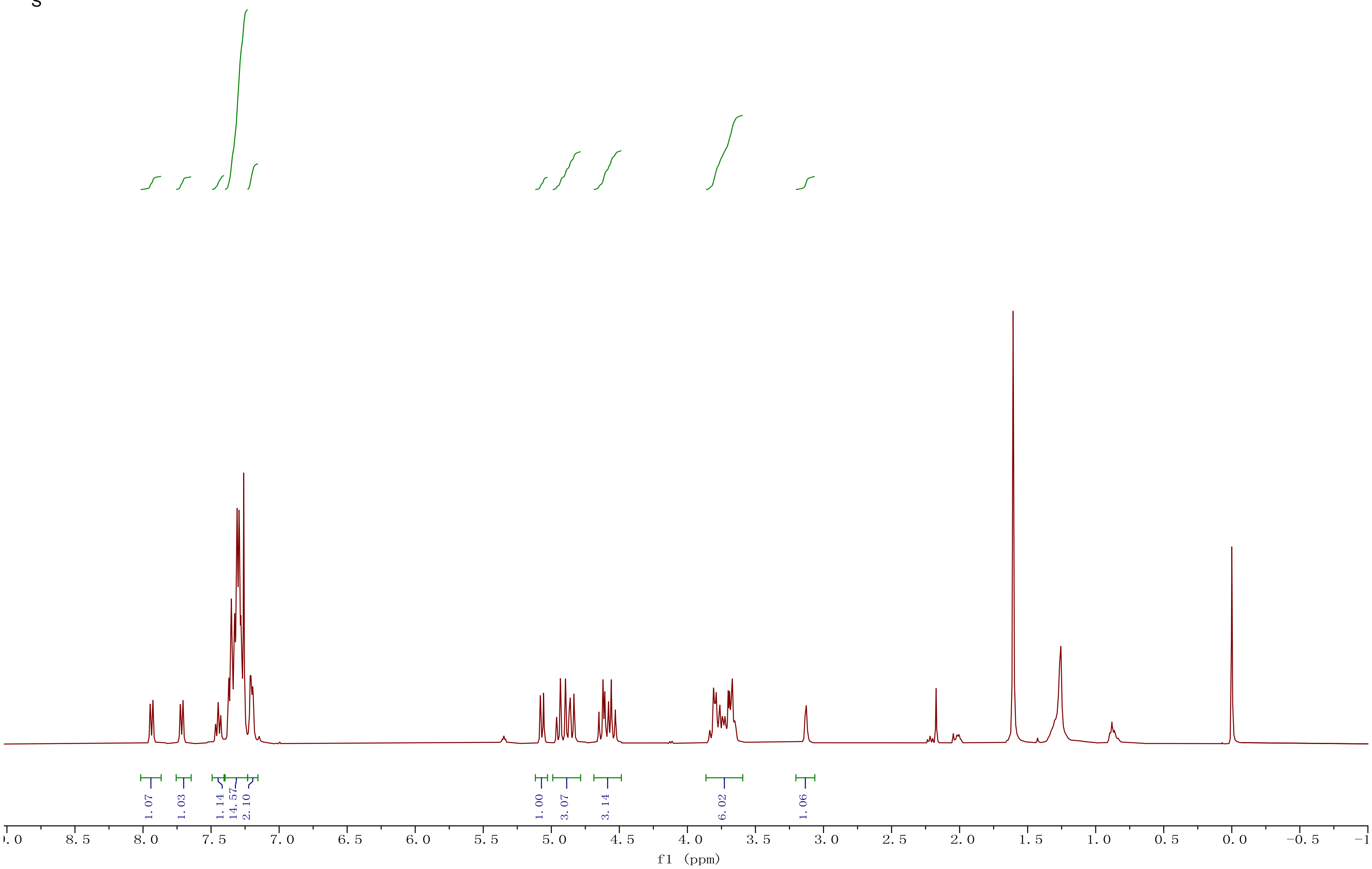

Figure S56. ^{1}H NMR spectrum (400 MHz) of **22** in $CDCl_3$

Benzothiazol-2-yl 3,4,6-tri-*O*-benzyl-1-thio-β-D-glucopyranoside 22

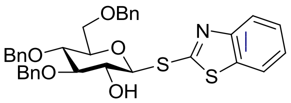

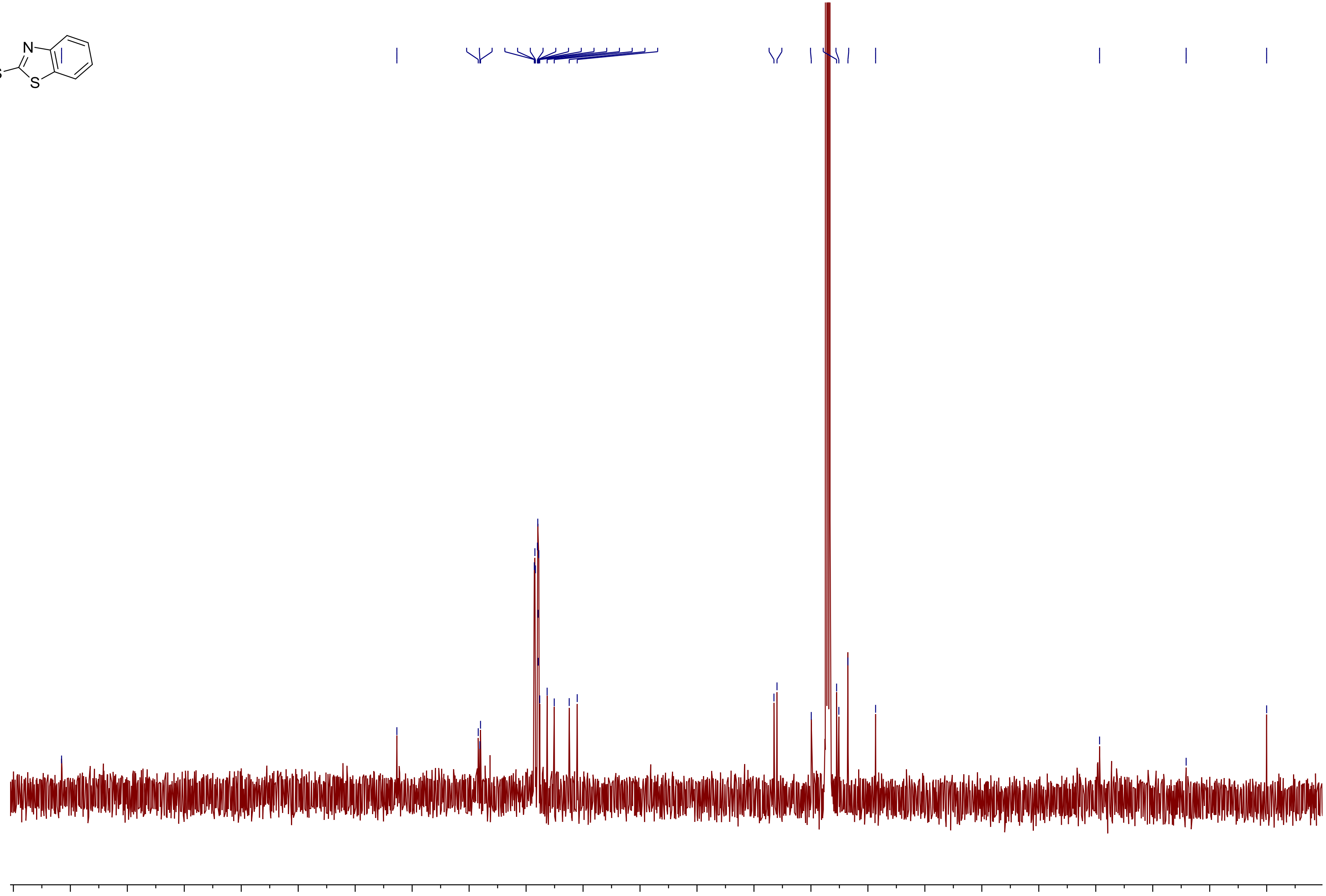

Figure S57. ^{13}C NMR spectrum (400 MHz) of **22** in $CDCl_3$

Phenyl 3,4,6-tri-*O*-benzyl-1-seleno-β-D-glucopyranoside 26

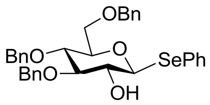

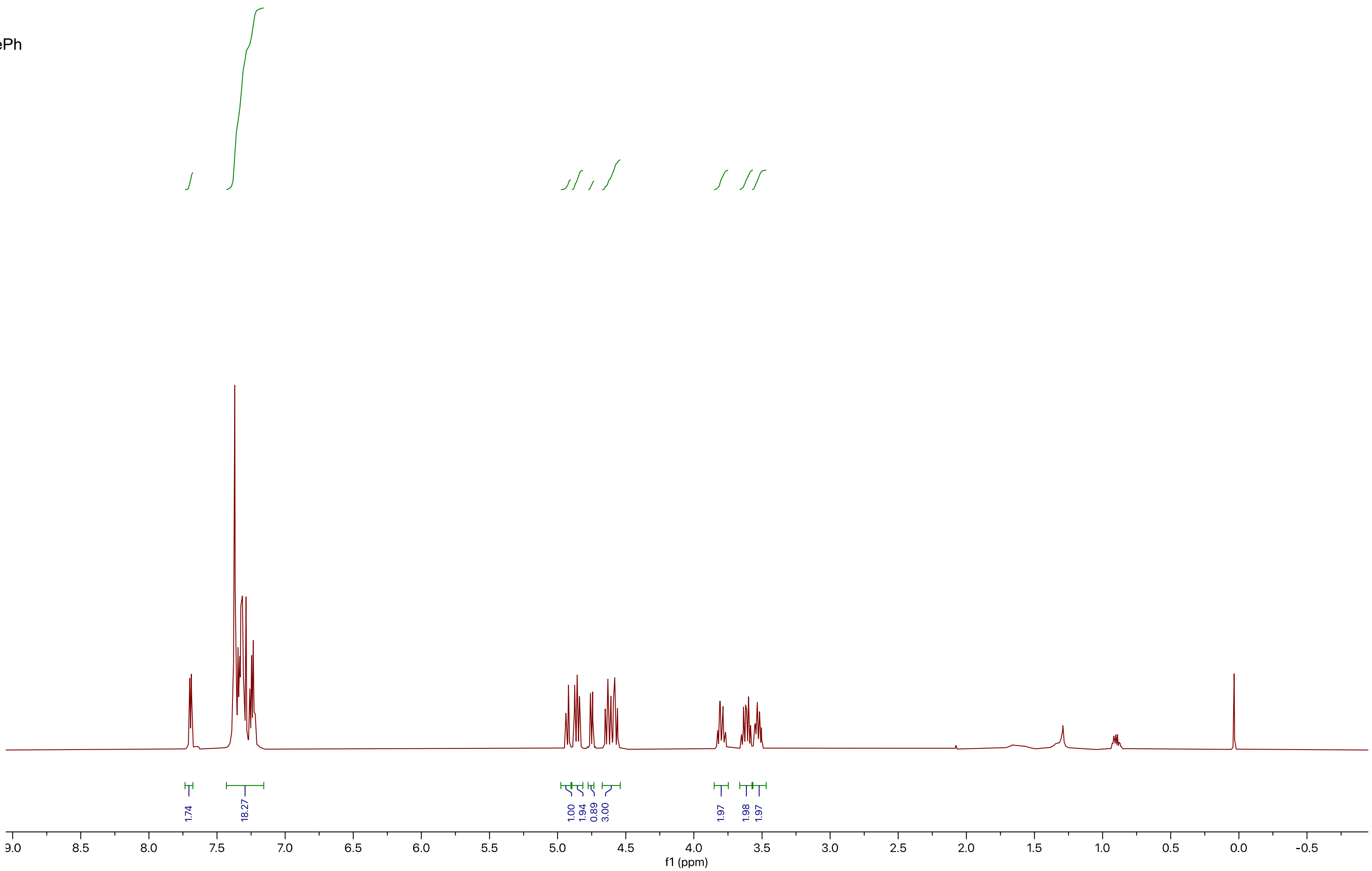

Figure S58. ^{1}H NMR spectrum (400 MHz) of **26** in $CDCl_3$

Phenyl 3,4,6-tri-*O*-benzyl-2-*O*-picolyl-1-thio-β-D-glucopyranoside 28

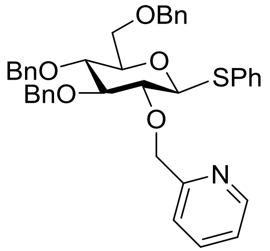

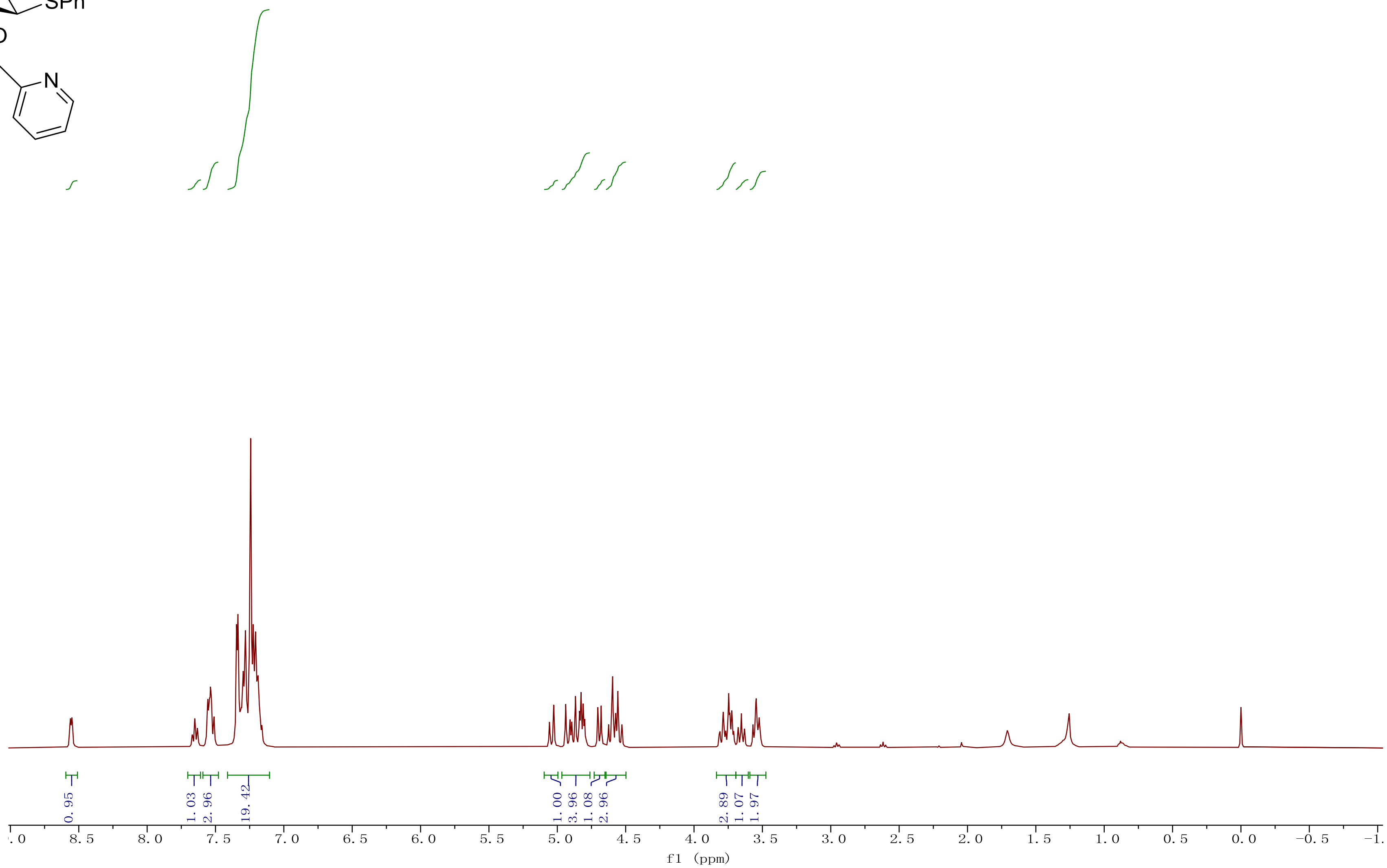

Figure S59. ^{1}H NMR spectrum (400 MHz) of **28** in $CDCl_3$

Phenyl 3,4,6-tri-*O*-benzyl-2-*O*-picolyl-1-thio-β-D-glucopyranoside 28

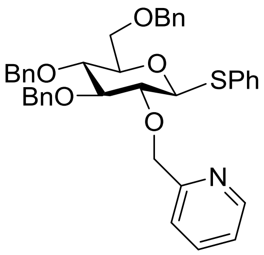

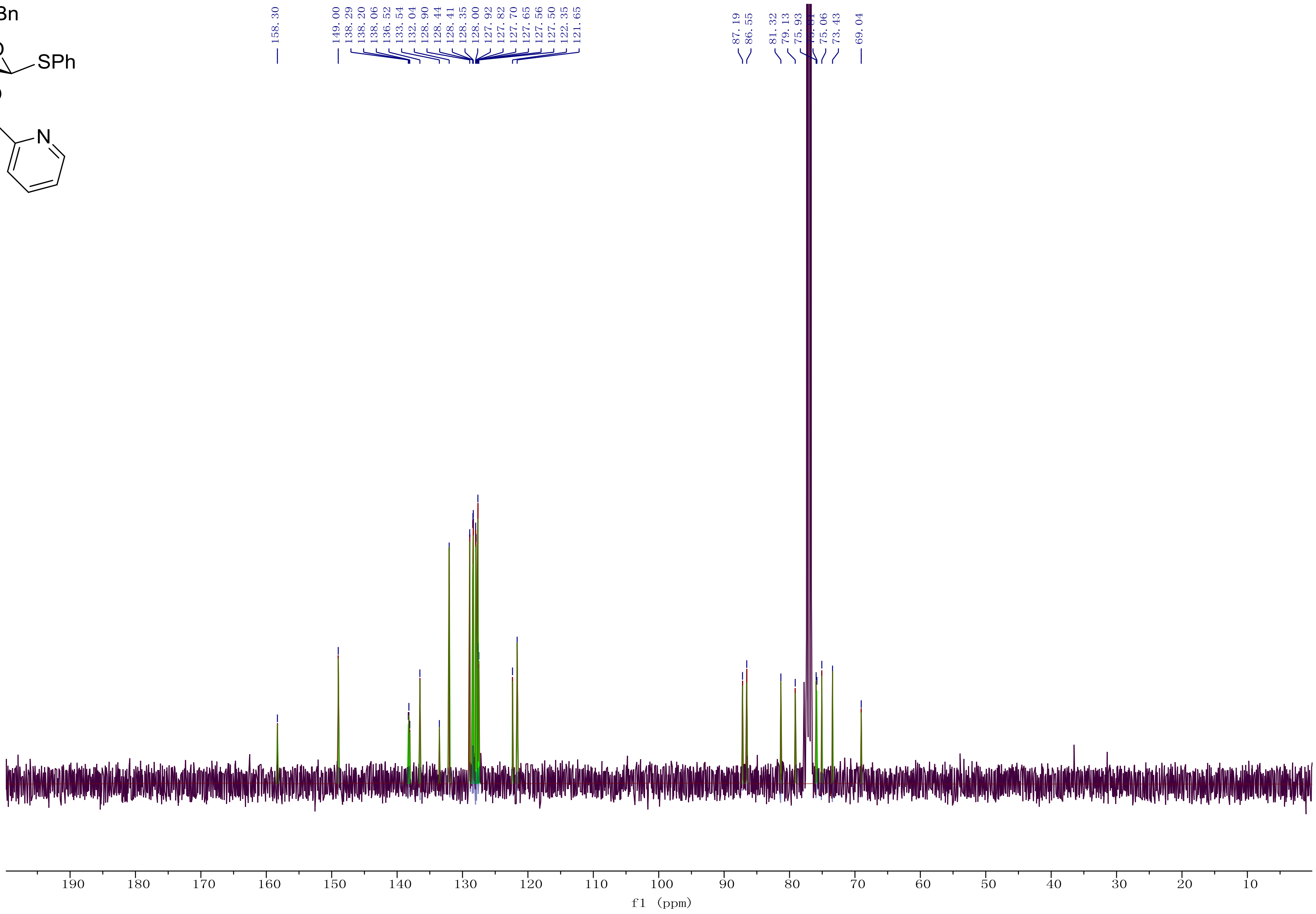

Figure S60. ^{13}C NMR spectrum (100 MHz) of **28** in $CDCl_3$

Phenyl 3,4,6-tri-*O*-benzyl-2-*O*-picolyl-1-thio-β-D-galactopyranoside 29

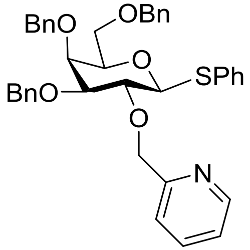

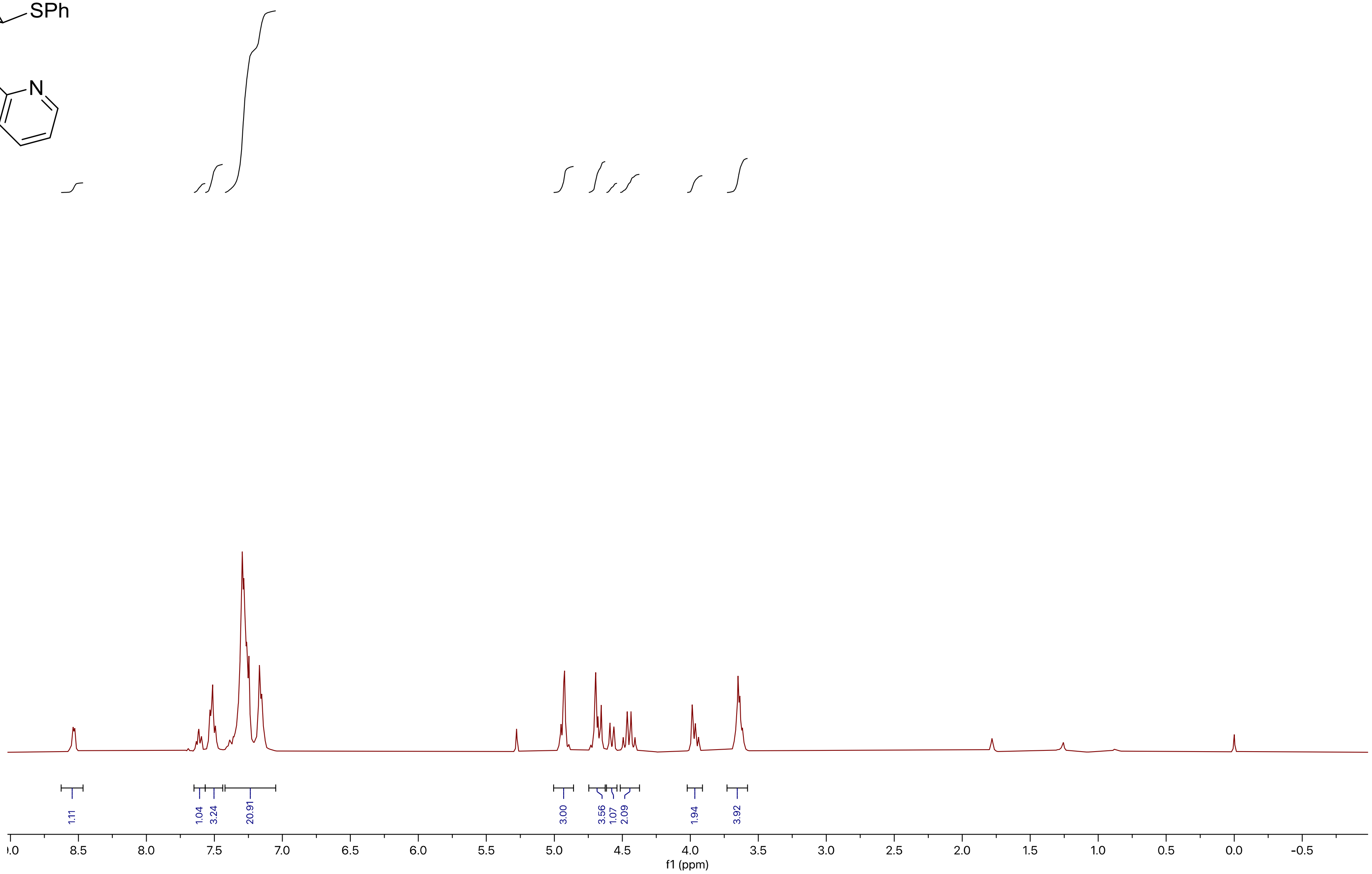

Figure S61. ^{1}H NMR spectrum (400 MHz) of **29** in $CDCl_3$

Phenyl 3,4,6-tri-*O*-benzyl-2-*O*-picolyl-1-thio-β-D-galactopyranoside 29

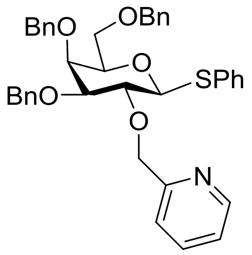

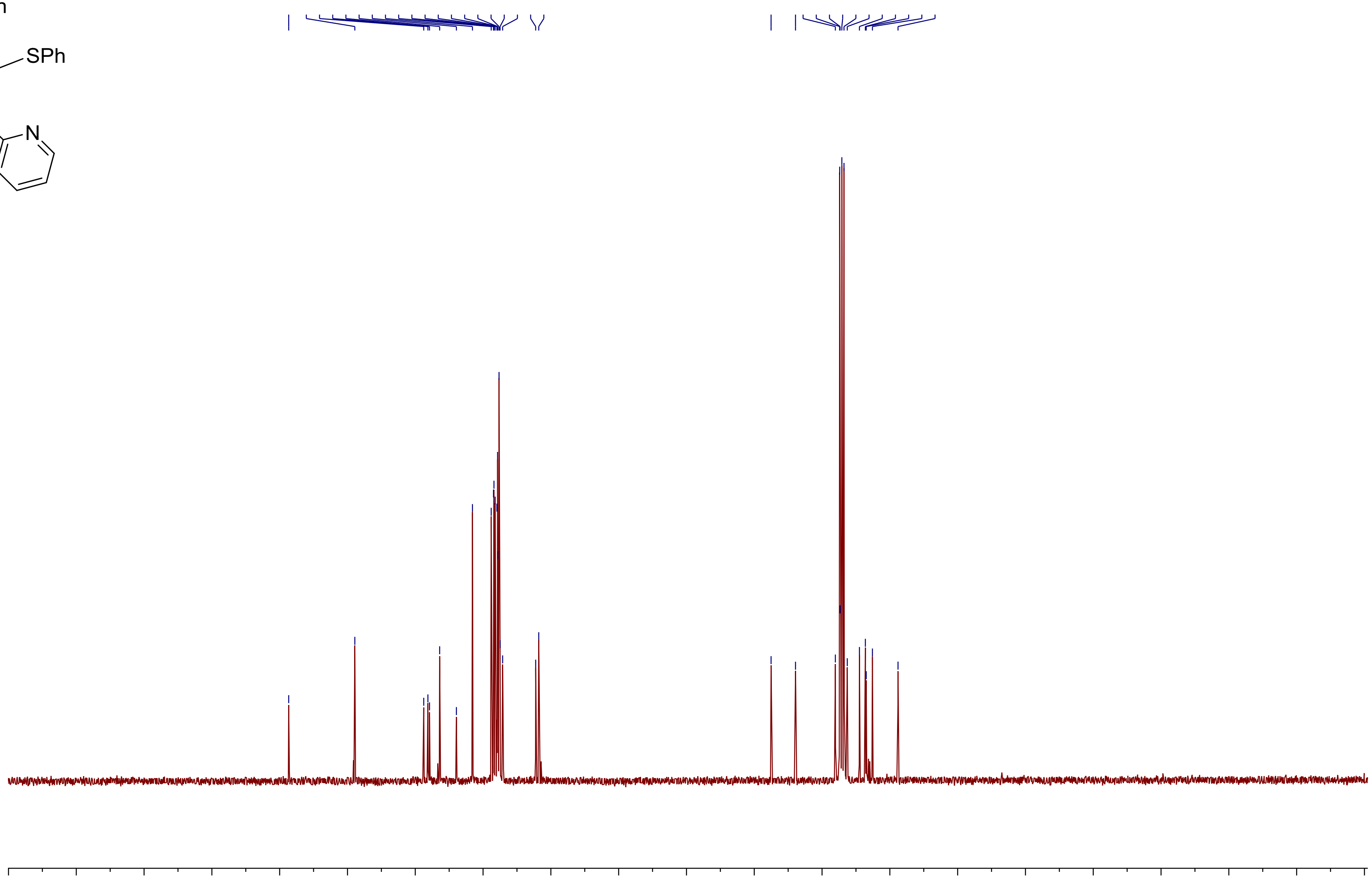

Figure S62. ^{13}C NMR spectrum (100 MHz) of **29** in $CDCl_3$

Phenyl 3,4,6-tri-*O*-benzyl-2-*O*-picolyl-1-thio-β-D-galactopyranoside 29

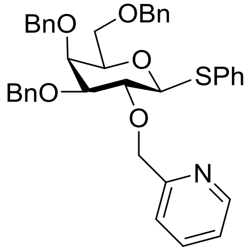

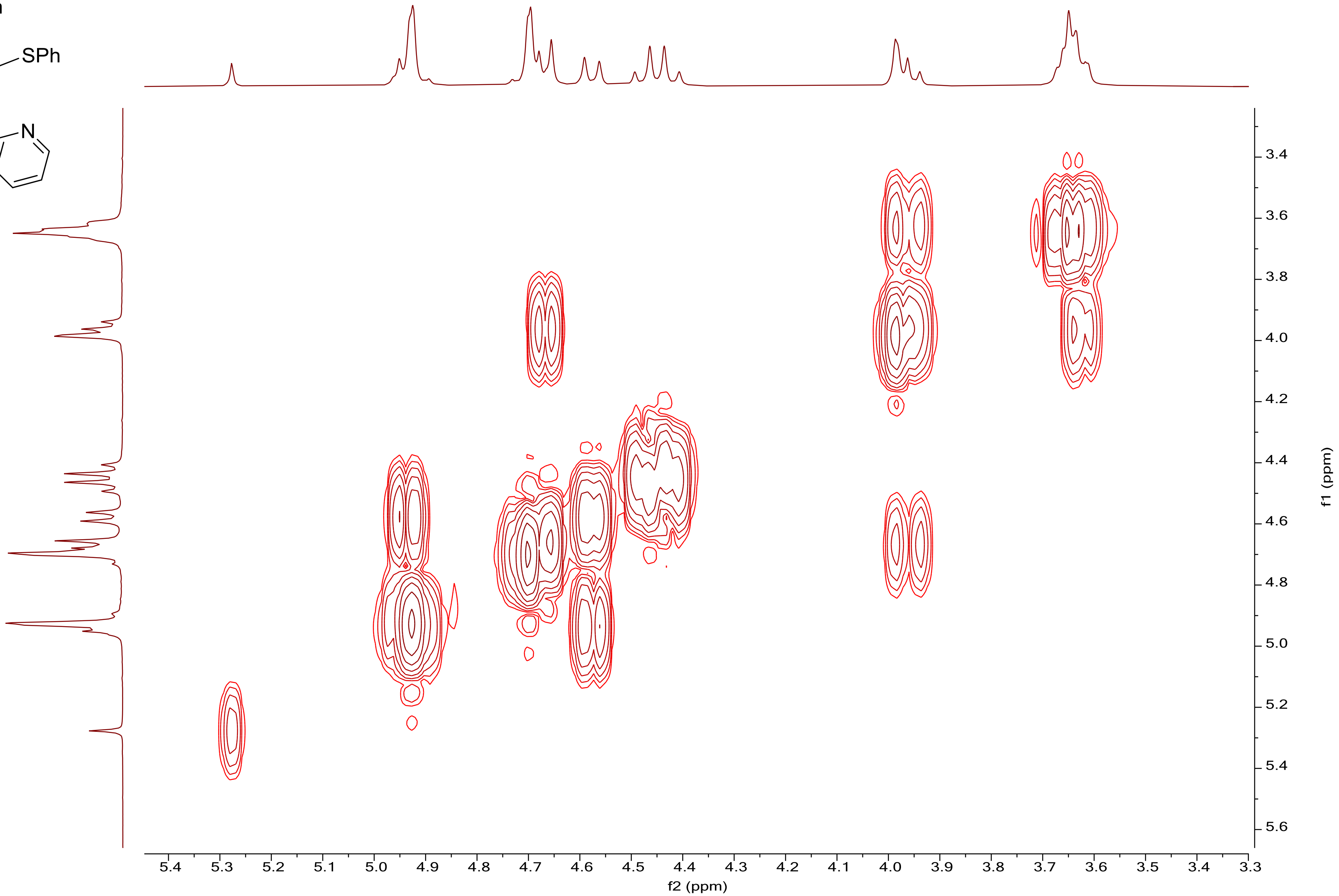

Figure S63. ^1H-^1H COSY spectrum (400 MHz) of **29** in $CDCl_3$

Phenyl 3,4,6-tri-*O*-benzyl-2-*O*-(phenylmethoxy)methyl-β-D-1-thio-glucopyranoside 30

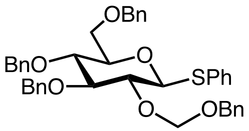

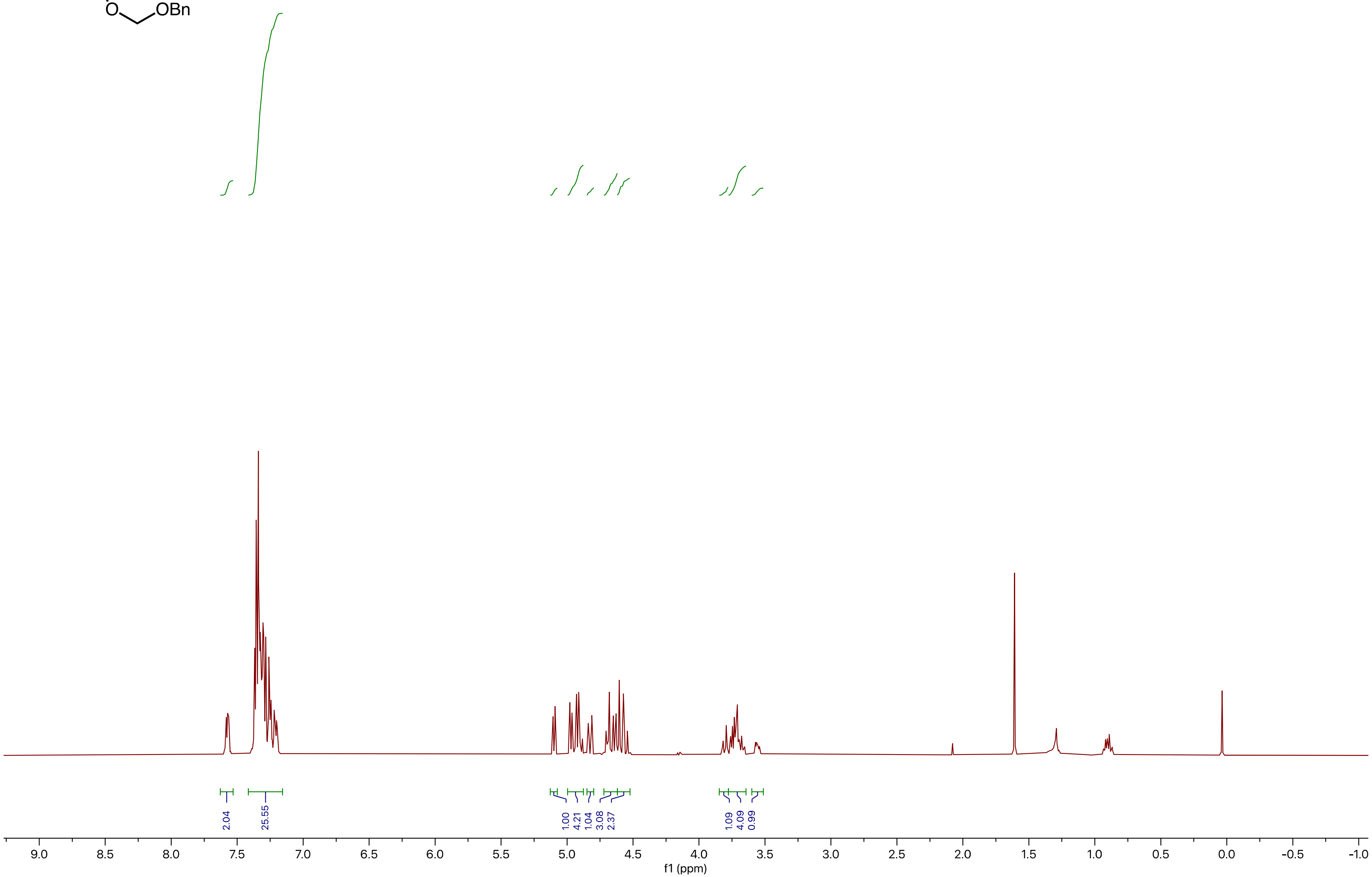

Figure S64. ^{1}H NMR spectrum (400 MHz) of **30** in $CDCl_3$

Phenyl 3,4,6-tri-*O*-benzyl-2-*O*-(cyanomethyl)-1-thio-β-D-glucopyranoside 31

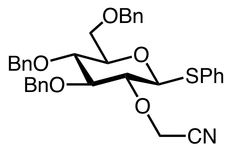

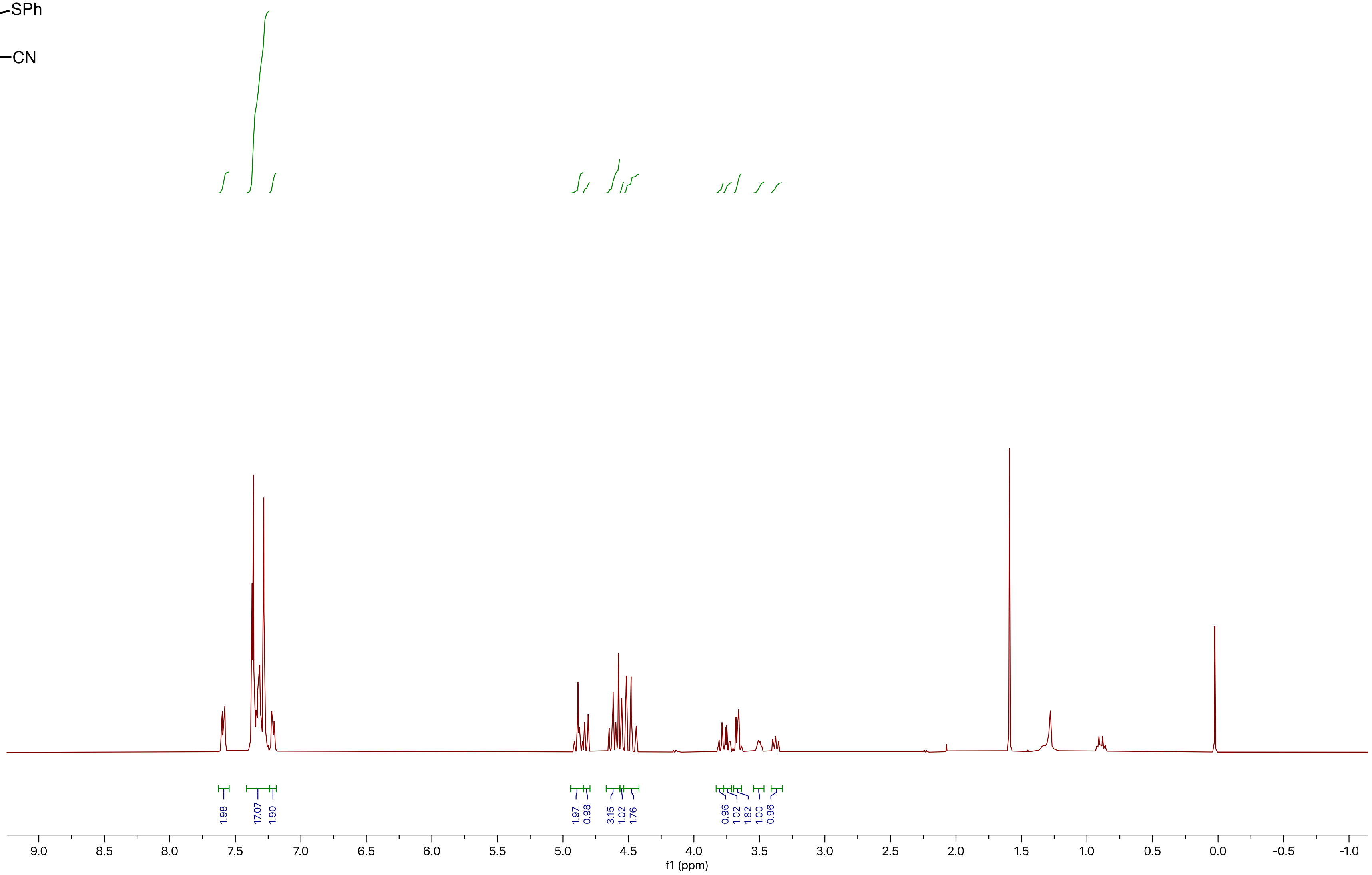

Figure S65. ^{1}H NMR spectrum (400 MHz) of **31** in $CDCl_3$

Phenyl 3,4,6-tri-*O*-benzyl-2-*O*-(2-cyanobenzyl)-1-thio-β-D-glucopyranoside 32

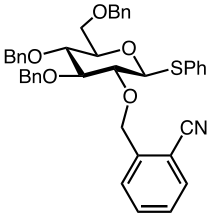

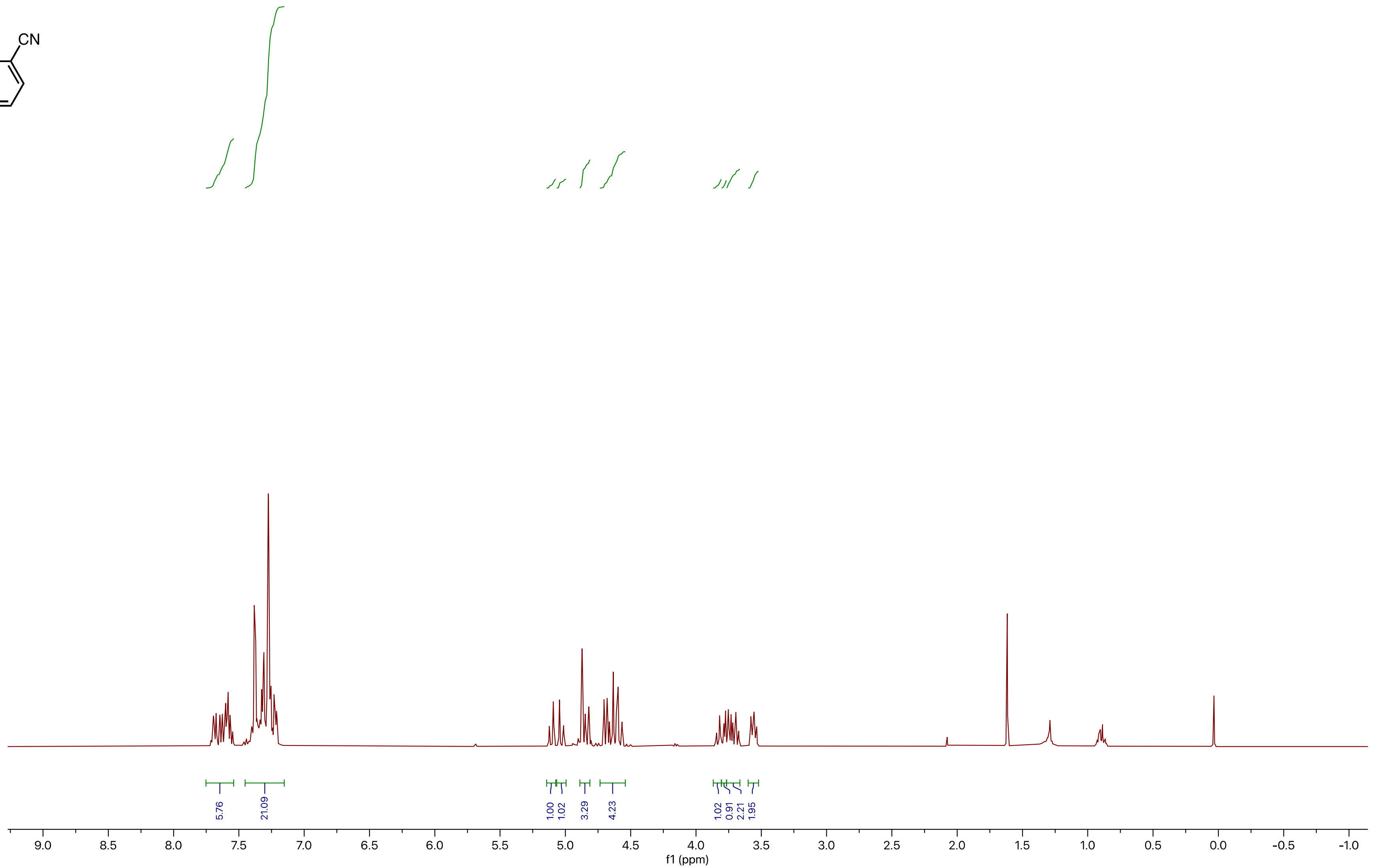

Figure S66. ^{1}H NMR spectrum (400 MHz) of **32** in $CDCl_3$

Phenyl 3,4,6-tri-*O*-benzyl-2-*O*- benzoyl-1-thio-β-D-glucopyranoside 33

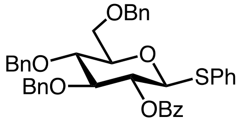

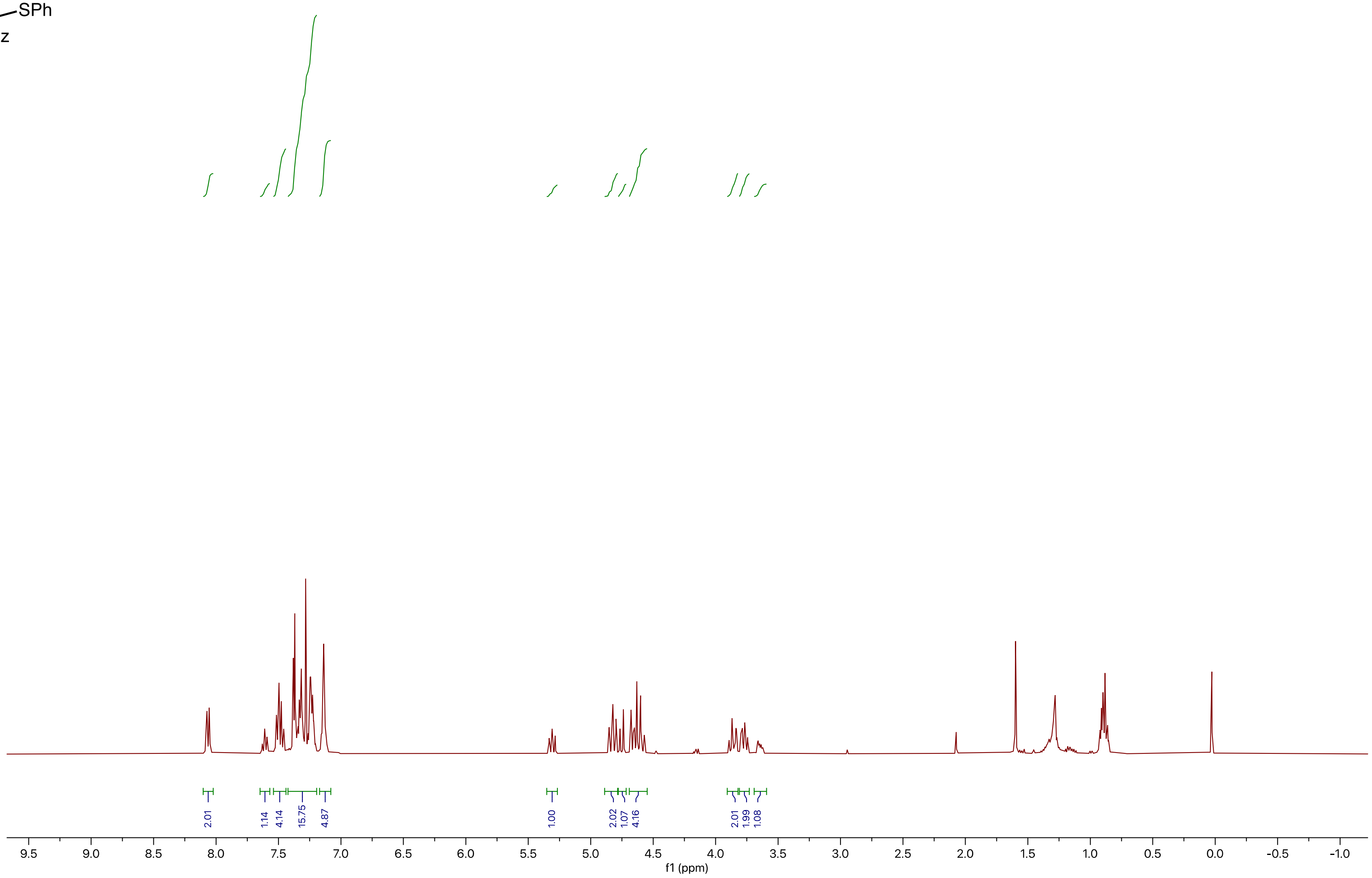

Figure S67. ^{1}H NMR spectrum (400 MHz) of **33** in $CDCl_3$

Phenyl 3,4,6-tri-*O*-benzyl-2-*O*- pivaloyl-1-thio-β-D-glucopyranoside 34

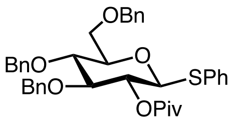

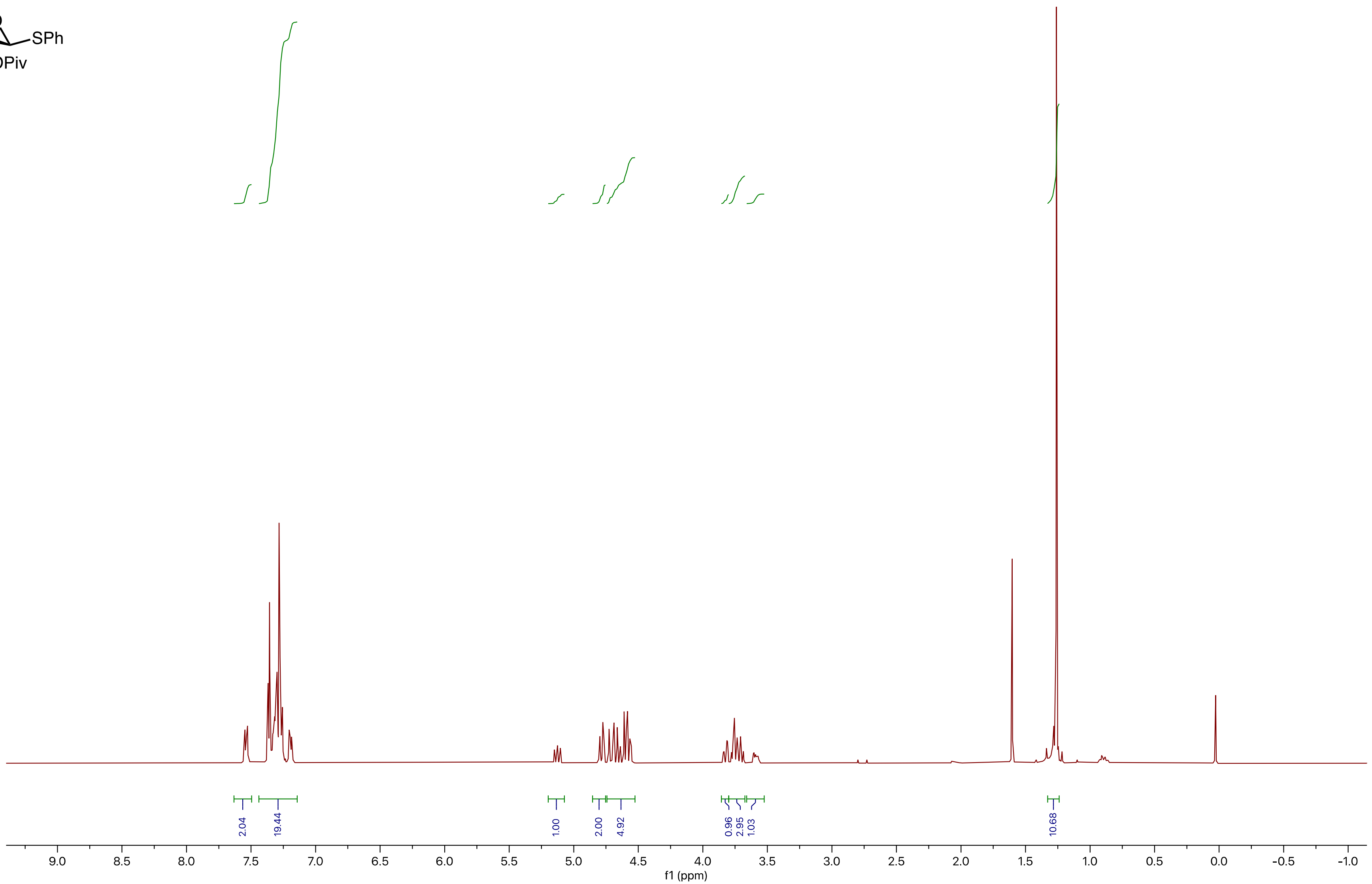

Figure S68. ^{1}H NMR spectrum (400 MHz) of **34** in $CDCl_3$

Methyl 2,3,4-tri-*O*-benzoyl-6-*O*-(3,4,6-tri-*O*-benzyl-2-*O*-picolyl-β-D-glucopyranosyl)-α-D-glucopyranoside 35

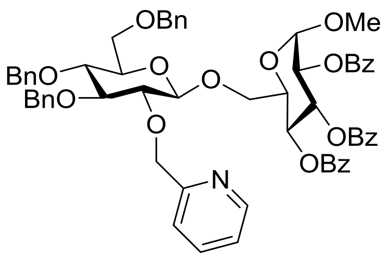

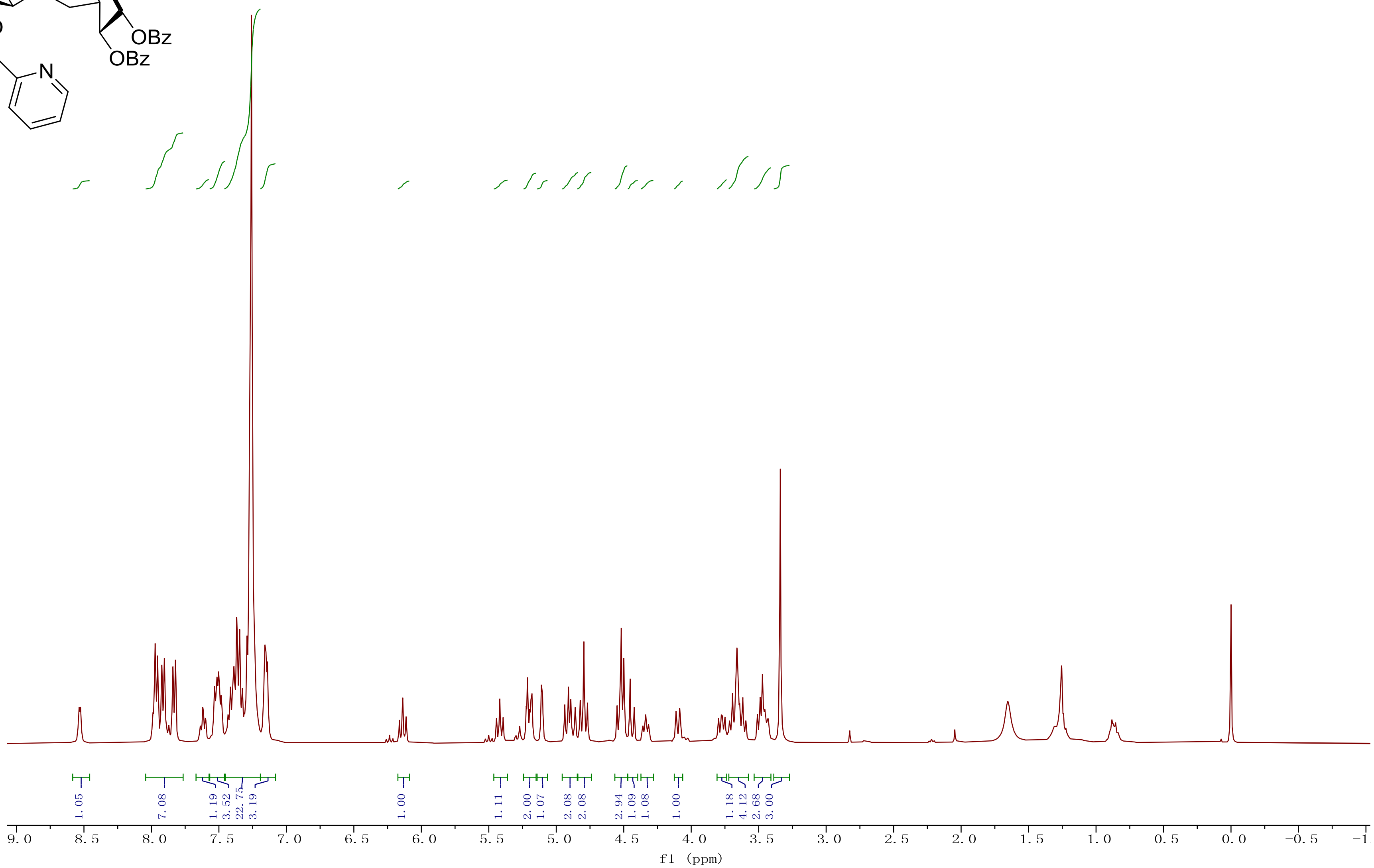

Figure S69. ^{1}H NMR spectrum (400 MHz) of **35** in $CDCl_3$

Methyl 2,3,4-tri-*O*-benzoyl-6-*O*-(3,4,6-tri-*O*-benzyl-2-*O*-picolyl-β-D-glucopyranosyl)-α-D-glucopyranoside 35

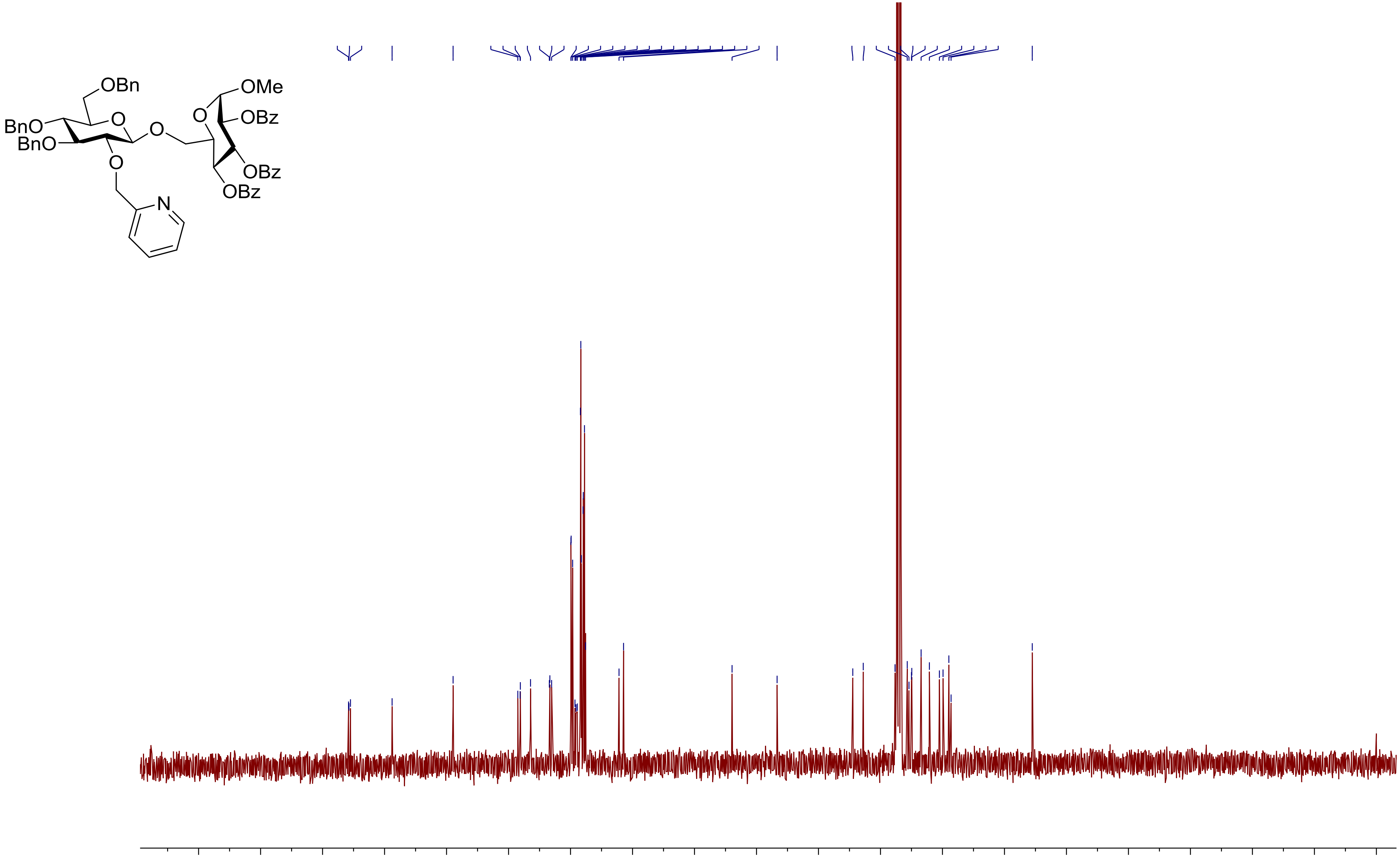

Figure S70. ^{13}C NMR spectrum (100 MHz) of **35** in $CDCl_3$

Methyl 2,3,4-tri-*O*-benzoyl-6-*O*-(3,4,6-tri-*O*-benzyl-2-*O*-picolyl-β-D-glucopyranosyl)-α-D-glucopyranoside 35

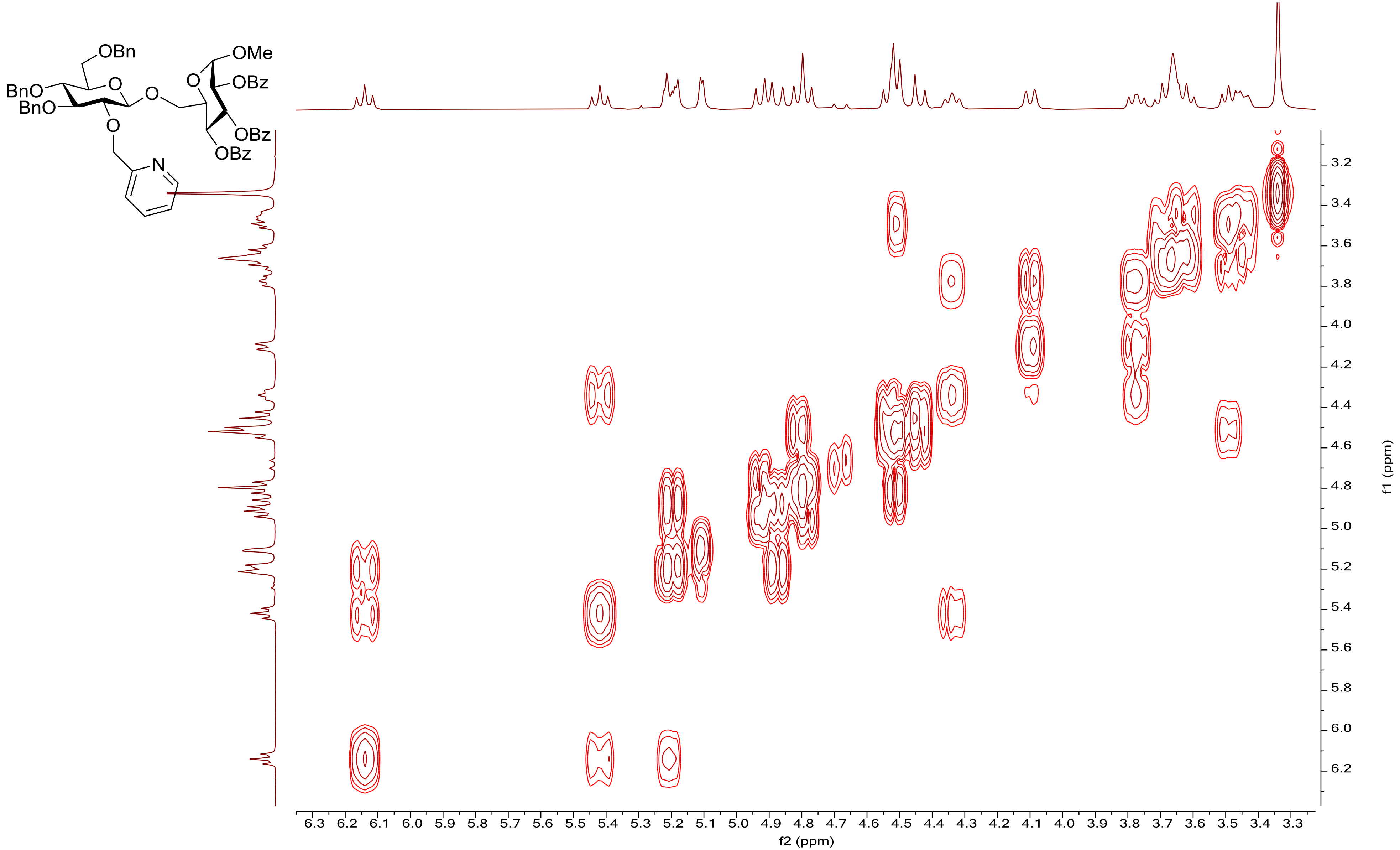

Figure S71. ^{1}H-^{1}H COSY spectrum (100 MHz) of **35** in $CDCl_3$

Methyl 2,3,4-tri-*O*-benzyl-6-*O*-(3,4,6-tri-*O*-benzyl-2-*O*-picolyl-β-D-glucopyranosyl)-α-D-glucopyranoside 36

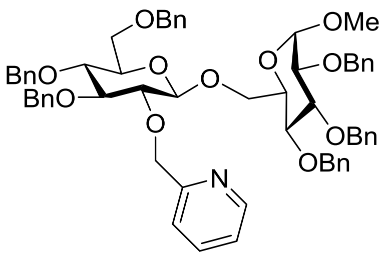

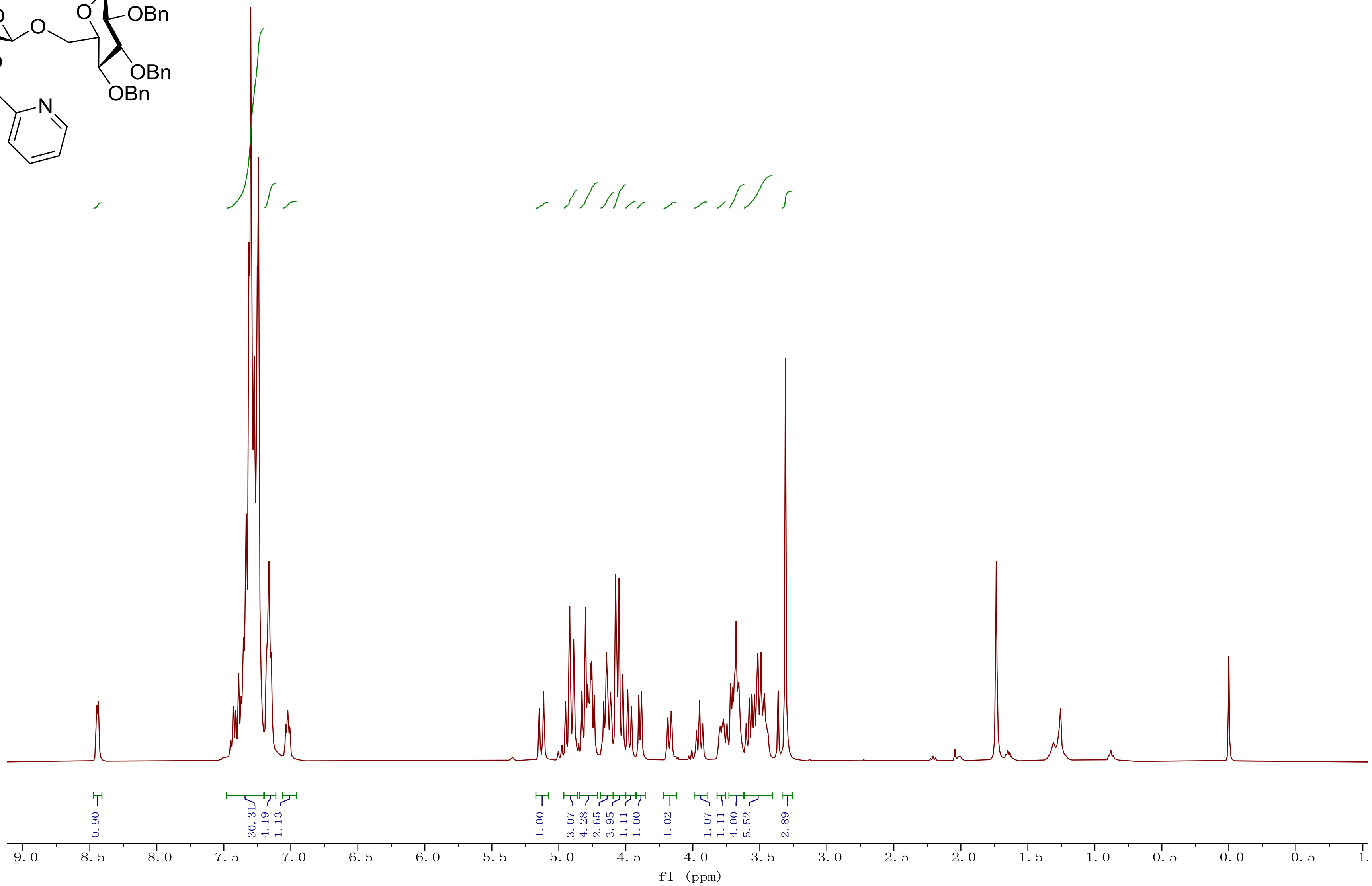

Figure S72. ^{1}H NMR spectrum (400 MHz) of **36** in $CDCl_3$

Methyl 2,3,6-tri-*O*-benzyl-4-*O*-(3,4,6-tri-*O*-benzyl-2-*O*-picolyl-β-D-glucopyranosyl)-α-D-glucopyranoside 37

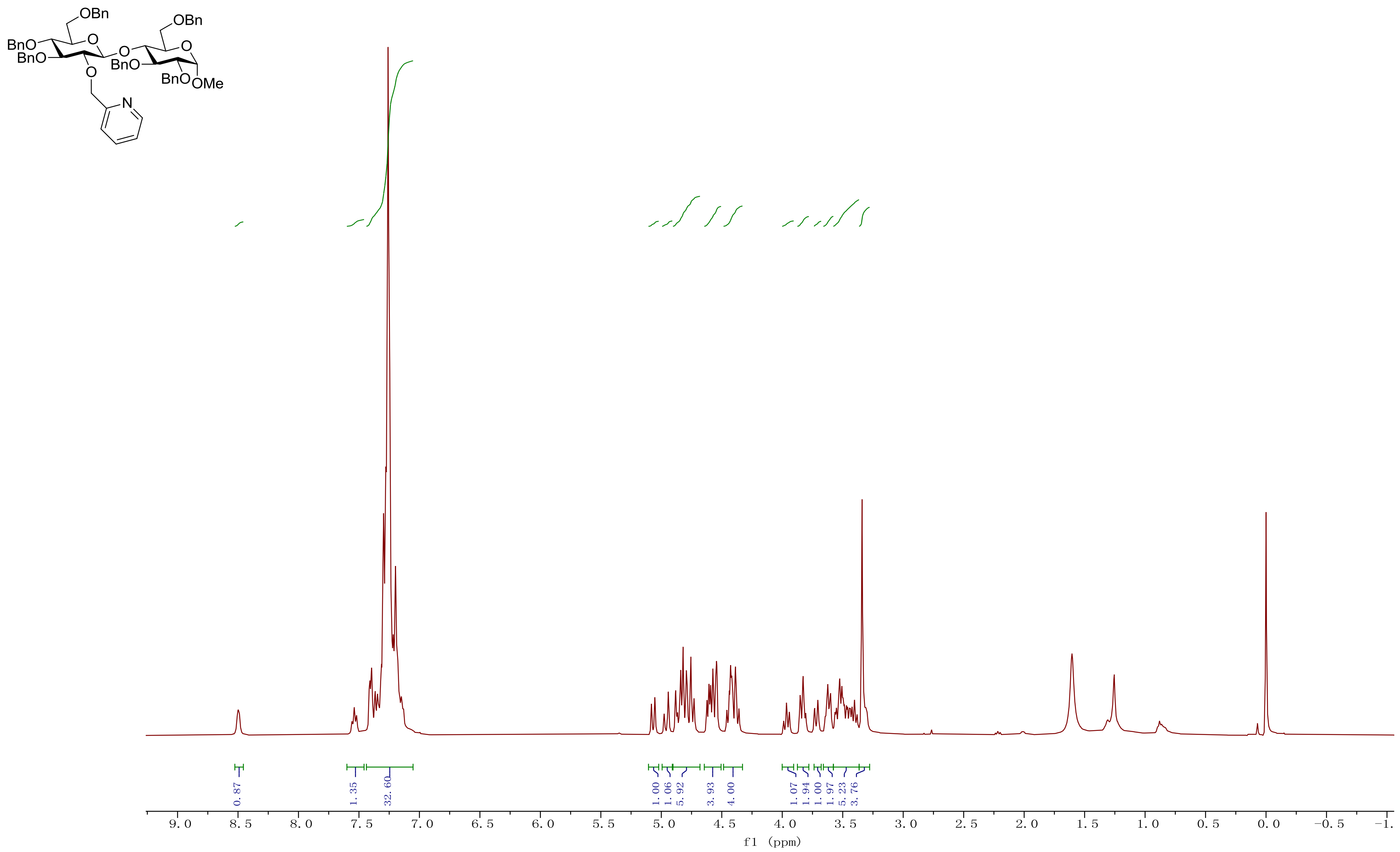

Figure S73. ^{1}H NMR spectrum (400 MHz) of **37** in $CDCl_3$

Methyl 2,4,6-tri-*O*-benzyl-3-*O*-(3,4,6-tri-*O*-benzyl-2-*O*-picolyl-β-D-glucopyranosyl)-α-D-glucopyranoside 38

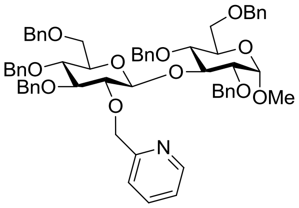

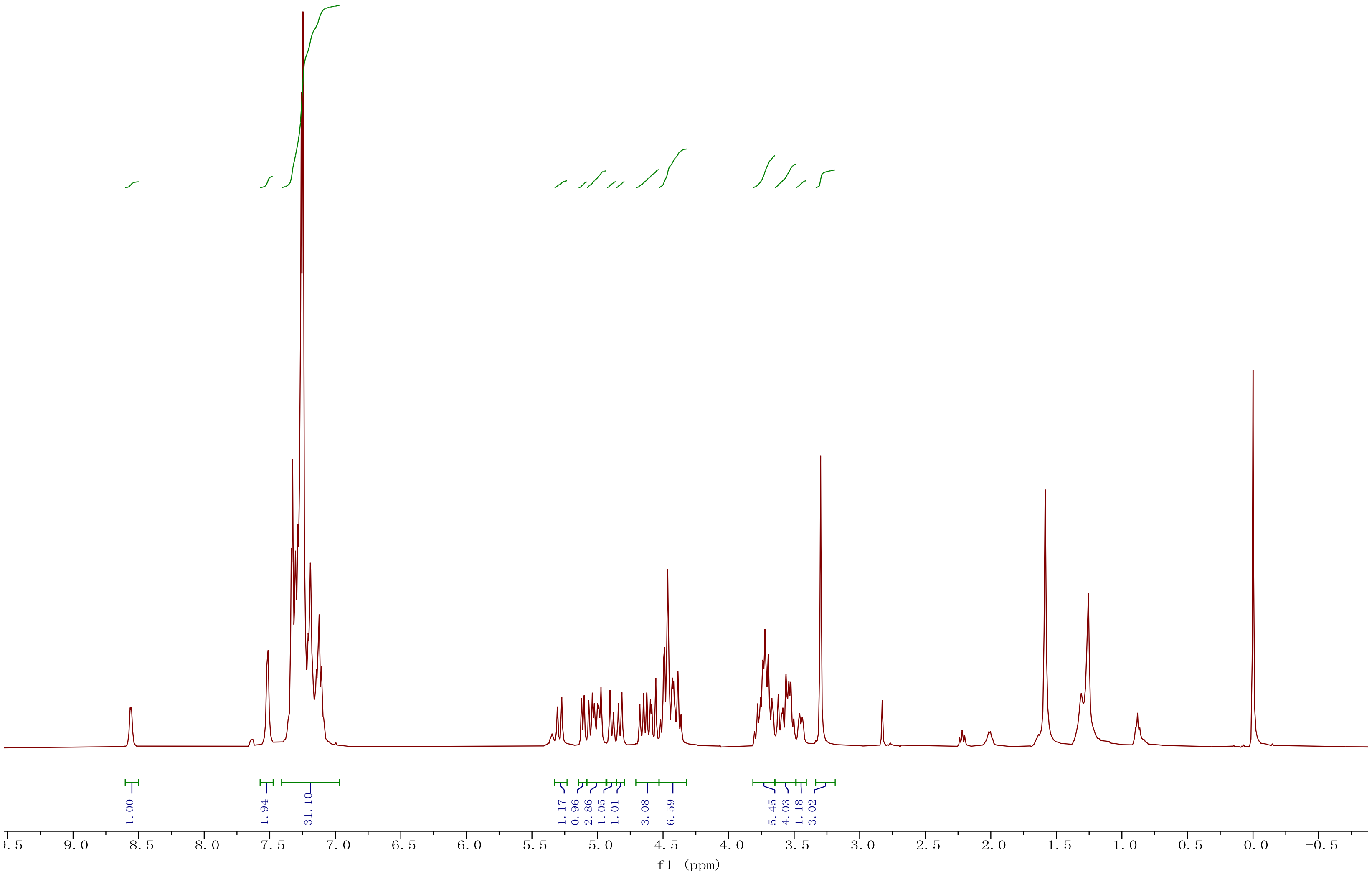

Figure S74. ^{1}H NMR spectrum (400 MHz) of **38** in $CDCl_3$

Methyl 3,4,6-tri-*O*-benzyl-2-*O*-(3,4,6-tri-*O*-benzyl-2-*O*-picolyl-β-D-glucopyranosyl)-*α*-D-glucopyranoside 39

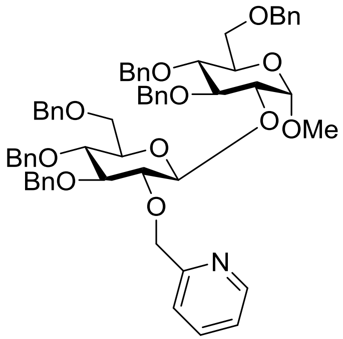

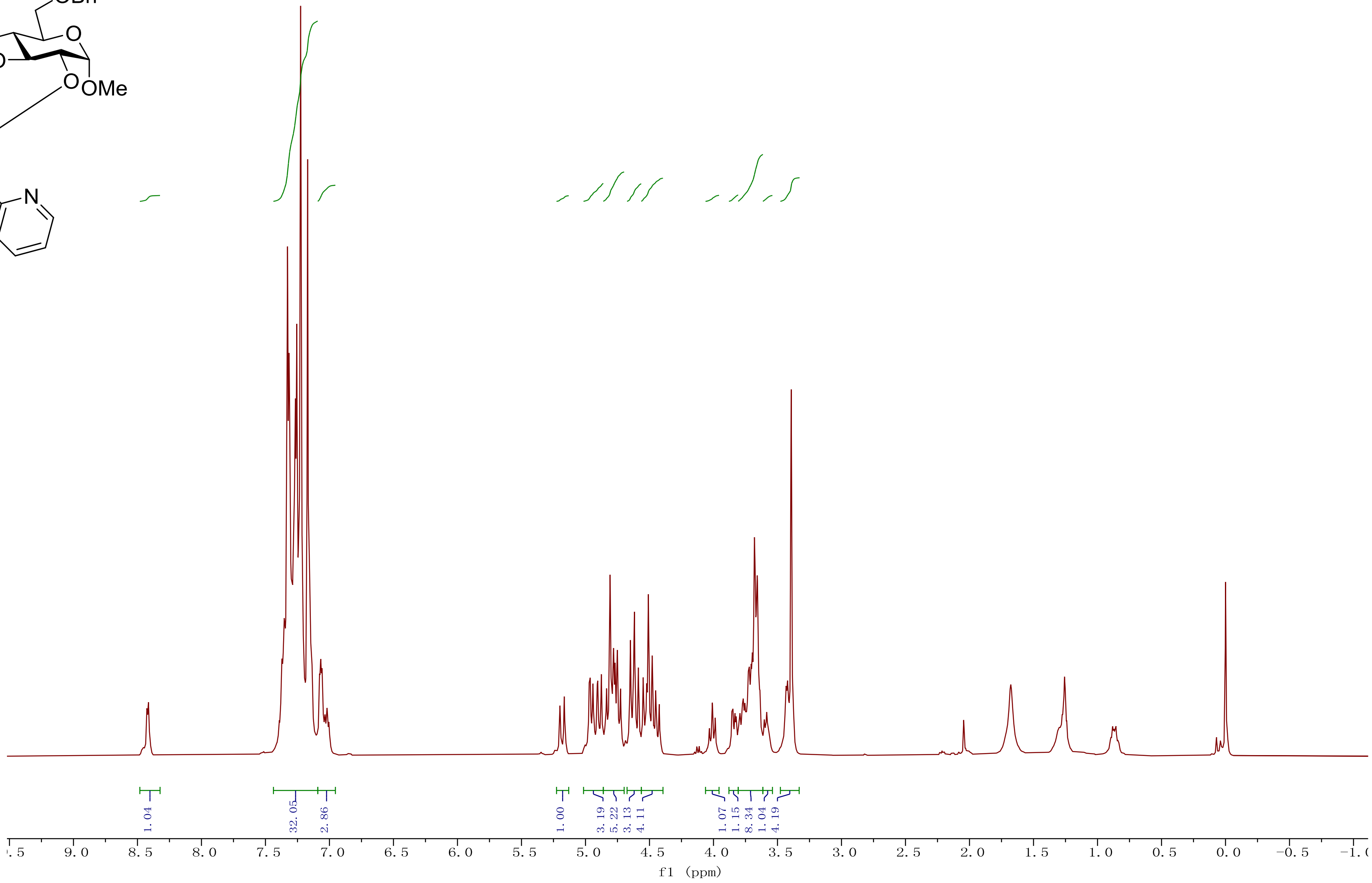

Figure S75. ^{1}H NMR spectrum (400 MHz) of **39** in $CDCl_3$

1,2:5,6-Di-*O*-isopropylidine-3-*O*-(3,4,6-tri-*O*-benzyl-2-*O*-picolyl-β-D-glucopyranosyl)-α-D-glucofuranose 40

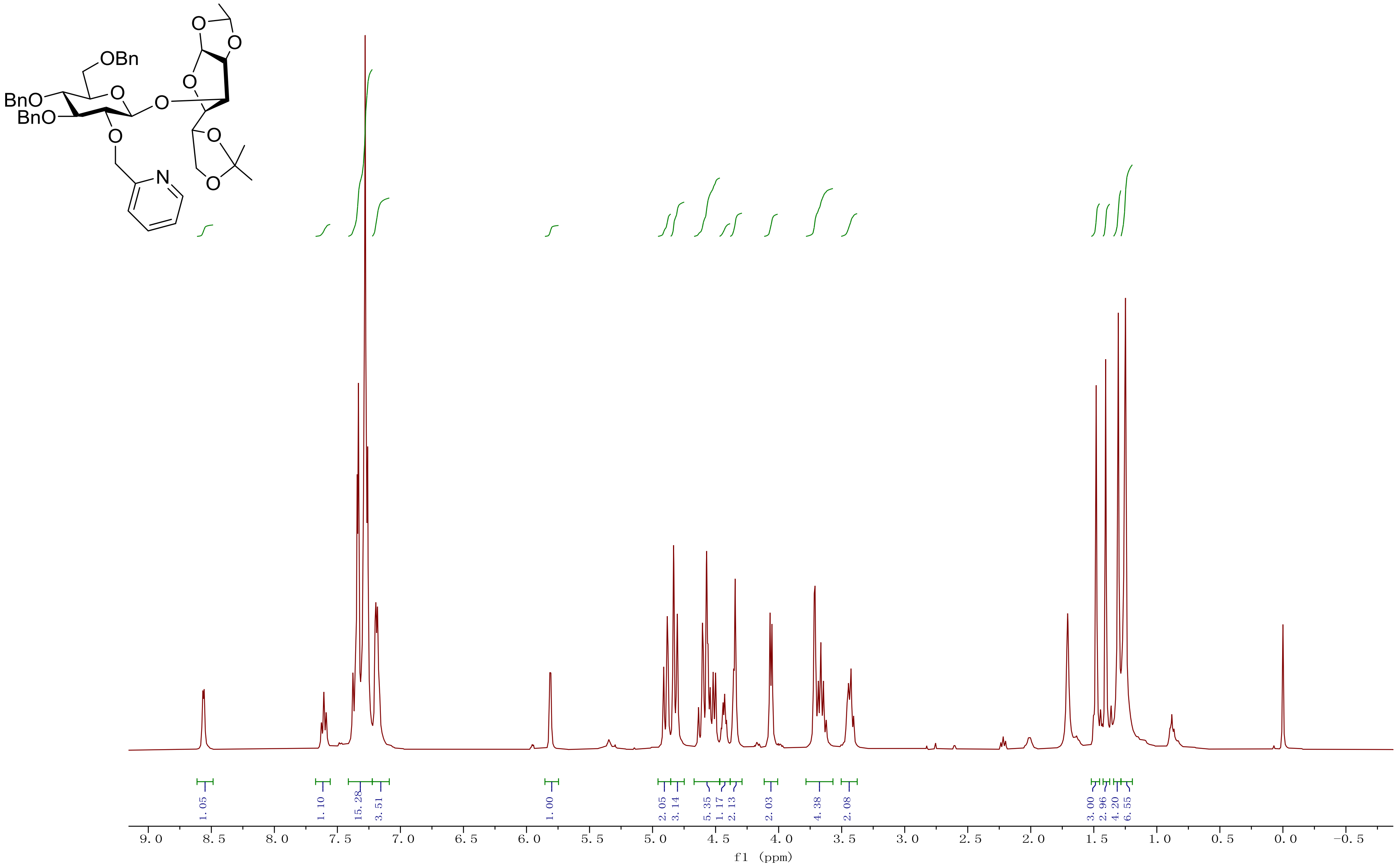

Figure S76. ^{1}H NMR spectrum (400 MHz) of **40** in $CDCl_3$

Methyl 2,3,4-tri-*O*-benzyl-6-*O*-(3,4,6-tri-*O*-benzyl-2-*O*-picolyl-β-D-galactopyranosyl)-α-D-glucopyranoside 41

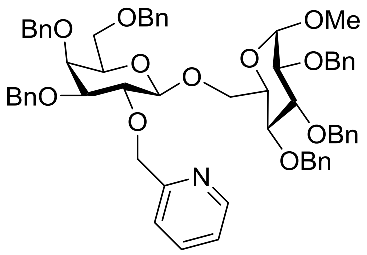

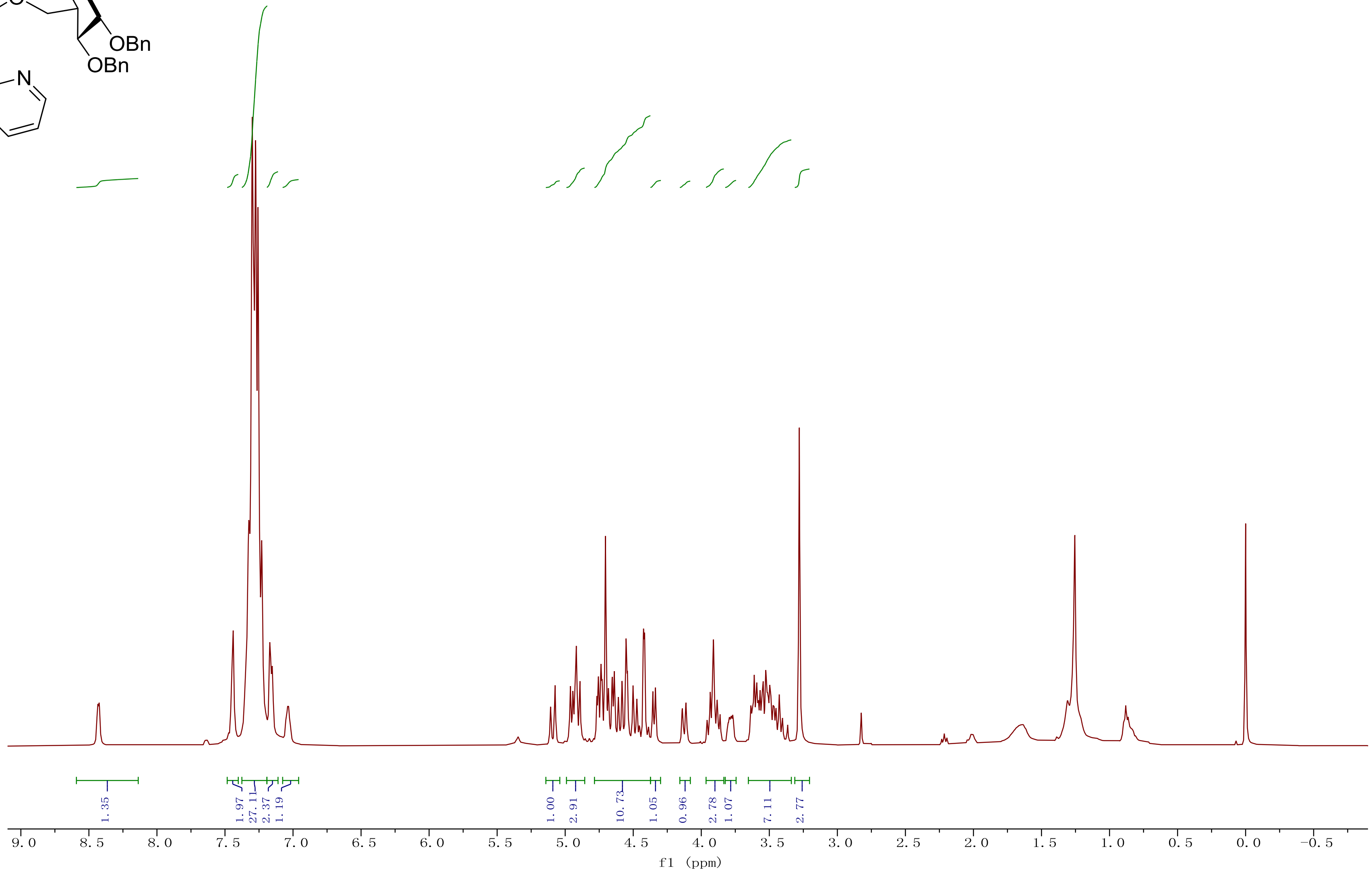

Figure S77. ^{1}H NMR spectrum (400 MHz) of **41** in $CDCl_3$
